# A statistical method for single sample analysis of HumanMethylation450 array data: genome-wide methylation analysis of patients with imprinting disorders

**DOI:** 10.1186/s13148-015-0081-5

**Published:** 2015-04-21

**Authors:** Faisal I Rezwan, Louise E Docherty, Rebecca L Poole, Gabrielle A Lockett, S Hasan Arshad, John W Holloway, I Karen Temple, Deborah JG Mackay

**Affiliations:** Human Development and Health, Faculty of Medicine, University of Southampton, Tremona Road, Southampton, Hampshire SO16 6YD UK; Wessex Regional Genetics Laboratory, Salisbury NHS Foundation Trust, Salisbury District Hospital, Salisbury, Wilts SO2 8BJ UK; The David Hide Asthma and Allergy Research Centre, St Mary’s Hospital, Newport, Isle of Wight PO30 5TG UK; Clinical and Experimental Sciences, Faculty of Medicine, University of Southampton, Tremona Road, Southampton, Hampshire SO16 6YD UK; Wessex Clinical Genetics Service, Princess Anne Hospital, University Hospital Southampton NHS Foundation Trust, Southampton, UK

**Keywords:** Methylation, Illumina HumanMethylation450 array, Single case-control analysis, Crawford-Howell *t*-test

## Abstract

**Background:**

The Illumina Infinium HumanMethylation450 BeadChip is an array-based technology for analysing DNA methylation at approximately 475,000 differentially methylated cytosines across the human genome. Hitherto, the array has been used for case-control studies, where sample numbers can be sufficient to yield statistically robust data on a genome-wide basis. We recently reported an informatic pipeline capable of yielding statistically and biologically significant results using only five cases, which expanded the use of this technology to rare disease studies. However, the clinical application of these technologies requires the ability to perform robust analysis of individual patients.

**Results:**

Here we report a novel informatic approach for methylation array analysis of single samples, using the Crawford-Howell *t*-test. We tested our approach on patients with ultra-rare imprinting disorders with aberrant DNA methylation at multiple locations across the genome, which was previously detected by targeted testing. However, array analysis outperformed targeted assays in three ways: it detected loci not normally analysed by targeted testing, detected methylation changes too subtle to detect by the targeted testing and reported broad and consistent methylation changes across genetic loci not captured by point testing.

**Conclusions:**

This method has potential clinical utility for human disorders where DNA methylation change may be a biomarker of disease.

**Electronic supplementary material:**

The online version of this article (doi:10.1186/s13148-015-0081-5) contains supplementary material, which is available to authorized users.

## Background

Epigenetic modulation of gene expression is responsible for tissue specific and temporal changes across growth and development. The most widely studied of these epigenetic modifications is DNA methylation of 5-methylcytosine at CpG dinucleotides. Aberrations of DNA methylation are associated with a range of diseases, including imprinting disorders and cancer [1]. Recent advances in technologies have made it possible to study the epigenetic changes associated with these diseases using robust genome-wide technologies including the Infinium HumanMethylation450 BeadChip (henceforward denoted the 450 k array; www.Illumina.com). The 450 k array measures the intensity of fluorescent signal from methylated and unmethylated probes at approximately 475,000 CpG dinucleotides across the genome, including CpG islands, promoters, gene bodies, intergenic regions and the majority of imprinted loci. These intensities are then used to calculate DNA methylation levels, with advantageous throughput, cost, coverage and technical consistency.

To date, many studies, utilising the 450 k array, have used case-control designs [2-6]. The limitation to the majority of these studies is that the bioinformatic analysis used requires a large number of cases and controls to obtain statistically significant results. Recently, we developed a novel informatic pipeline yielding statistically and biologically significant results using small case number analysis (case = 5) [7], which expanded the use of this technology to rare disease studies. However, the clinical application of these technologies requires the ability to perform robust analysis of individual patients.

Humans harbour approximately 100 known imprinted genes, characterised by the epigenetic control of gene expression, often through parent-of-origin-specific methylation that is applied in the germ line and conserved through subsequent development in all tissues. As yet, disruption of the methylation state at eight imprinted loci has been associated with imprinting disorders (Beckwith-Wiedemann syndrome (BWS; MIM #130659), Silver-Russell syndrome (SRS; MIM #180860), transient neonatal diabetes mellitus (TNDM; MIM #601410), Prader-Willi syndrome (PWS; MIM #176270), Angelman syndrome (AS; MIM #105830), matUPD14-like (Temple syndrome) and patUPD14-like (Wang-Kagami) syndromes and pseudohypoparathyroidism 1B (PHP-1B; MIM #103580)). Rare patients with multi-locus methylation disorders (MLMD) [8-11] form a uniquely informative group of samples that can be used to develop a sensitive and specific single sample 450 k array bioinformatic pipeline. Informatically, there are a number of approaches to single case analysis. A single normalised sample can be compared against a large sample group standardised in the same way [12]. However, collecting a large normative sample can be both time-consuming and challenging [13]. Another approach is to compare one or more tests to the performance of the same individuals by chi-square tests. However, the significant raw difference between different performances (or scores) can be diminished by comparison against control performance (or score) and vice versa. Alternatively, a single sample’s performance (or score) can be compared to that of a matched control group. Whereas the standardised method requires a large number of samples and intra-individual comparisons require assessment of two or more independent variables, the single case-control method requires only a moderate number of controls [12].

In single case-control analysis, the most common means of detecting significant differences is to convert the case’s score to a z-score using the control sample mean and standard deviation and referring the score to a table of areas under the normal curve [14]. However, this might not accurately estimate the parameters if the control sample is large enough to assume that the mean and the standard deviation are used as population parameter rather than sample statistics [15]. In many cases, the number of controls can be quite small (even smaller than 10). Therefore, it is logical to use a *t*-test method using the *t*-distribution. A number of studies used one-sample *t*-tests in their single case-control studies, and to date, several studies used Crawford and Howell’s *t*-test methods as mentioned in [16]. The Weisberg *t*-test, for identifying outliers, is also capable of single sample analysis. The different *t*-tests will be briefly described in the following section.

However, all of these studies involved neuropsychological rather than 450 k array data. Here we demonstrate the effectiveness of a single case-control method for analysing 450 k array data from patients with multi-locus and single-locus imprinting disorders. Using 450 k array data from patients with known regions and severity of DNA hypomethylation, we were able to optimise our informatic approach: firstly, by comparison of various *t*-test methods and secondly, by varying the control group size to identify the smallest control size required to detect biologically and statistically significant changes in methylation at known regions of hypomethylation specific to each patient.

## Results

As mentioned earlier, we developed a pipeline for small sample size (*n*_cases_ = 5) against large control groups, using patients with TND-MLMD and BWS-MLMD and broadly similar patterns of methylation change as determined by targeted testing. The pipeline applied a linear model as the statistical method, and CpGs were selected where they were hypomethylated compared with controls, with an adjusted *P* value < 1.33 × 10^− 7^ and *M* values between −1 and +1 (equivalent to 0.26 ≤ *β* ≤ 0.7) in normal controls, to enrich for the intermediate methylation consistent with the hemimethylation of genomic imprinting. We focused our attention on genes or DNA regions containing at minimum two CpGs within 2000 nucleotides. Using this approach, we detected 21 hypomethylated regions in the TND-MLMD and 34 regions in BWS-MLMD pooled samples [7], including regions of hypomethylation that were previously unknown and consistent with genomic imprinting. Targeted testing showed that some of these regions were not hypomethylated in all the samples. Therefore, though analysis of small case numbers vs. large control numbers could identify differential methylation robustly, it failed to identify patient-specific regions without targeted follow-up testing.

We applied single sample *t*-tests (Crawford-Howell, Weisberg and one-sample *t*-tests) instead of linear regression as the statistical method and modified the filtration criteria: hypomethylated DNA sequences with characteristics consistent with imprinting were selected as those containing a minimum of three consecutive CpGs within 2000 nucleotides with *M* value between −1 and +1 in normal controls and *P* value <0.05.

### Selection of the CH *t*-test after comparative evaluation of *t*-test performance

Here we compared three types of *t*-tests (Crawford-Howell, one-sample and Weisberg *t*-tests) for their ability to identify known regions of differential methylation by single sample analysis while predicting less variability using a randomly selected control group size of 50 which was batch-matched, that is derived from the same batch of 450 k analyses as the patient DNA.

**Figure 1:**
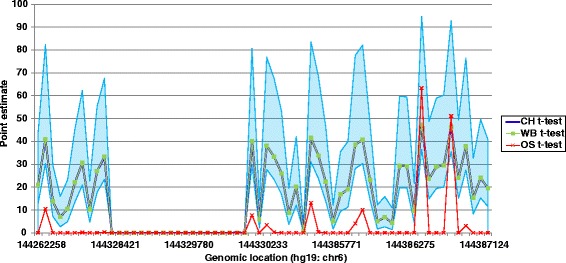
The performance of three *t*-tests (one-sample, Weisberg and Crawford-Howell *t*-tests) around the *PLAGL1* region in TND-MLMD 1. The x-axis denotes the genomic location of *PLAGL1* on chromosome 6. The y-axis represents the estimated percentage of the control population that would be expected to obtain lower score than the case (point estimate), which is calculated according to the one-sample (OS, red crossed line), Weisberg (WB, green line with green square markers) and Crawford-Howell (CH, blue line) methods. The blue shade represents 95% confidence interval of the point estimates from the noncentrality parameter from a noncentral *t*-distribution.

Using simulated data, it has already been shown that the Crawford-Howell *t*-test (denoted as CH *t*-test henceforth) works better than the one-sample *t*-test irrespective of the number of controls and the one-sample *t*-test has a high Type I error rate [16]. It is true that, in case of single sample *t*-test, using single value against a control group is highly unorthodox as this type of *t*-test is used to test whether a sample mean differs significantly from a known population mean. However, it has been used in a number of studies [17-19] in this manner. The CH and Weisberg *t*-tests are more efficient in identifying significant hypomethylation than the one-sample *t*-test. For example, in TND-MLMD patients both CH and Weisberg *t*-tests were able to identify a number of sites including the cardinal disease locus *PLAGL1* in all patients. Though both the CH and Weisberg *t*-tests showed similar results for several loci, the Weisberg test generated slightly less significant *P* values (differences in *P* values ranging from 10^−9^ to 10^−15^ at the *PLAGL1* locus). The difference in *P* values is attributed to the difference in minimum *P* value threshold of CH *t*-test and Weisberg *t*-tests. Conversely, the one-sample *t*-test did identify significant sites along with many more false positives. The results of different *t*-tests examining *PLAGL1* in TND-MLMD 5 are presented in Figure 1 and Additional file 1: Table S7. The point estimates (estimated percentage of the control population that would be expected to obtain lower score than the case) for both CH and Weisberg *t*-tests are well within the 95% confidence interval of the noncentrality parameter derived from the case scores. However, in case of one-sample *t*-test, there are a number of instances in which the point estimates do not fit in to that confidence interval, showing that one-sample *t*-test predicted large numbers of insignificant hypomethylation signals as significant. Likewise, the CH and Weisberg *t*-tests produced similar results for BWS-MLMD patients, identifying several regions of differential methylation, including the cardinal disease locus *KCNQ1OT1*, whereas the one-sample *t*-test again detected many false positive sites as significant differential methylation. The results of different *t*-tests around *KCNQ1OT1* in BWS-MLMD 1 are presented in Additional file 1: Figure S1 and Additional file 1: Table S8, which shows the same type of outcome. The same samples with varying control size (5, 10, 20, 30, 40 and 50) showed the same trend in the efficiency of the *t*-tests.

Therefore, both CH and Weisberg *t*-tests are capable of identifying regions of differential methylation with low false positive rate in single sample case-controls analysis. However, the CH *t*-test has the advantage of calculating effect size for single sample case-control studies, which is absent from the Weisberg *t*-test; therefore, we selected CH *t*-test for further analysis due to this and the more significant *P* values generated, and all further tests described here were performed using this method.

### Detection of cardinal locations in MLMD patients using the single sample analysis

**Figure 2:**
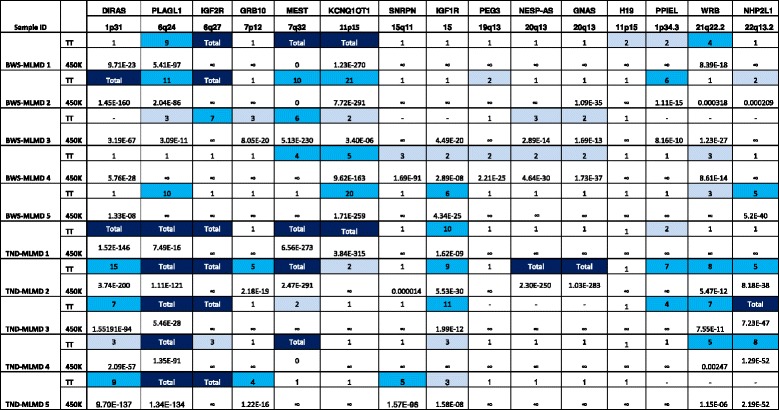
Comparison of detection of methylation changes between targeted DNA methylation testing and single sample analysis. Column headers indicate the loci tested and their genomic locations. Rows denote targeted testing (TT) and single sample analysis (450 k) results of individual patients, grouped by their presenting disorder. The DNA methylation at differentially methylated loci was estimated by methylation-specific PCR (msPCR) in TT. A methylation ratio of 1 is equivalent to hemizygous methylation, as seen in normal controls; a ratio of 2 indicates two-fold excess of unmethylated over methylated template; ‘Total’ indicates no detectable methylated sequences. The intensity of blue shading reflects the severity of hypomethylation. A dash indicates no data, normally because insufficient DNA prevented completion of all testing. For 450 k, the *P* values have been determined by Fisher’s combined *P* value method for independent tests. The ∞ symbol means no significant methylation changes were detected at that region and 0 is yielded while the *P* value is too small (<10^−350^). BWS-MLMD, Beckwith-Wiedemann syndrome-multi-locus methylation disorders; TND-MLMD, transient neonatal diabetes-multi-locus methylation disorders.

Previous targeted testing of our samples identified several known regions of biologically significant differential methylation in addition to the cardinal disease loci. However, the magnitude of differential methylation varied at different imprinted loci, for example all TND cases have complete hypomethylation at the *PLAGL1* locus, whereas individuals with BWS showed varying levels of hypomethylation at *KCNQ1OT1* (see Figure 2). These data provided us with valuable information on the inter-individual differences of both the severity of differential methylation and the regions affected.

**Figure 3:**
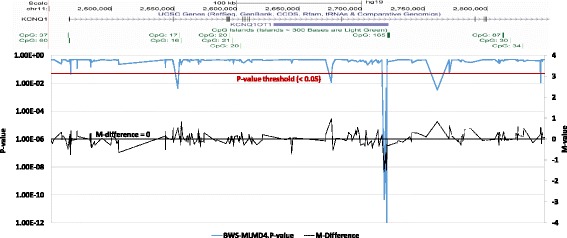
Identification of hypomethylation at the cardinal loci in an MLMD sample. Upper panel: Genomic location from the UCSC genome browser, illustrating the *KCNQ1* gene and the imprinting control region. Lower panel: graphical presentation of 450 k DNA methylation data across the *KCNQ1* gene in BWS-MLMD 4. The x-axis corresponds to the genomic location as illustrated in the upper panel. The primary y-axis (left) represents the CH *P* value (solid blue line); the secondary y-axis (right) represents the difference in *M* value between BWS-MLMD 4 and controls (dashed black line). BWS-MLMD, Beckwith-Wiedemann syndrome-multi-locus methylation disorders.

Analysis of TND-MLMD and BWS-MLMD patients’ 450 k data, using the single sample analysis pipeline and a randomly selected batch-matched control group of size 50, identified several regions of differential methylation in a number of cases. These regions are largely dependent on the magnitude of the differential methylation as predicted by targeted testing. In all patients, the cardinal disease loci were identified: that is, *PLAGL1* in TND-MLMD patients and *KCNQ1OT1* in BWS patients. Figure 3 (with Additional file 1 Table S9) illustrates the identification of hypomethylation at *KCNQ1OT1* in BWS-MLMD 4 (data for other BWS-MLMD patients are in Additional file 1: Figure S2 and for TND-MLMD cases in Additional file 1: Figure S3).

### Detection of methylation disturbance at multiple locations using single sample analysis

Apart from the cardinal loci, the pipeline also detected additional significantly hypomethylated loci, including but not limited to those identified by targeted testing. Hypomethylation was detected at well-established imprinted loci including *SNRPN*, *GNAS*, *MEST* and *GRB10*, more recently identified loci including *ZNF331*, *FAM50B*, *HM13*, *ERLIN2*, *LOC100130522*, *WRB* and *NHP2L1*, and previously uninvestigated regions (such as *SVOPL* and *MAFG*; Additional file 1: Table S1).

**Figure 4:**
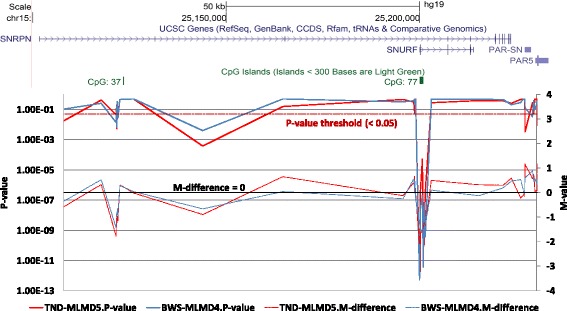
Identification of hypomethylation at the *SNRPN* locus in MLMD samples. Upper panel: Genomic location from the UCSC genome browser, illustrating the *SNRPN* gene and the imprinting control region. Lower panel: graphical presentation of 450 k DNA methylation data across the *SNRPN* gene in BWS-MLMD 4 (red) and TND-MLMD 5 (blue). The x-axis corresponds to the genomic location as illustrated in the upper panel. The primary y-axis (left) represents the CH *P* value (solid lines); the secondary y-axis (right) represents the difference in *M* value between the cases and controls (dashed lines). BWS-MLMD, Beckwith-Wiedemann syndrome-multi-locus methylation disorders; TND-MLMD, transient neonatal diabetes-multi-locus methylation disorders.

To assess the sensitivity of the pipeline, we focused on three imprinted loci: *SNRPN* (chr15: 25068738–25201732), *GNAS* (chr20:57,380,000-57,400,000) and *WRB* (chr21: 40752116–40752116). Significant hypomethylation at *SNRPN* was confirmed in TND-MLMD 5 and BWS-MLMD 4 by targeted testing (Figure 2) which is also detected by our pipeline (Figure 4, Additional file 1: Table S10). Moreover, TND-MLMD 2 was also found to show differential methylation in some *SNRPN* CpGs outside the differentially methylated region (DMR) - this was not detected by msPCR analysis (Additional file 1: Figure S4), and it shows a slightly different methylation pattern than that of TND-MLMD 5. Likewise, the mosaic hypomethylation of *WRB* detected in three TND-MLMD samples was confirmed by targeted testing methylome analysis (TND-MLMDs 2, 3, 4 and 5: Additional file 1: Figure S5).

**Figure 5:**
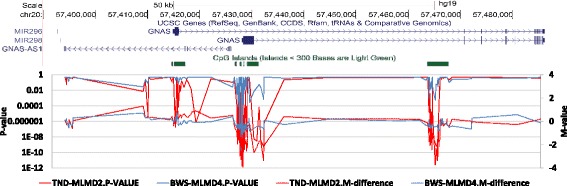
Identification of hypomethylation at the *GNAS* locus in MLMD samples. Upper panel: Genomic location from the UCSC genome browser, illustrating the *GNAS* locus and three regions of high CpG density harbouring differentially methylated regions. Lower panel: graphical presentation of 450 k DNA methylation data across the *GNAS* locus TND-MLMD 2 (red) and BWS-MLMD 4 (blue). The x-axis corresponds to the genomic location as illustrated in the upper panel. The primary y-axis (left) represents the CH *P* value (solid lines); the secondary y-axis (right) represents the difference in *M* values between cases and controls (dashed lines). Note the hypomethylation clearly visible at three locations in TND-MLMD 2, coinciding with the more subtle hypomethylation detectable in BWS-MLMD 4 primarily through significance of *P* value. BWS-MLMD, Beckwith-Wiedemann syndrome-multi-locus methylation disorders; TND-MLMD, transient neonatal diabetes-multi-locus methylation disorders.

The pipeline detected significant methylation changes across the *GNAS* locus in three out of five BWS-MLMD and one out of five TND-MLMD patients. Figure 5 (with Additional file 1: Table S11) illustrates the *GNAS* locus in BWS-MLMD 4 and TND-MLMD 2, showing that methylation changes are detected at consecutive probes across the locus, which is more informative than the point determinations of targeted testing.

### Use of the single sample approach on ‘simple’ patients

In addition to the samples with MLMD, we analysed samples from patients with ‘simple’ imprinting disorders where targeted testing indicated that hypomethylation was restricted to the cardinal disease locus. In all four TND samples, the cardinal region of differential methylation was *PLAGL1* as expected. For two samples, TND-SIMPLE 2 and 3, hypomethylation of five and one additional imprinted regions was identified respectively, characteristic of MLMDs (Additional file 1: Table S2). Additionally, one of the samples (TND-SIMPLE 2) had many novel regions of hypomethylation not previously associated with imprinted loci. One of these, *GLP2R* was also observed in TND-SIMPLE 3 as the only hypomethylated locus not associated with a known imprinting region. Likewise, all the BWS samples were hypomethylated at the cardinal locus *KCNQ1OT1*, but one sample (BWS-SIMPLE3) showed hypomethylation at multiple imprinting loci, characteristic of MLMD. This shows that 450 k-based analyses can detect methylation changes that may go undetected by the point determinations of targeted testing. (Additional file 1: Table S3).

### Applying the single sample analysis to biological replicates

To assess whether the CH *t*-test detected false positives from control group variations, we processed one sample with two completely different batch-matched groups of 50 controls. The two tests respectively selected 205 and 184 hypomethylated CpG sites, with 170 sites in 12 regions in common (see Additional file 1: Table S4).

### Determining the minimum number of controls

#### Significance test

To assess the effect of control group size on detection of known regions of differential methylation, BWS-MLMD samples were analysed with varying numbers of controls (5, 10, 20, 30, 40 or 50) using the CH *t*-test. With 5 controls, no cardinal sites for BWS-MLMDs were detected. When control group size = 10, numerous regions of hypomethylation were identified (*KCNQ1*, *PLAGL1*, *DIRAS3*, *MEST*, *GNAS*, *PEG3*, *NHP2L1*, and *PPIEL*) though not *WRB*. With 20 controls all known regions of differential methylation were detected, the use of 30, 40 or 50 controls added little sensitivity. Similar results were obtained for TND-MLMD cases. In summary, 10 controls can produce statistically and biologically significant results, though 20 controls are preferable to obtain higher sensitivity. Additional file 1: Table S5 presents the sites found in TND-MLMD and BWS-MLMD samples using the varying control sizes using the CH *t*-test. No further significant improvement was observed using a larger control group (>50 controls), therefore, we restricted our maximum number of controls to 50.

#### Effect size calculation

In order to determine the effect of the CH *t*-test on the magnitude of differential methylation, the effect size for each sample was calculated against variable numbers of controls (5, 10, 20, 30, 40 or 50). For both TND-MLMD and BWS-MLMD samples, at the majority of differentially methylated regions, the effect sizes were similar irrespective of the number of controls. However, at some regions of differential methylation, the effect size was greater when control group size = 5 rather than ≥10. To determine the reliability of the effect size, point estimates and 95% confidence intervals of those effect sizes were calculated.

In general for TND-MLMD samples, the confidence interval was strikingly wider with 5 than 10 controls. For example, at the *PLAGL1* region in TND-MLMD3, with 5 controls the effect size was −24.650, but the confidence interval was wide (−41.162 to −8.549), indicating this effect size as unreliable. With 40 controls, the effect size was much smaller (−13.875), but its confidence interval (−16.953 to −10.789) was tighter (Additional file 1: Table S6). With 20 controls, the effect size was intermediate −21.309, with confidence interval −28.033 to −14.574. The width of the confidence interval is attributed to the extreme hypomethylation of the *PLAGL1* locus, which is a typical biological finding in TND but detrimental to effect size. For subtle changes in methylation, the effect sizes were much smaller, and use of 10, 20 or 30 controls resulted in large effect sizes with tight confidence intervals in both TND-MLMD and BWS-MLMD. Therefore, 20 controls appeared optimal for single sample analyses.

## Discussion

There are a number of motivations for developing this single sample case-control method for analysing Illumina 450 k methylation data. Firstly, the study population - patients with imprinting disorders - is small, and classic case-control studies would not yield statistically significant results. Secondly, individual patients have unique clinical features and unique epimutations and therefore require individual analysis to yield relevant epigenetic data with clinical utility. Thirdly, our former small-sample analysis approach [7] requires large control numbers to attain high statistical robustness, which is not always feasible for analysing single patients. Fourthly, use of large control batches would be prohibitively expensive if epigenomic array analysis is to be adopted as a pragmatic tool for epigenetic diagnosis of patients. Technical replication of the same sample in different batches, with different controls, clearly confirmed that our approach robustly detected statistically significant sites. The method also identified outlier samples, which is not possible for grouped case-control studies. The pipeline clearly identified one BWS-HIL patient with large abnormal DNA methylation variations (may be due to technical variations) in multiple locations, though these may be due to technical variation (Additional file 1: Table S1). It should be noted that the threshold *P* value of 0.05 is not as stringent as that used for case-control analyses (< 1.33 × 10^− 7^) but nonetheless does robustly identify imprinted loci.

The CH *t*-test method has the advantage of reporting not only probability of significant methylation changes but also the magnitude of the change by its effect size point estimate and confidence intervals. The power calculation interval shows the uncertainty of the point estimate of the effect size and its variation with the number of controls [20]. Using this metric gave a concrete indication of the number of controls required to yield significant results.

The optimal number of controls for this approach was determined empirically, as the number of controls for which known imprinted loci were robustly detected. In broad terms, fewer than ten controls gave unreliable effect sizes (large confidence intervals), whereas control sizes of 10 and 20 gave improvements in confidence, and a modest additional improvement was achieved for >20 controls. We, therefore, suggest that 20 controls in the same batch are optimal for this approach, and using 10 controls is feasible in statistical terms. However, a requirement for large numbers of controls is not ideal for use in a diagnostic setting where cost is a consideration. We are currently attempting to identify robust methods for identifying methylation changes without the need for batch-matched controls. Though it is true that use of smaller numbers of controls runs the risk of violating the normality assumption, the effect of departure from normality is modest in case of the CH-test, as it is capable of controlling the Type I error rate [21,22]. While using large numbers of controls assures the normality of the distribution from the controls, in our empirical tests, we observed only incremental increases in statistical power with increase in control number above 20 controls.

We found the 450 k array to have unexpected benefits compared with targeted testing. Firstly, 450 k analysis is by definition an epigenome-wide approach and therefore detected DNA methylation variation at other loci not normally assessed in targeted testing for imprinting disorders. This expands of the scope of differentially methylated regions for future analysis. Secondly, 450 k data analysis was sensitive to subtle methylation changes at differentially methylated regions to the point where it detected variations that were undetected in targeted testing. Two cases that appeared by targeted testing to show ‘simple’ methylation changes (one TND and one BWS) were shown by 450 k array to have MLMD with subtle variations at several imprinted loci, which may be relevant to the clinical presentation of these individuals. This sensitivity probably stems from the fact that differentially methylated regions of imprinted genes frequently span tens or hundreds of CpG dinucleotides. While targeted testing is a single-point analysis, so a subtle variation may not be distinguishable from the normal range, whereas using the 450 k array a subtle variation may be reiterated many times sequentially, increasing its statistical robustness. Thirdly, differentially methylated regions are typified by dense clustering of CpG dinucleotides, and 450 k analysis reports on multiple CpGs in any given locus, and therefore, it gives information about the extent of methylation anomalies across a locus. This may offer novel information about the extent and effects of methylation changes across gene clusters.

An obvious limitation of 450 k-based analysis is that the array targets only a small percentage of potentially methylated cytosines in the genome; therefore, additional loci affected in these patients may remain undetected by this method. However, the disadvantage of incomplete coverage is offset by the advantages of cost and technical consistency.

450 k array-based analysis has not previously been used on patients with imprinting disorders, because their rarity and heterogeneity precluded the use of established case-control cohort studies. This is potentially very important for imprinting disorders, where standard diagnostic testing is fragmented, time-consuming and variably sensitive, and where clinically heterogeneous and overlapping features (for example pre- and post-natal growth dysregulation) can be associated with multiple epigenetic mutations, many of which are not included in current testing regimes. 450 k analysis offers potential for diagnosis of known imprinting disorders and for detection of novel patterns of methylation anomalies. This may lead to substantial improvements in the diagnostic rate and translational research for imprinting disorders, in the same way that genome-wide array analysis has advanced the clinical genetics of common diseases over the last fifteen years [23-25]. Intriguingly, methylation variation may also act as a biomarker of underlying genetic anomalies. It is well known that some deleterious genetic/genomic variations can be detected by means of consequent methylation changes: for example, FRAX triplet-repeat expansions cause promoter methylation and inactivation of the FRAX gene and Fragile X mental retardation [26], deletions and rearrangements of the *IGF2* enhancer attenuate *IGF2* expression with co-ordinate hypomethylation of promoter sequences [27] and genetic rearrangements in Lynch syndrome are detectable as epigenetic inactivation of *MSH2* [28]. We suggest that epigenome-wide DNA methylation analysis may be a powerful adjunct to genomic analysis, since it may indirectly indicate genomic variations that do not alter coding sequence but do alter gene expression.

## Conclusions

Using the Crawford-Howell *t*-test in single sample case-control studies is a novel approach for analysing Illumina 450 k array methylation data. By this method, we identified statistically and biologically significant hypomethylation in individuals at both known and novel sites. We suggest that single sample analysis makes possible the use of the 450 k array as a translational research or diagnostic tool for human disorders associated with disturbance of DNA methylation.

## Methods

### Study and control populations

For this study, we selected patients with two imprinting disorders, Transient Neonatal Diabetes (TND) and Beckwith-Wiedemann Syndrome (BWS). These patients have been described previously, and their methylation levels determined at several imprinted loci by targeted testing [7,9,29]. In our recent study [7], five multi-locus methylation disorder patient samples from each clinically classified group (TND or BWS) were processed in separate batches with 245 and 221 anonymous healthy controls, respectively, from an unrelated study. In this study, we additionally included four TND and three BWS patients where targeted testing detected DNA hypomethylation only at the cardinal disease loci, with no known involvement of any other imprinted locus (denoted ‘simple’ BWS and TND cases). These samples were processed in a third batch with 63 anonymous healthy controls from an unrelated study. Batch-matched controls were chosen as the control group and randomly selected for each sample analysed in the single sample analysis pipeline.

### Data analysis

To assess the methylation level in each sample, a standard workflow was followed. The DNA in each sample was extracted from the whole blood by the standard procedure described in [30], and DNA concentration was determined using PicoGreen dsDNA Quantitation Kit (Molecular Probes, Inc., OR, USA). One microgram of DNA was bisulfite-treated for converting cytosine to thymine using the EZ 96-DNA Methylation Kit (Zymo Research, CA, USA). Illumina Infinium HumanMethylation450 BeadChip (Illumina, Inc., CA, USA), which was processed following standard protocol [31], was used to estimate genome-wide DNA methylation. Multiple identical control samples were assigned to each batch to assess assay variability and control batch effects. The BeadChips were scanned by the BeadStation and the methylation levels, as beta (β) values, were extracted using the Methylation Module of GenomeStudio (version 2011.1). The methylation data were then pre-processed further, as described in the following section.

### Single sample analysis pipeline

**Figure 6:**
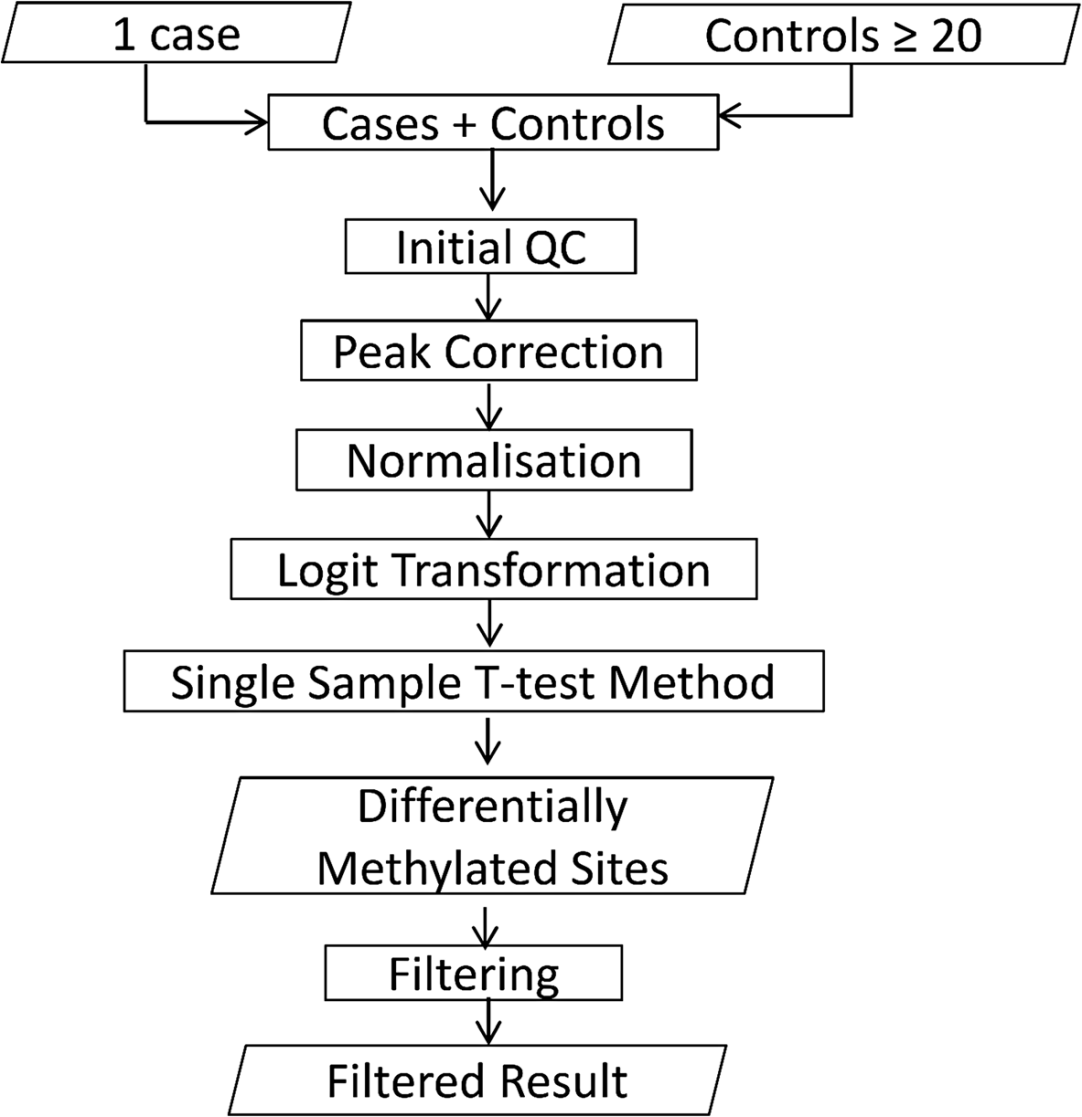
Workflow of the single case-control pipeline. Each single case was pre-processed with the controls using IMA package, and then the Crawford-Howell *t*-test method was implemented to identify differentially methylated sites. To reduce the rate of false positives, filtration criteria were set to obtain filtered results.

The single sample analysis pipeline was developed combining the Illumina Methylation Analyzer (IMA) package [32] and implementation of single sample *t*-tests within the R statistical analysis environment (http://www.r-project.org). In the first stage, the IMA package is used for pre-processing and quality control, and the output data are used single sample analysis. The workflow of the pipeline is shown in Figure 6, and the steps are described as follows.

#### Pre-processing

Pre-processing of the 450 k data first removes any CpG sites with missing values, followed by removal of any sample where >90% CpG sites have detection *P* value >0.05, and any CpG sites where >75% samples have detection *P* value >10^−5^. Probes on the X and Y chromosomes were removed to discard any sex bias within the samples. The beta-values were converted to logit transformed *M* values, and quantile normalisation was used to normalise signal intensities to reduce inter-array variation [33]. Peak correction [34] was applied to correct differences between Infinium I and Infinium II type assays. No batch correction was required as each case and its corresponding controls were drawn from the same batch. Statistically significant differences between the pre-processed *M* values of cases and controls were determined using single sample *t*-tests.

#### Statistical tests for identifying significant differential methylation

In our single sample studies, we mainly used the CH *t*-test method (described in [12] and [35]) for statistical analysis of the pre-processed data. The reasons for this selection are presented in the Results and Discussion sections. This method is an alternative *t*-test method, which treats control sample statistics as statistics rather than as a population parameter. The CH *t*-test is described by

$$ {t}_{CH}=\frac{x^{*}-\overline{x}}{s\;\sqrt{\frac{n+1}{n}}} $$

Where x^*^ is the single sample score, $\overline{x}$ and *s* are the mean and standard deviation of scores in control samples, respectively, and *n* is the size of the control sample. If the *t*-value (t_CH_) falls below the one-tailed 5% critical value for *t* on n-1 degrees of freedom (df), then it can be said that the case score sufficiently differs from the control population to refute the null hypothesis. For an example, a control sample of 10 samples (*n* = 10) has a mean of 0.5 ($\overline{x}=0.5$) and standard deviation of 0.1 (*s* = 0.1). If the case score is 0.4 (x^*^ = 0.4), the *t*-value, from CH-test, is 0.954 with 9 df and a one-tailed probability using Student *t*-distribution of 0.365. Therefore, the case score is not low enough to reject the null hypothesis that the case score is drawn from the control population.

To establish the optimal test for or single sample analysis, we compared the CH *t*-test method to other two *t*-tests, namely - one-sample and Weisberg *t*-tests.

The one-sample *t*-test draws inferences regarding significant differences between a single case and control scores. It compares the known control sample mean with the score of a single case, which is hypothesised as a population mean. The formula for the one-sample *t*-test is

$$ {t}_{OS}=\frac{\overline{x}-{x}^{*}}{s/\sqrt{n}} $$

However, the one-sample *t*-test exhibits a high type I error. For an example, if we use the same measures as above ($n=10;\;\overline{x}=0.5;s=0.1;{x}^{*}=0.4$), we obtain a *t*-value of 3.162 with 9 df and the one-tailed probability is 0.012, which incorrectly rejects the null hypothesis.

On the other hand, the Weisberg *t*-test for outliers (described in [36]) also can detect abnormal scores of a single sample against a limited number of control samples. The formula for the Weisberg *t*-test is

$$ {t}_{WB}=\frac{x^{*}-\overline{x}}{s\;\sqrt{\frac{n}{n-1}}} $$

If we apply the same example in case of Weisberg *t*-test, we obtain a *t*-value of −0.949 with 8 df, and the one-tailed probability is 0.371.

#### Power calculations for single sample analysis

In order to determine the magnitude of loss of methylation at significant sites/regions for single sample case-control analysis, application of a significance test alone is not ideal [37]. Therefore, we applied a power calculation for the CH *t*-test to generate an effect size estimate using the *P* value from [20]. This is similar to Cohen’s *d*, which is the difference between the means of case and control samples in standardised units divided by the pooled standard deviation of the two samples [20]. Similarly for the CH *t*-test, the effect size index is calculated using the difference between the single case score (*x*) and the mean of controls ($\overline{x}$) divided by the standard deviation of controls (*s*_*x*_):

$$ {z}_{cc}=\frac{x-\overline{x}}{s_x} $$

For an example, a control group of 10 samples (*n* = 10) has a mean of 0.5 ($\overline{x}=0.5$) and standard deviation of 0.1 (*s* = 0.1). If the case score is 0.4 (x^*^ = 0.4), *z*_cc_ = −1.0, and ${z}_{cc}\sqrt{n}\kern0.5em =\kern0.5em -3.162$. The noncentrality parameter for the *t*-distribution having −3.162 as its 0.975 percentile point with 9 df is −5.538. Therefore, the lower limit is $-5.538/\sqrt{n}\kern0.5em =\kern0.5em -1.751$. In the same way, the upper limit of *z*_cc_ can be calculated as −0.214.

The *t*-value from the CH *t*-test shows the statistical significance of the difference between case and controls, whereas the effect size index shows the level of difference between them. Along with the point estimate of the effect size, an estimate interval should also be presented in the single sample analysis. The procedure used in this paper to measure the confidence interval of the point estimate of the effect size has been described previously in [35], and the calculation is further explained in [20].

#### Filtering criteria

To reduce false positive calls, we further filtered the results from the significant difference between case and controls groups by CH *t*-test and power calculation. To define sites that were hypomethylated in cases, we initially set the same stringent criteria as in [7]: one-tailed *P* value (adjusted using false discovery rate) < 10^−7^ and *M* value between −1 and +1 in normal controls with the beta-differences smaller than zero (to select only hypomethylated loci). Genes containing at least three CpGs meeting these criteria within <2000 bp (base pair) were selected as candidate DMRs consistent with imprinting. However, when applied to single sample analysis, these criteria were too stringent to detect known differentially methylated regions. Therefore, for single sample analyses, we used a less stringent *P* value, which was calculated as described in *Statistical tests for identifying significant differential methylation*: significant methylation changes were therefore selected as those containing a minimum of three consecutive CpGs within 2000 nucleotides with *M* values between −1 and +1 in normal controls and *P* value <0.05.

#### Minimum number of controls

Using varying numbers of controls (5, 10, 20, 30, 40 or 50) we assessed the impact of control group size in detecting known regions of hypomethylation and changes in effect size and confidence interval in our single sample analysis.

## Additional file

Additional file 1:
**Supplementary figures and tables.**

## References

[1] Feinberg AP (2007). Phenotypic plasticity and the epigenetics of human disease. Nature.

[2] Yang BZ, Zhang H, Ge W, Weder N, Douglas-Palumberi H, Perepletchikova F (2013). Child abuse and epigenetic mechanisms of disease risk. Am J Prev Med.

[3] Shenker NS, Polidoro S, van Veldhoven K, Sacerdote C, Ricceri F, Birrell MA (2013). Epigenome-wide association study in the European Prospective Investigation into Cancer and Nutrition (EPIC-Turin) identifies novel genetic loci associated with smoking. Hum Mol Genet.

[4] Moore K, McKnight AJ, Craig D, O’Neill F (2014). Epigenome-wide association study for Parkinson’s disease. Neuromolecular Med.

[5] Seow WJ, Kile ML, Baccarelli AA, Pan WC, Byun HM, Mostofa G (2014). Epigenome-wide DNA methylation changes with development of arsenic-induced skin lesions in Bangladesh: a case-control follow-up study. Environ Mol Mutagen.

[6] Abdolmaleky HM, Nohesara S, Ghadirivasfi M, Lambert AW, Ahmadkhaniha H, Ozturk S (2014). DNA hypermethylation of serotonin transporter gene promoter in drug naive patients with schizophrenia. Schizophr Res.

[7] Docherty LE, Rezwan FI, Poole RL, Jagoe H, Lake H, Lockett GA (2014). Genome-wide DNA methylation analysis of patients with imprinting disorders identifies differentially methylated regions associated with novel candidate imprinted genes. J Med Genet.

[8] Mackay DJ, Callaway JL, Marks SM, White HE, Acerini CL, Boonen SE (2008). Hypomethylation of multiple imprinted loci in individuals with transient neonatal diabetes is associated with mutations in ZFP57. Nat Genet.

[9] Bliek J, Verde G, Callaway J, Maas SM, De Crescenzo A, Sparago A (2009). Hypomethylation at multiple maternally methylated imprinted regions including PLAGL1 and GNAS loci in Beckwith-Wiedemann syndrome. Eur J Hum Genet.

[10] Azzi S, Rossignol S, Steunou V, Sas T, Thibaud N, Danton F (2009). Multilocus methylation analysis in a large cohort of 11p15-related foetal growth disorders (Russell Silver and Beckwith Wiedemann syndromes) reveals simultaneous loss of methylation at paternal and maternal imprinted loci. Hum Mol Genet.

[11] Eggermann T (2010). Russell-Silver syndrome Imprinted genes and human disease. Am J Med Genet Part C Semin Med Genet.

[12] Crawford JR, Howell DC (1998). Comparing an individual’s test score against norms derived from small samples. Clin Neuropsychol.

[13] Crawford JR, Laura H, Goldstein JE (2004). Psychometric foundations of neuropsychological assessment. Clinical Neuropsychology: a practical guide to assessment and management for clinicians.

[14] Howell DC (2002). Statistical methods for psychology.

[15] Crawford JR, Garthwaite PH (2007). Comparison of a single case to a control or normative sample in neuropsychology: development of a Bayesian approach. Cogn Neuropsychol.

[16] Crawford JR, Garthwaite PH (2012). Single-case research in neuropsychology: a comparison of five forms of *t*-test for comparing a case to controls. Cortex.

[17] Reinhold N, Markowitsch HJ (2007). Emotion and consciousness in adolescent psychogenic amnesia. J Neuropsychol.

[18] Vecera SP, Rizzo M (2004). What are you looking at? Impaired ‘social attention’ following frontal-lobe damage. Neuropsychologia.

[19] Brand M, Kalbe E, Kracht LW, Riebel U, Munch J, Kessler J (2004). Organic and psychogenic factors leading to executive dysfunctions in a patient suffering from surgery of a colloid cyst of the Foramen of Monro. Neurocase.

[20] Crawford JR, Garthwaite PH, Porter S (2010). Point and interval estimates of effect sizes for the case-controls design in neuropsychology: rationale, methods, implementations, and proposed reporting standards. Cogn Neuropsychol.

[21] Crawford JR, Garthwaite PH (2005). Testing for suspected impairments and dissociations in single-case studies in neuropsychology: evaluation of alternatives using monte carlo simulations and revised tests for dissociations. Neuropsychology.

[22] Crawford JR, Garthwaite PH, Azzalini A, Howell DC, Laws KR (2006). Testing for a deficit in single-case studies: effects of departures from normality. Neuropsychologia.

[23] de Vries BB, Pfundt R, Leisink M, Koolen DA, Vissers LE, Janssen IM (2005). Diagnostic genome profiling in mental retardation. Am J Hum Genet.

[24] Menten B, Maas N, Thienpont B, Buysse K, Vandesompele J, Melotte C (2006). Emerging patterns of cryptic chromosomal imbalance in patients with idiopathic mental retardation and multiple congenital anomalies: a new series of 140 patients and review of published reports. J Med Genet.

[25] Stankiewicz P, Beaudet AL (2007). Use of array CGH in the evaluation of dysmorphology, malformations, developmental delay, and idiopathic mental retardation. Curr Opin Genet Dev.

[26] Gerhardt J, Zaninovic N, Zhan Q, Madireddy A, Nolin SL, Ersalesi N (2014). Cis-acting DNA sequence at a replication origin promotes repeat expansion to fragile X full mutation. J Cell Biol.

[27] Gronskov K, Poole RL, Hahnemann JM, Thomson J, Tumer Z, Brondum-Nielsen K (2011). Deletions and rearrangements of the H19/IGF2 enhancer region in patients with Silver-Russell syndrome and growth retardation. J Med Genet.

[28] Ligtenberg MJ, Kuiper RP, Chan TL, Goossens M, Hebeda KM, Voorendt M (2009). Heritable somatic methylation and inactivation of MSH2 in families with Lynch syndrome due to deletion of the 3′ exons of TACSTD1. Nat Genet.

[29] Mackay DJ, Boonen SE, Clayton-Smith J, Goodship J, Hahnemann JM, Kant SG (2006). A maternal hypomethylation syndrome presenting as transient neonatal diabetes mellitus. Hum Genet.

[30] Miller SA, Dykes DD, Polesky HF (1988). A simple salting out procedure for extracting DNA from human nucleated cells. Nucleic Acids Res.

[31] Bibikova M, Fan JB (2009). GoldenGate assay for DNA methylation profiling. Methods Mol Biol.

[32] Wang D, Yan L, Hu Q, Sucheston LE, Higgins MJ, Ambrosone CB (2012). IMA: an R package for high-throughput analysis of Illumina’s 450 K Infinium methylation data. Bioinformatics.

[33] Dempster EL, Pidsley R, Schalkwyk LC, Owens S, Georgiades A, Kane F (2011). Disease-associated epigenetic changes in monozygotic twins discordant for schizophrenia and bipolar disorder. Hum Mol Genet.

[34] Dedeurwaerder S, Defrance M, Calonne E, Denis H, Sotiriou C, Fuks F (2011). Evaluation of the Infinium Methylation 450 K technology. Epigenomics.

[35] Crawford JR, Garthwaite PH (2002). Investigation of the single case in neuropsychology: confidence limits on the abnormality of test scores and test score differences. Neuropsychologia.

[36] Weisberg S (1985). Probability and mathematical statistics: applied linear regression.

[37] Sullivan GM, Feinn R (2012). Using effect size-or why the p value is not enough. J Grad Med Educ.

