# Supplementary material for: A statistical method for single sample analysis of HumanMethylation450 array data: genome-wide methylation analysis of patients with imprinting disorders
Source: Clin Epigenetics. 2015 Apr 21;7(1):48. doi: 10.1186/s13148-015-0081-5 (PMC4410592; doi:10.1186/s13148-015-0081-5)
Supplement: Additional file 1: — Supplementary figures and tables. [file 13148_2015_81_MOESM1_ESM.pdf]

Supplementary Figures and Tables

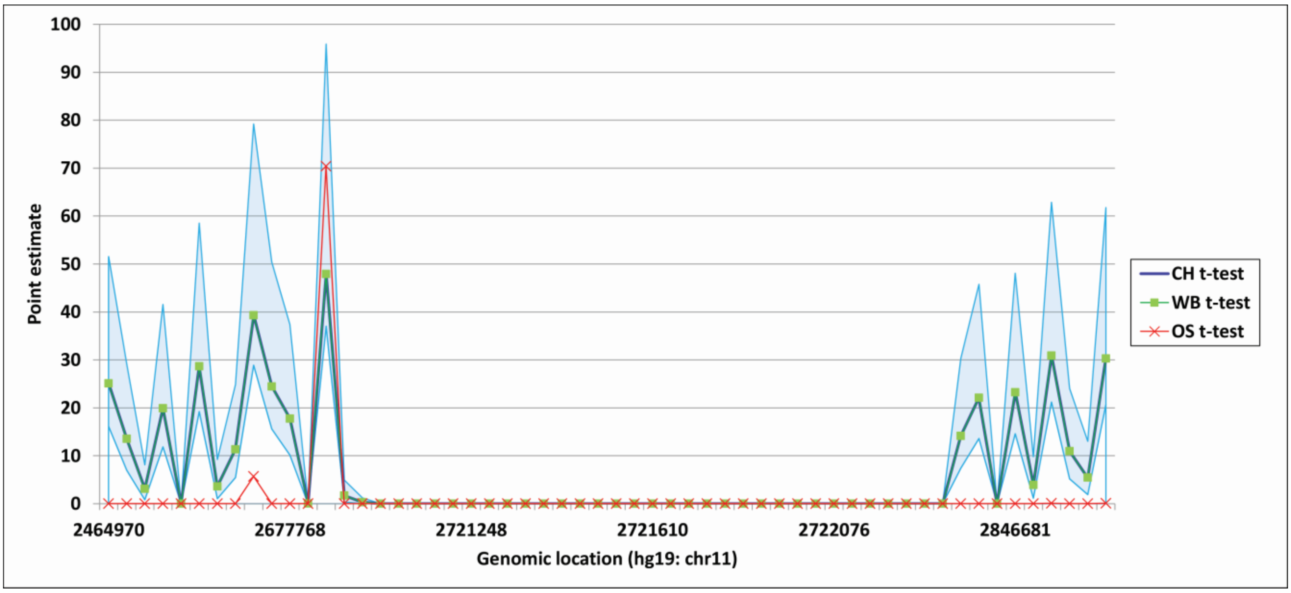

Supplementary Figure 1. The performance of three t-tests (one-sample, Weisberg, Crawford-Howell t-tests) around the *KCNQ1OT1* region in BWS-MLMD3. The x-axis denotes the genomic location of *KCNQ1OT1* on chromosome 11. The y-axis represents the estimated percentage of the control population that would be expected to obtain lower score than the case (point estimate), which is calculated according to the one-sample (OS, red crossed line), Weisberg (WB, green line with green square markers) and Crawford-Howell (CH, blue line) methods. The blue shade represents 95% confidence interval point estimates from the noncentrality parameter from a noncentral t-distribution.

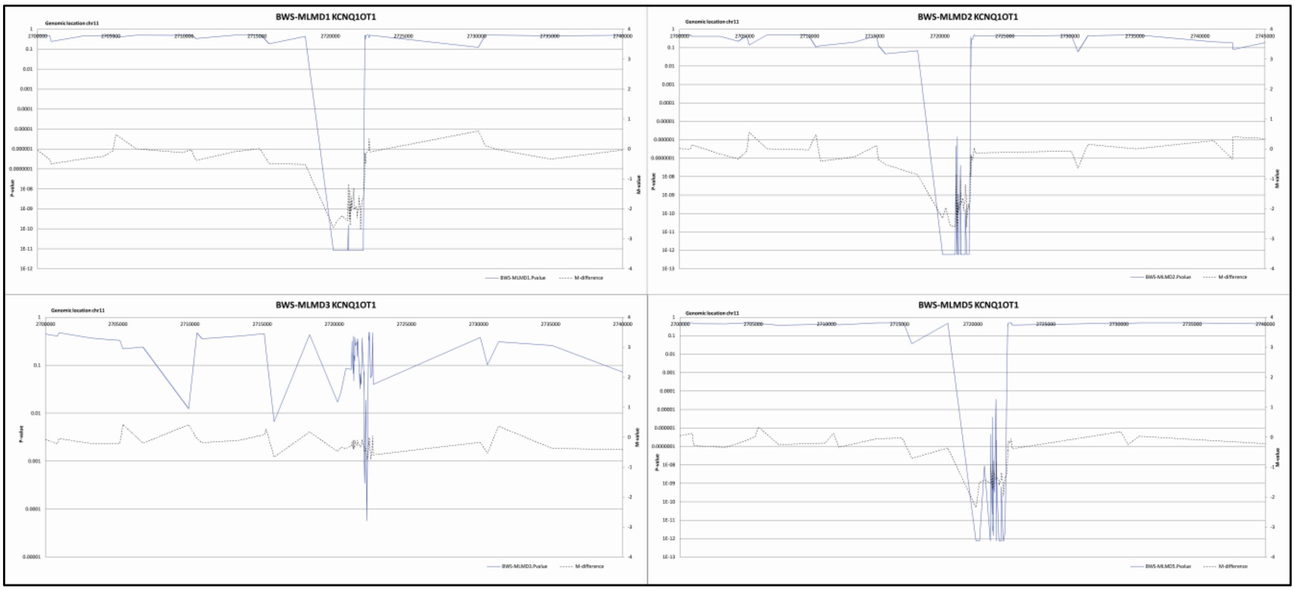

Supplementary Figure 2. Identification of hypomethylation at the cardinal loci (KCNQ1OT1) in four BWS-MLMD samples. Graphical presentation of 450k DNA methylation data across the KCNQ1OT1 gene in BWS-MLMDs1, 2, 3, and 5. The x-axis corresponds to the genomic location at chr11 (hg19). The primary y-axis (left) represents the CH P-value (solid blue line); the secondary y-axis (right) represents the difference in M value between BWS-MLMD cases and controls (dashed black line).

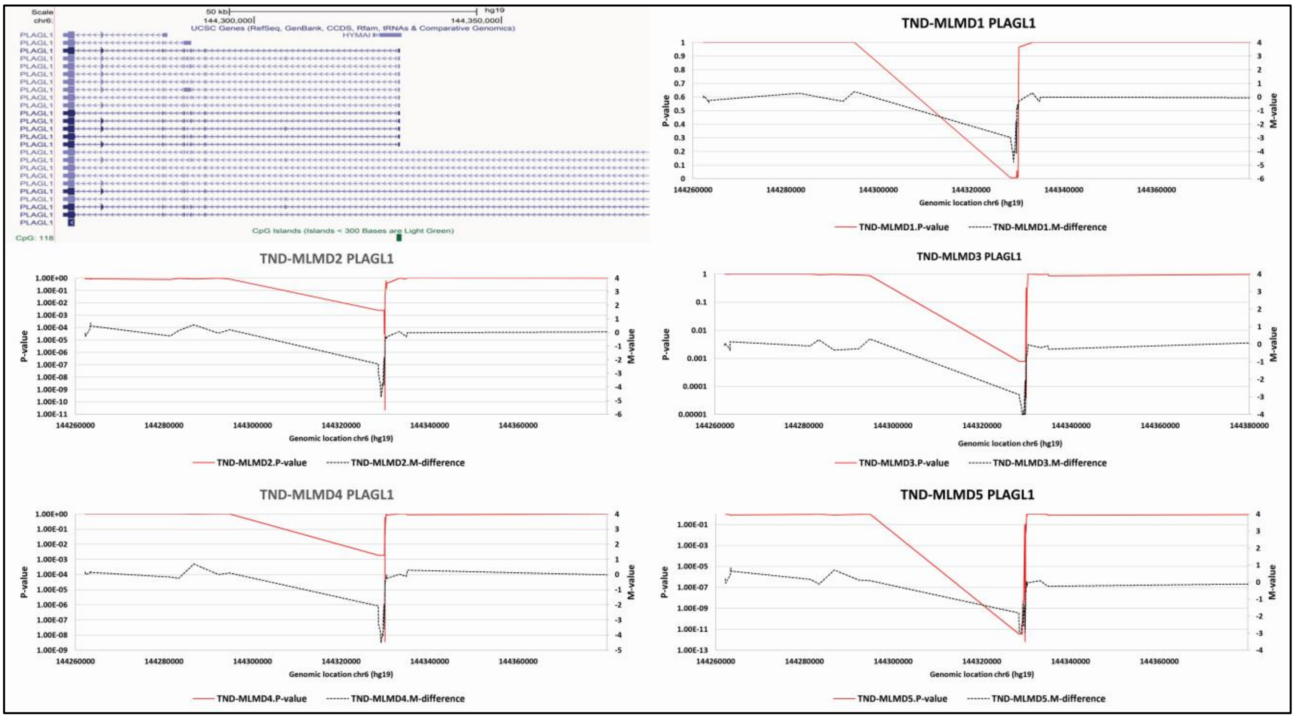

Supplementary Figure 3. Identification of hypomethylation at the cardinal loci (PLAGL1) in five TND-MLMD samples. Upper leftmost figure: Genomic location from the UCSC genome browser, illustrating the PLAGL1 gene and the imprinting control region. Five other figures: graphical presentation of 450k DNA methylation data across the PLAGL1 gene in TND-MLMDs 1-5. The x-axis corresponds to the genomic location as illustrated in the upper panel. The primary y-axis (left) represents the CH P-value (solid red line); the secondary y-axis (right) represents the difference in M value between TND-MLMD cases and controls (dashed black line).

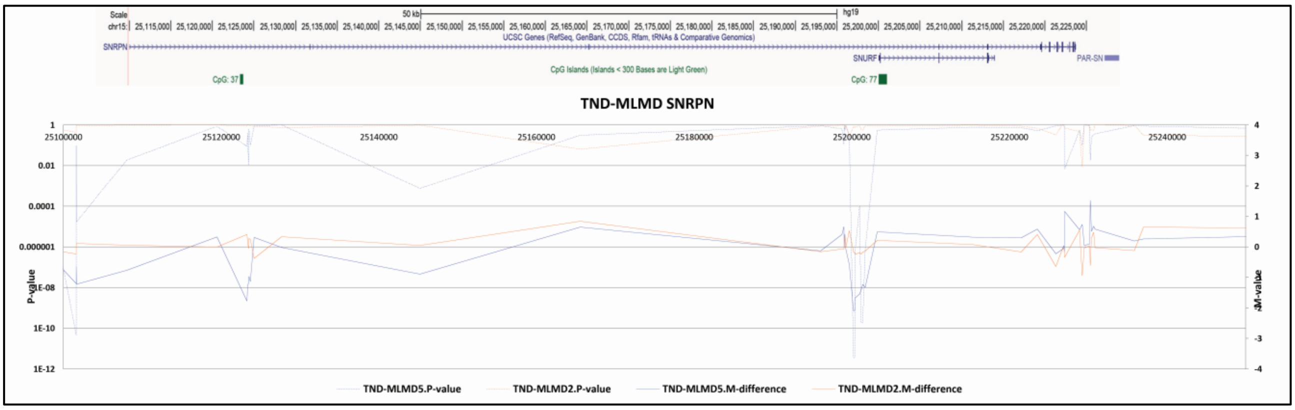

Supplementary Figure 4. Identification of hypomethylation at additional loci (SNRPN) in TND-MLMD2 and TND-MLMD5. Upper panel: Genomic location from the UCSC genome browser, illustrating the SNRPN gene and the imprinting control region. Lower panel: graphical presentation of 450k DNA methylation data across the SNRPN gene in TND-MLMD2 (red) and TND-MLMD5 (blue). The x-axis corresponds to the genomic location as illustrated in the upper panel. The primary y-axis (left) represents the CH P-value (solid lines); the secondary y-axis (right) represents the difference in M value between the cases and controls (dashed lines).

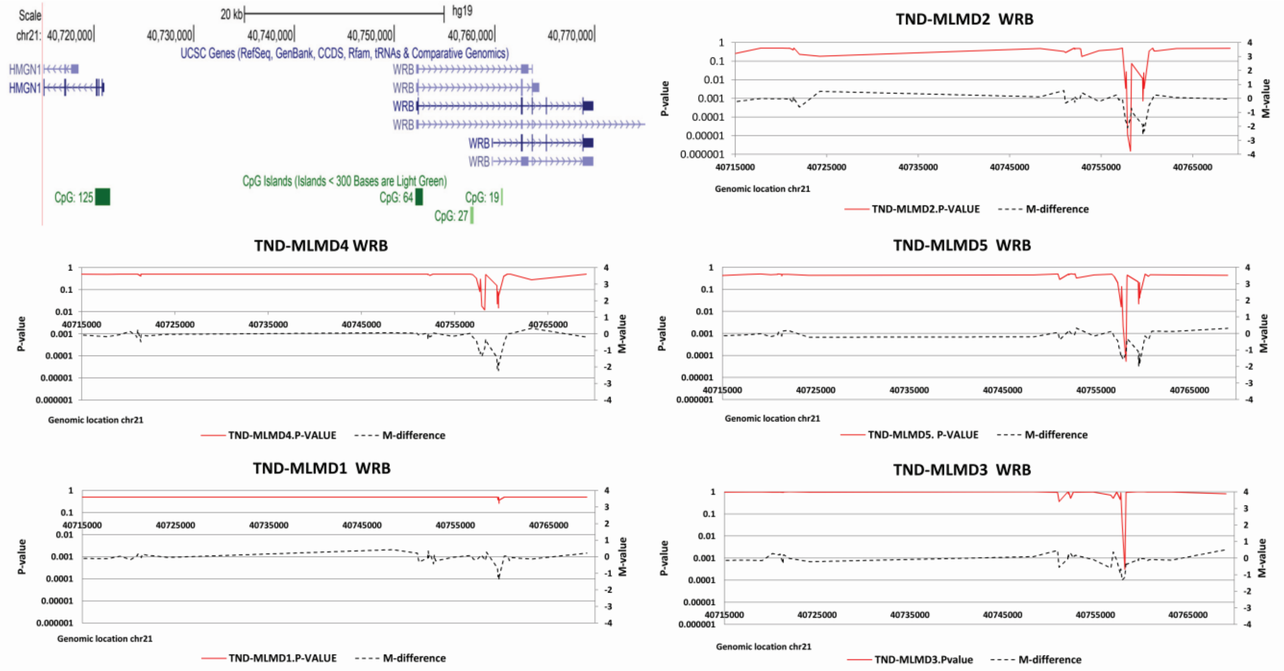

Supplementary Figure 5. Identification of hypomethylation at WRB region in five TND-MLMD samples. Upper leftmost figure: Genomic location from the UCSC genome browser, illustrating the WRB gene and the imprinting control region. Five other figures: graphical presentation of 450k DNA methylation data across the WRB gene in TND-MLMDs 1-5. The x-axis corresponds to the genomic location as illustrated in the upper panel. The primary y-axis (left) represents the CH P-value (solid red line); the secondary y-axis (right) represents the difference in M value between TND-MLMD cases and controls (dashed black line).

Supplementary Table 1. Genomic regions shared between TND-MLMD and BWS-MLMD cases, as identified by the single sample analysis pipeline. Tables 1(a) and 1(b) represent the results for the five TND-MLMD and five BWS-MLMD cases against 50 controls respectively. Probes with M-values between −1 and +1 in controls and relative hypomethylation in patients with a p-values of <0.05 were identified. This subset was further filtered by minimal criteria for a hypomethylated locus, that is, a minimum 3 consecutive hypomethylated probes spaced by <2000 nucleotides. Candidate regions that meet these criteria in both TND-MLMD and BWS-MLMD cases are listed in the tables.

(1a) TND-MLMDs

TND-MLMD1	TND-MLMD2	TND-MLMD3	TND-MLMD4	TND-MLMD5
DIRAS3	*DIRAS3*	*DIRAS3*	*DIRAS3*	*DIRAS3*
HYMAI;PLAGL1	*HYMAI;PLAGL1*	*HYMAI;PLAGL1*	*HYMAI;PLAGL1*	*HYMAI;PLAGL1*
JAKMIP1	*JAKMIP1*		*JAKMIP1*	*JAKMIP1*
ERLIN2;LOC728024	*ERLIN2;LOC728024*			*ERLIN2;LOC728024*
LOC100130522	*LOC100130522*	*LOC100130522*	*LOC100130522*	*LOC100130522*
IGF1R	*IGF1R*	*IGF1R*		*IGF1R*
	WRB	*WRB*	*WRB*	*WRB*
FAM50B	*FAM50B*	*FAM50B*		*FAM50B*
KCNQ1;KCNQ1OT1				
MEST;MESTIT1	*MEST;MESTIT1*		*MEST;MESTIT1*	
ZNF331	*ZNF331*			*ZNF331*
	NHP2L1	*NHP2L1*	*NHP2L1*	*NHP2L1*
L3MBTL	*L3MBTL*	*L3MBTL*		
	HM13;PSIMCT-1		*HM13;PSIMCT-1*	
	GNAS;GNASAS			
	GLP2R			*GLP2R*
	GRB10			*GRB10*
		SVOPL		
	PEG10;SGCE	*PEG10;SGCE*		
	NPAS3			*NPAS3*
	SNRPN;SNURF			*SNRPN;SNURF*
	RB1			*RB1*
				MAGEL2
				SEPT8
	SEPT9			
	PEG10			
	RBMXL2			
	PTCHD3			
	SLC5A1			
TNRC6C				
	PRAP1			
	FRMD4A			
	C1QTNF8			

(1b) BWS-MLMDs

BWS-MLMD1	BWS-MLMD2	BWS-MLMD3	BWS-MLMD4	BWS-MLMD5
DIRAS3	*DIRAS3*	*DIRAS3*	*DIRAS3*	*DIRAS3*
HYMAI;PLAGL1	*HYMAI;PLAGL1*	*HYMAI;PLAGL1*		
	JAKMIP1	*JAKMIP1*	*JAKMIP1*	
	ERLIN2;LOC728024	*ERLIN2;LOC728024*	*ERLIN2;LOC728024*	*ERLIN2;LOC728024*
	LOC100130522	*LOC100130522*		
		IGF1R	*IGF1R*	*IGF1R*
WRB	*WRB*	*WRB*	*WRB*	
	FAM50B			*FAM50B*
KCNQ1;KCNQ1OT1	*KCNQ1;KCNQ1OT1*	*KCNQ1;KCNQ1OT1*	*KCNQ1;KCNQ1OT1*	*KCNQ1;KCNQ1OT1*
MEST;MESTIT1	*MEST;MESTIT1*	*MEST;MESTIT1*		
ZNF331	*ZNF331*		*ZNF331*	
	NHP2L1			*NHP2L1*
	L3MBTL		*L3MBTL*	
		HM13;PSIMCT-1	*HM13;PSIMCT-1*	
	GNAS;GNASAS	*GNAS;GNASAS*	*GNAS;GNASAS*	
	GNAS	*GNAS*	*GNAS*	
	GLP2R	*GLP2R*		
		GRB10		
	SVOPL			*SVOPL*
	PEG10;SGCE			
		NPAS3		
LOC728448	*LOC728448*	*LOC728448*		
	ELANE	*ELANE*		
	DHX16	*DHX16*		
	MAFG	*MAFG*		
	MAP3K6	*MAP3K6*		
	CBFA2T3	*CBFA2T3*		
	GP9	*GP9*		
	PRTN3	*PRTN3*		
	BLCAP;NNAT	*BLCAP;NNAT*		
	KCNAB3	*KCNAB3*		
INPP5F		*INPP5F*		
	AZU1	*AZU1*		
			MAGEL2	
	GRB7	*GRB7*		
			SNRPN;SNURF	
	SEPT9			
	PEG10			
	CHD7	*CHD7*		
	RARA	*RARA*		
		HOXB7	*HOXB7*	
		DDR1	*DDR1*	
		RBMXL2		
	FGR	*FGR*		
	SLFN13	*SLFN13*		
	PTCHD3			
	CXXC5	*CXXC5*		
			SLC5A1	

	P2RX1	*P2RX1*		
C1orf65				
	MAD1L1	*MAD1L1*		
	FCGRT	*FCGRT*		
PRKCZ	*PRKCZ*			
	HORMAD2	*HORMAD2*		
	TNRC6C			
			MIMT1;PEG3;ZIM2	
	IFFO1			
		HSPA7		
		MACROD1		
		COL11A2		
	MAN2B1			
		MIR193A		
		MIR193B		
	PRKAG2			
		BMP7		
		ADO		
		ECEL1P2		
		APLP2		
		FOXI2		
	MARCH8			
	LTBR			
		FAM163A		
	PILRA			
		LHX9		
		PPT2		
	FXYD1			
		RETN		
	DENND3			
		INHBB		
		CSNK1E		
		ALX4		
		ZNF578		
	CDH5			
		FBXO2;FBXO44		
	MAEA			
	SHC1			
	ENTPD1			
		LOC254559		
		STRA6		
		FCHO1		
	SYNJ2			
	SPRED2			
	IQCE			
		UCN		
	BRD4			
		MT1M		
		SH2B2		
	TMEM105			

	OSBPL5			
		TRAPPC9		
		DKK4		
		WDR52		
		EGFL7		
		GRM2		
	C11orf41			
		ETV3L		
		PRDM16		
		ANGPT2;MCPH1		
		MT1G;MT1H		
	TRIM39			
		AMT;NICN1		
		LYPD3		
		HLA-G		
		OTOP3		
		TMEM119		
		PLA2G3		
	IFNGR2			
		GABBR1		
		LAMB2		
	SKI			
	ADRBK1			
		HERC3;NAP1L5		
	LTBP1			
		LPIN3		
		C17orf64		
	MS4A3			
	PNPLA2			
	TIAM2			
	SNORA30;SRCAP			
		PURG;WRN		
		ZIC1		
		SNX31		
		FBXL18		
	ZNF516			
		EIF4E3;GPR27		
	TRPM2			
		SIX2		
	RNF144A			
	ESPNL			
		COCH		
		ESPNP		
		MIR183;MIR96		
		C6orf27		
	C16orf68			
		FGF2		
		BHMT		
		BCAN		
	ALDH4A1			

	MPO			
	KRT17			
		PAX6		
		ABCG4		
	GPR97			
		TRIM2		
	ITGAE			
		FGF20		
	NADK			
	HOXB5;LOC404266			
		CLDN14		
	EPHA8			
	AMPD3			
		EPHA2		
		RIMS3		
		KLHDC7B		
		SHROOM1		
		SNED1		
	MXD3			
	NCOR2			
	BTBD12			
		HOXB6;LOC404266		
		CCDC105		
	FOXK1			
		CHRNB1		
		EPB49		
		TRADD		
		SARM1		
		KRT72		
		KRTCAP3;NRBP1		
		MGC12982		
		C17orf28		
	S100A8			
	WDR20			
		SEZ6		
		FBXO27		
		DES		
	AGTRAP			
		FGF17		
		C4orf38		
		SLC35C1		
		MARCKSL1		
	SLC20A2			
	FBXO47			
		KCNG3		
	TSKS			
		ASCL2		
	CSTA			
		FOXD2		
		DLL1		

	FES			
	TRIM61			
		KIAA2013		
	C10orf95			
		ADAM5P		
	RAB11FIP3			
		LRP1		
		RGS12		
		C13orf26		
		LRP5		
		WWTR1		
		DLEU7		
		LRRC32		
	TBCD			
		WNT6		
		TP73		
	RIN2			
		KRTCAP3		
		CHRD		
	C18orf1			
		LSP1		
	AATK			
	MGRN1			
	LOC404266			
		LPAR5		
	DTX1			
		TRIL		
		CMYA5		
		RNF39		
	SLFN12L			
		ABAT		
		LOC100129637		
		KCNA3		
	SEMA6B			
		SEC31B		
		C14orf48		
		CCDC42B		
		PLBD1		
	CEBPE			
		LPIN1		
		SPI1		
		PNMAL2		
		FOXD2;MGC12982		
	CPNE6			
		LOC146880		
	IL1R2			
	LPCAT1			
		ECEL1		
		PDXDC1		
		GDNF		

		PLEC1		
	ALDH3B1			
	TRIM27			
		SLC24A4		
	MEST			
		SYNGAP1		
		HRASLS5		
		DMKN		
		LOC100130872-SPON2;SPON2		
		SNRPF		
	RASA3			
	SBNO2			
	GFI1			
	NOTCH4			
	CALR			
		PPT2;PRRT1		
		PURA		
	FUT4			
		SIGLEC9		
	ZBTB47			
		DIO3;MIR1247		
	HLA-DMB			
		CCDC19		
		PRRT1		

Supplementary Table 2. Genomic regions in four TND-SIMPLE cases, as identified by the single sample analysis pipeline. Probes with M-values between −1 and +1 in controls and relative hypomethylation in patients with a p-values of <0.05 were identified. This subset was further filtered by minimal criteria for a hypomethylated locus, that is, a minimum 3 consecutive hypomethylated probes spaced by <2000 nucleotides. Candidate regions that meet these criteria in TND-SIMPLE cases are listed in the tables.

TND-SIMPLE1	TND-SIMPLE2	TND-SIMPLE3	TND-SIMPLE4
HYMAI; PLAGL1	*HYMAI; PLAGL1*	*HYMAI; PLAGL1*	*HYMAI; PLAGL1*
	GLP2R	*GLP2R*	
		GNAS	
	TRIM15		
	GATS; PVRIG		
	RASSF5		
	CD247		
	SEPT9		
	DIABLO		
	TSHR		
	BCL9L		
	KIAA1949		
	CLSTN1		
	TNFRSF18		
	ANKRD11		
	PTPRCAP		

	CYGB		
	STAT3		
	DENND2D		
	WIBG		
	MAD1L1		
	NA		
	PIK3CD		
	RUNX3		
	RPS6KB2		
	HFM1		
	SLCO5A1		
	MEST; MESTIT1		
	ZAP70		
	DGKA		
	PDCD1		
	H19		
	IFITM1		
	DDIT4		
	PEG10; SGCE		
	CCDC57		
	KLF2		
	PSMB8		
	ZFP36L1		
	ABI3; GNGT2		
	CHRNA4		
	LTA		
	MEG3		
	PRF1		
	KCNQ1; KCNQ1OT1		
	ACOT7		
	PBX2		
	CORO1B; PTPRCAP		
	FSD1		
	LGALS3BP		
	LIME1		
	POU2AF1		
	CD3D; CD3G		
	TC2N		
	LCK		
	GNG7		
	FLOT1		
	GPR133		
	TMIGD2		
	CD3D		
	SH3TC1		

Supplementary Table 3. Genomic regions in three BWS-SIMPLE cases, as identified by the single sample analysis pipeline. Probes with M-values between −1 and +1 in controls and relative hypomethylation in patients with a p-values of <0.05 were identified. This subset was further filtered by minimal criteria for a hypomethylated locus, that is, a minimum 3 consecutive hypomethylated probes spaced by <2000 nucleotides. Candidate regions that meet these criteria in BWS-SIMPLE cases are listed in the tables.

BWS-SIMPLE 1	BWS-SIMPLE2	BWS-SIMPLE3
KCNQ1; KCNQ1OT1	*KCNQ1; KCNQ1OT1*	*KCNQ1; KCNQ1OT1*
		GNAS
		MEG3
		ERLIN2; LOC728024
	GJD3	

Supplementary Table 4. Shared sites detected by the single sample analysis pipeline in biological replicates. DNA from TND-MLMD1 was independently processed in two different batches. Probes with M-values between −1 and +1 in controls and relative hypomethylation in patients with a p-values of <0.05 were identified. This subset was further filtered by minimal criteria for a hypomethylated locus, that is, a minimum 3 consecutive hypomethylated probes spaced by <2000 nucleotides. The p-values were determined by Fisher's combined p-value method for independent tests. Here, ID = Imprinting Disorder; DMR = Differentially Methylated Region.

		TND-MLMD1 batch1				TND-MLMD1 batch2		
	Chr	Gene name	Probe region	No. of probes	P-value	Probe region	No. of probes	P-value
Imprinted-associated with ID	6	*HYMAI;PLAGL1*	144328482-144329922	16	1.07E-102	144328482-144329909	15	7.49E-16
	7	*MEST;MESTIT1*	130130122-130131931	43	7.48E-276	130130288-130132161	41	6.56E-273
	11	*KCNQ1;KCNQ1OT1*	2720229-2722195	31	0	2720229-2722195	31	3.84E-315
Imprinted-not associated with ID	1	*DIRAS3*	68515788-68517273	20	2.95E-171	68515788-68517273	20	1.52E-146
	6	*FAM50B*	3849235-3849702	18	7.81E-84	3849331-3849702	15	2.83E-33
	10	*INPP5F*	121578384-121578639	3	1.54E-05			
	15	*IGF1R*	99408636-99409506	6	7.29E-14	99408804-99409506	5	1.62E-09
	19	*ZNF331*	54040774-54041999	9	1.84E-40	54040813-54041856	7	5.71E-51
	20	*L3MBTL*	42142417-42143502	22	2.50E-201	42142417-42143502	25	4.99E-198
Novel candidate DMRs	4	*JAKMIP1*	6107131-6107339	4	1.25E-06	6107021-6107339	5	4.53E-15
	5	*TMEM232*	110062343-110062473	5	1.16E-06			
	8	*LOC728024;ERLIN2*	37605359-37605978	7	4.86E-42	37605552-37605978	4	2.33E-21
	17	*GLP2R*	9729250-9729424	6	1.44E-10			
	17	*TNRC6C*	76036514-76037562	7	1.88E-16	76036514-76037562	7	6.13E-13
	18	*LOC100130522*	77905298-77905947	8	1.44E-45	77905298-77905947	9	3.35E-38

Supplementary Table 5. Identified regions of TND-MLMD and BWS-MLMD cases using the single sample analysis pipeline with varying controls size. . Tables 5(a) and 5(b) represent the results for the five TND-MLMD and three BWS-MLMD cases, respectively, against 5, 10, 20, 30, 40, and 50 controls Probes with M-values between −1 and +1 in controls and relative hypomethylation in patients with a p value of <0.05 were identified. This subset was further filtered by minimal criteria for a hypomethylated locus, that is, a minimum 3 consecutive hypomethylated probes spaced by <2000 nucleotides. Candidate regions that meet these criteria are indicated in red.

5(a)

		TND-MLMD1						TND-MLMD2						TND-MLMD3						TND-MLMD4						TND-MLMD5					
		control size						control size						control size						control size						control size					
Chr	Gene	5	10	20	30	40	50	5	10	20	30	40	50	5	10	20	30	40	50	5	10	20	30	40	50	5	10	20	30	40	50
1	*DIRAS3*																														
4	*JAKMIP1*																														
5	*SEPT8*																														
6	*FAM50B*																														
6	*HYMAI;PLAGL1*																														
7	*SVOPL*																														
7	*MEST;MESTIT1*																														
7	*PEG10;SGCE*																														
7	*GRB10*																														
8	*ERLIN2;LOC728024*																														
10	*FRMD4A*																														
10	*PRAP1*																														
10	*PTCHD3*																														
11	*KCNQ1;KCNQ1OT1*																														
11	*RBMXL2*																														
13	*RB1*																														
14	*NPAS3*																														
15	*SNRPN;SNURF*																														
15	*IGF1R*																														
15	*MAGEL2*																														

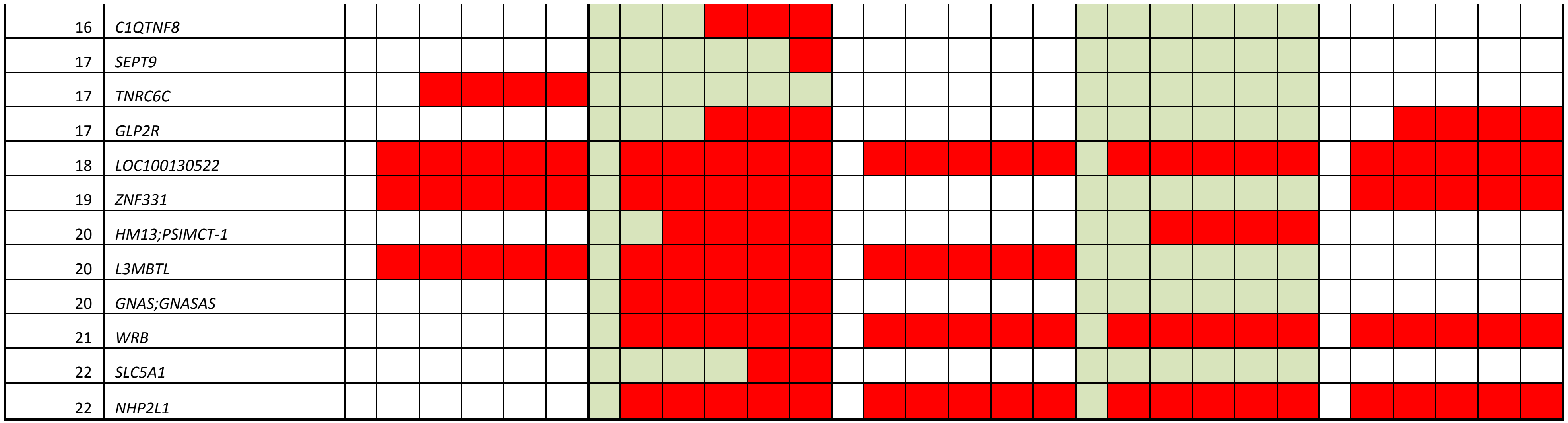
16 C1QTNF8
17 SEPT9
17 TNRC6C
17 GLP2R
18 LOC100130522
19 ZNF331
20 HM13;PSIMCT-1
20 L3MBTL
20 GNAS;GNASAS
21 WRB
22 SLC5A1
22 NHP2L1

5(b)

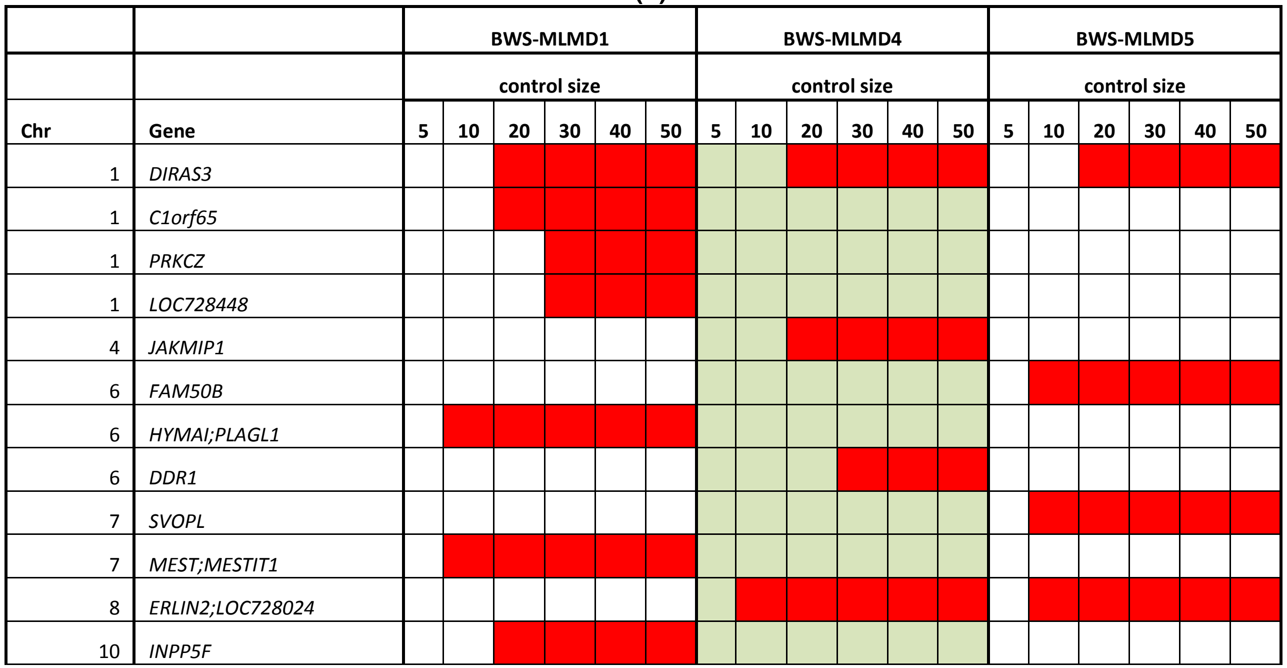
BWS-MLMD1
BWS-MLMD4
BWS-MLMD5
control size
Chr
Gene
5 10 20 30 40 50
1 DIRAS3
1 C1orf65
1 PRKCZ
1 LOC728448
4 JAKMIP1
6 FAM50B
6 HYMAI;PLAGL1
6 DDR1
7 SVOPL
7 MEST;MESTIT1
8 ERLIN2;LOC728024
10 INPP5F

11	*KCNQ1;KCNQ1OT1*
15	*SNRPN;SNURF*
15	*IGF1R*
15	*NDN*
15	*MAGEL2*
17	*HOXB7*
19	*ZNF331*
19	*MIMT1;PEG3;ZIM2*
20	*HM13;PSIMCT-1*
20	*GNAS;GNASAS*
20	*L3MBTL*
21	*WRB*
22	*SLC5A1*
22	*NHP2L1*

Supplementary Table 6. Effect size calculation for Crawford-Howell t-test in imprinting disorders. Here, the effect sizes are presented over *PLAGL1* imprinting control region with 95% confidence interval for TND-MLMD3.

Genomic Location chr6 (hg19)	Control size = 05	Control size = 10	Control size = 20	Control size = 30	Control size = 40	Control size = 50
144329473	-13.319 (-22.268 to -4.581)	-13.345 (-19.430 to -7.274)	-14.089 (-18.546 to -9.623)	-14.213 (-17.863 to -10.554)	-15.179 (-18.543 to -11.806)	-15.463 (-18.523 to -12.395)
144386416	-0.190 (-1.065 to 0.706)	-0.283 (-0.909 to 0.357)	-0.089 (-0.527 to 0.351)	-0.515 (-0.892 to -0.130)	-0.245 (-0.558 to 0.071)	-0.068 (-0.345 to 0.210)
144329766	-9.046 (-15.153 to -3.069)	-11.585 (-16.875 to -6.304)	-11.815 (-15.560 to -8.061)	-12.632 (-15.880 to -9.376)	-11.893 (-14.536 to -9.243)	-13.713 (-16.429 to -10.989)
144329052	-13.466 (-22.513 to -4.633)	-15.384 (-22.391 to -8.396)	-16.475 (-21.681 to -11.260)	-16.331 (-20.520 to -12.132)	-15.625 (-19.088 to -12.154)	-19.242 (-23.045 to -15.431)
144329780	-15.396 (-25.730 to -5.311)	-13.945 (-20.300 to -7.604)	-16.704 (-21.981 to -11.417)	-14.601 (-18.350 to -10.843)	-14.384 (-17.574 to -11.187)	-15.239 (-18.255 to -12.216)
144329331	-8.218 (-13.775 to -2.772)	-7.424 (-10.844 to -4.002)	-7.914 (-10.442 to -5.377)	-7.578 (-9.546 to -5.602)	-8.895 (-10.881 to -6.901)	-9.368 (-11.234 to -7.496)
144329789	-8.406 (-14.089 to -2.840)	-9.858 (-14.371 to -5.351)	-10.843 (-14.284 to -7.394)	-10.590 (-13.320 to -7.853)	-12.963 (-15.840 to -10.078)	-12.339 (-14.787 to -9.886)
144329485	-10.626 (-17.782 to -3.630)	-10.663 (-15.538 to -5.796)	-12.486 (-16.441 to -8.522)	-13.044 (-16.396 to -9.682)	-13.743 (-16.792 to -10.686)	-14.270 (-17.095 to -11.437)
144329909	-19.247 (-32.150 to -6.660)	-13.840 (-20.149 to -7.546)	-9.724 (-12.816 to -6.624)	-12.783 (-16.069 to -9.488)	-7.076 (-8.667 to -5.478)	-9.360 (-11.224 to -7.489)
144328482	-21.470 (-35.857 to -7.438)	-22.586 (-32.853 to -12.350)	-25.334 (-33.323 to -17.332)	-22.650 (-28.451 to -16.837)	-20.523 (-25.064 to -15.972)	-23.970 (-28.703 to -19.227)
144329829	-17.447 (-29.148 to -6.030)	-19.751 (-28.734 to -10.795)	-24.289 (-31.950 to -16.616)	-20.136 (-25.295 to -14.965)	-17.954 (-21.929 to -13.970)	-23.917 (-28.640 to -19.185)
144329732	-2.725 (-4.721 to -0.702)	-2.854 (-4.277 to -1.406)	-3.314 (-4.440 to -2.174)	-3.202 (-4.091 to -2.303)	-3.779 (-4.667 to -2.885)	-3.693 (-4.469 to -2.910)
144329172	-13.161 (-22.004 to -4.525)	-15.275 (-22.232 to -8.335)	-13.214 (-17.397 to -9.022)	-15.080 (-18.950 to -11.200)	-14.087 (-17.211 to -10.955)	-16.859 (-20.194 to -13.517)
144329887	-22.207 (-37.088 to -7.696)	-17.809 (-25.913 to -9.728)	-10.413 (-13.719 to -7.097)	-10.250 (-12.893 to -7.599)	-11.259 (-13.763 to -8.748)	-9.929 (-11.905 to -7.947)
144328917	-24.650 (-41.162 to -8.549)	-21.641 (-31.480 to -11.832)	-21.309 (-28.033 to -14.574)	-20.179 (-25.350 to -14.998)	-13.875 (-16.953 to -10.789)	-23.252 (-27.844 to -18.651)
144329802	-6.429 (-10.807 to -2.126)	-8.371 (-12.215 to -4.528)	-9.267 (-12.216 to -6.309)	-9.244 (-11.633 to -6.848)	-1.394 (-1.826 to -0.952)	-10.330 (-12.385 to -8.270)

Supplementary Table 7. The performance of three t-tests (one-sample, Weisberg, Crawford-Howell t-tests) around the *PLAGL1* region in TND-MLMD1. The point estimate represents the estimated percentage of the control population that would be expected to obtain lower score than the case and 95% confidence interval of point estimates were calculated from the noncentrality parameter from a noncentral t-distribution. Here, CH = Crawford-Howell t-test, WB = Weisberg t-test, and OS = One-sample t-test. Additionally, Chr = chromosome, Map information = genomic location (hg19), ILMNID = Illumina Infinium HumanMethylation450 array probe ID, Mean = Control mean, and SD = control standard deviation.

			Control group			T-value			P-value					Estimated percentage of the control population obtaining a lower score than the case	
ILMNIND	Chr	Map Information	Mean	SD	Case score	CH.t	WB.t	OS.t	CH.p	WB.p	OS.p	CH.Point	WB.Point	OS.Point	(95%CI)
cg13607311	6	144262258	2.832	0.225	3.017	0.813	0.812	-5.803	0.21	0.421	4.70E-07	21	42.1	4.70E-05	(12.744 to 30.980)
cg01659632	6	144262297	3.131	0.294	3.063	-0.231	-0.231	1.651	0.409	0.818	0.105	40.9	81.8	10.5	(30.385 to 51.939)
cg06271237	6	144263369	0.164	0.394	0.603	1.102	1.102	-7.869	0.138	0.276	3.03E-10	13.8	27.6	3.03E-08	(7.173 to 22.495)
cg26261298	6	144263435	-2.113	0.549	-1.275	1.511	1.51	-10.787	0.069	0.138	1.53E-14	6.9	13.8	1.53E-12	(2.673 to 13.296)
cg12419863	6	144263450	-0.481	0.502	0.162	1.269	1.269	-9.066	0.105	0.21	4.70E-12	10.5	21	4.70E-10	(4.908 to 18.325)
cg21776667	6	144281452	2.46	0.203	2.62	0.779	0.779	-5.562	0.22	0.44	1.10E-06	22	44	0.00011	(13.539 to 32.068)
cg18316621	6	144283348	2.917	0.233	2.797	-0.511	-0.51	3.646	0.306	0.612	0.001	30.6	61.2	0.1	(20.937 to 41.371)
cg01445838	6	144286805	1.173	0.545	1.881	1.287	1.287	-9.194	0.102	0.204	3.04E-12	10.2	20.4	3.04E-10	(4.704 to 17.913)
cg17870553	6	144292355	2.507	0.201	2.632	0.617	0.617	-4.409	0.27	0.54	5.67E-05	27	54	0.00567	(17.772 to 37.543)
cg27577583	6	144294847	2.96	0.197	3.046	0.433	0.433	-3.093	0.333	0.667	0.003	33.3	66.7	0.3	(23.400 to 44.233)
cg09730369	6	144328421	1.203	0.156	-0.615	-11.574	-11.572	82.655	6.66E-16	1.78E-15	0	6.66E-14	1.78E-13	0	(1.36E-42 to 7.11E-19)
cg17865602	6	144328482	0.723	0.122	-2.192	-23.736	-23.731	169.506	0	0	0	0	0	0	(3.03E-178 to 1.38E-79)
cg25350411	6	144328917	0.125	0.129	-2.876	-23.023	-23.018	164.415	0	0	0	0	0	0	(1.00E-167 to 6.95E-75)
cg07077459	6	144329052	0.346	0.132	-2.191	-19.051	-19.047	136.051	0	0	0	0	0	0	(5.61E-115 to 2.69E-51)
cg22378065	6	144329172	0.351	0.14	-2.005	-16.692	-16.689	119.205	0	0	0	0	0	0	(2.40E-88 to 2.22E-39)
cg10007452	6	144329331	0.062	0.132	-1.173	-9.275	-9.273	66.237	1.15E-12	2.82E-12	0	1.15E-10	2.82E-10	0	(2.16E-27 to 4.87E-12)
cg00702231	6	144329473	0.565	0.169	-2.052	-15.31	-15.307	109.339	0	0	0	0	0	0	(2.25E-74 to 4.00E-33)
cg12757684	6	144329485	0.023	0.144	-2.026	-14.127	-14.124	100.889	0	0	0	0	0	0	(2.32E-63 to 3.44E-28)
cg21952820	6	144329732	0.249	0.358	-1.075	-3.655	-3.654	26.103	0	0.001	0	0	0.1	0	(0.000 to 0.181)
cg05326984	6	144329766	0.096	0.164	-2.154	-13.575	-13.572	96.945	0	0	0	0	0	0	(1.60E-58 to 5.07E-26)

cg08263357	6	144329780	0.668	0.193	-2.278	-15.091	-15.088	107.769	0	0	0	0	0	0	(2.91E-72 to 3.53E-32)
cg11532302	6	144329789	0.148	0.168	-1.927	-12.219	-12.217	87.264	1.11E-16	2.22E-16	0	1.11E-14	2.22E-14	0	(1.86E-47 to 4.66E-21)
cg27216384	6	144329802	-0.09	0.149	-1.629	-10.228	-10.226	73.043	4.73E-14	1.21E-13	0	4.73E-12	1.21E-11	0	(2.72E-33 to 1.08E-14)
cg17895149	6	144329829	0.405	0.116	-2.365	-23.679	-23.675	169.104	0	0	0	0	0	0	(2.10E-177 to 3.29E-79)
cg23460430	6	144329887	0.789	0.184	-1.041	-9.832	-9.83	70.214	1.76E-13	4.43E-13	0	1.76E-11	4.43E-11	0	(9.04E-31 to 1.47E-13)
cg14161241	6	144329909	0.378	0.148	-1.009	-9.269	-9.267	66.194	1.18E-12	2.88E-12	0	1.18E-10	2.88E-10	0	(2.35E-27 to 5.05E-12)
cg03562868	6	144329922	1.14	0.209	0.334	-3.813	-3.813	27.233	0	0	0	0	0	0	(0.000 to 0.119)
cg20443501	6	144329962	1.207	0.137	0.727	-3.458	-3.458	24.697	0.001	0.001	0	0.1	0.1	0	(0.001 to 0.301)
cg04840930	6	144329997	2.184	0.12	1.644	-4.458	-4.457	31.838	2.41E-05	4.97E-05	0	0.00241	0.00497	0	(3.10E-06 to 0.018)
cg18593039	6	144330162	1.746	0.194	1.795	0.253	0.252	-1.803	0.401	0.802	0.077	40.1	80.2	7.7	(29.615 to 51.114)
cg23840797	6	144330233	2.624	0.152	2.381	-1.583	-1.583	11.308	0.06	0.12	2.89E-15	6	12	2.89E-13	(2.192 to 11.992)
cg00981250	6	144330345	3.108	0.143	3.064	-0.305	-0.305	2.178	0.381	0.762	0.034	38.1	76.2	3.4	(27.749 to 49.092)
cg02697107	6	144333219	1.763	0.202	1.852	0.435	0.435	-3.107	0.333	0.666	0.003	33.3	66.6	0.3	(23.338 to 44.162)
cg08256027	6	144334733	-2.165	0.313	-2.37	-0.649	-0.649	4.638	0.26	0.519	2.64E-05	26	51.9	0.00264	(16.874 to 36.421)
cg07018708	6	144334983	2.465	0.176	2.219	-1.384	-1.384	9.887	0.086	0.173	2.93E-13	8.6	17.3	2.93E-11	(3.707 to 15.785)
cg27016993	6	144382594	2.257	0.129	2.147	-0.84	-0.84	6.002	0.202	0.405	2.32E-07	20.2	40.5	2.32E-05	(12.110 to 30.095)
cg02566775	6	144382964	-0.058	0.318	0.722	2.43	2.43	-17.355	0.009	0.019	0	0.9	1.9	0	(0.131 to 2.920)
cg04232541	6	144384895	-3.576	0.163	-3.541	0.215	0.215	-1.535	0.415	0.831	0.131	41.5	83.1	13.1	(30.976 to 52.568)
cg07750706	6	144385261	-3.568	0.198	-3.652	-0.421	-0.421	3.007	0.338	0.676	0.004	33.8	67.6	0.4	(23.797 to 44.686)
cg04319464	6	144385609	-2.09	0.24	-1.904	0.767	0.767	-5.476	0.223	0.447	1.49E-06	22.3	44.7	0.000149	(13.832 to 32.464)
cg18144296	6	144385771	-2.85	0.174	-2.555	1.68	1.679	-11.995	0.05	0.1	4.44E-16	5	10	4.44E-14	(1.670 to 10.422)
cg15144236	6	144385792	-3.497	0.196	-3.304	0.971	0.971	-6.937	0.168	0.336	8.31E-09	16.8	33.6	8.31E-07	(9.412 to 26.126)
cg07507918	6	144385806	-2.839	0.144	-2.71	0.883	0.883	-6.304	0.191	0.382	7.95E-08	19.1	38.2	7.95E-06	(11.188 to 28.779)
cg21242697	6	144385893	-2.865	0.134	-2.905	-0.294	-0.294	2.102	0.385	0.77	0.041	38.5	77	4.1	(28.127 to 49.504)
cg23521444	6	144385917	-3.028	0.181	-2.986	0.234	0.234	-1.669	0.408	0.816	0.101	40.8	81.6	10.1	(30.294 to 51.842)
cg02926622	6	144386041	-3.074	0.196	-2.927	0.74	0.74	-5.285	0.231	0.463	2.89E-06	23.1	46.3	0.000289	(14.492 to 33.345)
cg09307468	6	144386093	-2.764	0.286	-2.281	1.673	1.673	-11.947	0.05	0.101	4.44E-16	5	10.1	4.44E-14	(1.702 to 10.526)
cg06493166	6	144386146	-2.053	0.381	-1.47	1.514	1.514	-10.812	0.068	0.137	1.42E-14	6.8	13.7	1.42E-12	(2.648 to 13.231)
cg00814597	6	144386148	-1.942	0.23	-1.539	1.738	1.738	-12.414	0.044	0.089	0	4.4	8.9	0	(1.406 to 9.541)
cg16334244	6	144386231	-1.287	0.242	-1.153	0.55	0.55	-3.927	0.292	0.585	0	29.2	58.5	0	(19.738 to 39.943)
cg12444861	6	144386275	0.286	0.135	0.361	0.553	0.552	-3.946	0.292	0.583	0	29.2	58.3	0	(19.658 to 39.846)

cg02849309	6	144386280	0.922	0.168	1.145	1.316	1.316	-9.4	0.097	0.194	1.51E-12	9.7	19.4	1.51E-10	(4.388 to 17.261)
cg01595870	6	144386416	0.844	0.162	0.833	-0.067	-0.067	0.481	0.473	0.947	0.633	47.3	94.7	63.3	(36.499 to 58.311)
cg09293391	6	144386418	0.619	0.161	0.737	0.725	0.725	-5.176	0.236	0.472	4.22E-06	23.6	47.2	0.000422	(14.880 to 33.856)
cg25291978	6	144386422	2.198	0.295	2.365	0.561	0.561	-4.004	0.289	0.578	0	28.9	57.8	0	(19.416 to 39.555)
cg04895233	6	144386457	1.678	0.209	1.564	-0.541	-0.541	3.865	0.295	0.591	0	29.5	59.1	0	(20.001 to 40.258)
cg25535418	6	144386474	2.423	0.3	2.395	-0.093	-0.093	0.663	0.463	0.926	0.511	46.3	92.6	51.1	(35.531 to 57.322)
cg10254692	6	144386515	0.586	0.235	0.756	0.714	0.714	-5.098	0.239	0.479	5.53E-06	23.9	47.9	0.000553	(15.159 to 34.222)
cg20931541	6	144386528	2.111	0.306	2.015	-0.31	-0.31	2.216	0.379	0.758	0.031	37.9	75.8	3.1	(27.564 to 48.890)
cg21113768	6	144386848	2.548	0.231	2.79	1.034	1.034	-7.386	0.153	0.306	1.68E-09	15.3	30.6	1.68E-07	(8.278 to 24.335)
cg24750627	6	144387124	1.516	0.155	1.405	-0.71	-0.71	5.074	0.24	0.481	6.01E-06	24	48.1	0.000601	(15.247 to 34.337)
cg14143954	6	144388928	2.251	0.171	2.401	0.869	0.868	-6.203	0.195	0.389	1.14E-07	19.5	38.9	1.14E-05	(11.491 to 29.215)

Supplementary Table 8. The performance of three t-tests (one-sample, Weisberg, Crawford-Howell t-tests) around the *KCNQ1OT1* region in BWS-MLMD1. The point estimate represents the estimated percentage of the control population that would be expected to obtain lower score than the case and 95% confidence interval of point estimates were calculated from the noncentrality parameter from a noncentral t-distribution. Here, CH = Crawford-Howell t-test, WB = Weisberg t-test, and OS = One-sample t-test. Additionally, Chr = chromosome, Map information = genomic location (hg19), ILMNID = Illumina Infinium HumanMethylation450 array probe ID, Mean = Control mean, and SD = control standard deviation.

			Control group			T-value			P-value			Estimated percentage of the control population obtaining a lower score than the case			
ILMNID	Chr	Map Information	Mean	SD	Case score	CH.t	WB.t	OS.t	CH.p	WB.p	OS.p	CH.Point	WB.Point	OS.Point	(95% CI)
cg21190593	11	2451058	1.109	0.268	0.864	-0.906	-0.905	6.467	0.185	0.185	4.44E-08	18.5	18.5	4.44E-06	(10.710 to 28.081)
cg19936757	11	2452218	1.295	0.146	1.543	1.684	1.684	-12.029	0.049	0.0495	4.44E-16	4.9	4.95	4.44E-14	(1.647 to 10.348)
cg14408823	11	2458481	1.831	0.157	1.625	-1.296	-1.296	9.257	0.1005	0.1005	2.45E-12	10.05	10.05	2.45E-10	(4.605 to 17.711)
cg10425885	11	2458734	2.354	0.142	2.497	0.998	0.998	-7.128	0.1615	0.1615	4.20E-09	16.15	16.15	4.20E-07	(8.917 to 25.355)
cg04055211	11	2459151	2.359	0.22	2.448	0.401	0.4	-2.861	0.345	0.3455	0.006	34.5	34.55	0.6	(24.475 to 45.455)
cg20429584	11	2461432	2.683	0.207	2.484	-0.954	-0.954	6.811	0.1725	0.1725	1.30E-08	17.25	17.25	1.30E-06	(9.748 to 26.641)
cg15033776	11	2463739	2.998	0.31	2.888	-0.353	-0.353	2.522	0.3625	0.363	0.015	36.25	36.3	1.5	(26.078 to 47.251)
cg20533553	11	2464822	1.829	0.167	1.589	-1.422	-1.422	10.157	0.0805	0.081	1.20E-13	8.05	8.1	1.20E-11	(3.368 to 15.008)
cg03371125	11	2464845	2.294	0.151	2.216	-0.512	-0.512	3.658	0.3055	0.3055	0.001	30.55	30.55	0.1	(20.885 to 41.309)
cg01178971	11	2464851	1.294	0.278	1.617	1.147	1.147	-8.193	0.1285	0.1285	9.69E-11	12.85	12.85	9.69E-09	(6.495 to 21.311)
cg04902871	11	2464954	1.479	0.271	1.44	-0.142	-0.142	1.013	0.444	0.444	0.316	44.4	44.4	31.6	(33.683 to 55.413)
cg19698309	11	2464959	2.623	0.184	2.399	-1.207	-1.207	8.619	0.1165	0.1165	2.19E-11	11.65	11.65	2.19E-09	(5.678 to 19.816)
cg23267890	11	2464970	0.416	0.196	0.282	-0.676	-0.676	4.83	0.251	0.251	1.38E-05	25.1	25.1	0.00138	(16.145 to 35.494)
cg15932136	11	2465028	0.465	0.194	0.683	1.114	1.113	-7.954	0.1355	0.1355	2.25E-10	13.55	13.55	2.25E-08	(6.992 to 22.183)
cg10678459	11	2465103	1.958	0.243	1.671	-1.173	-1.173	8.38	0.123	0.1235	5.05E-11	12.3	12.35	5.05E-09	(6.127 to 20.648)
cg11025829	11	2465136	-1.149	0.159	-0.783	2.275	2.275	-16.248	0.0135	0.0135	0	1.35	1.35	0	(0.236 to 3.897)
cg11363972	11	2465249	0.353	0.176	0.013	-1.911	-1.911	13.648	0.031	0.031	0	3.1	3.1	0	(0.825 to 7.279)
cg24079038	11	2465279	-0.411	0.171	-0.559	-0.853	-0.852	6.088	0.199	0.199	1.71E-07	19.9	19.9	1.71E-05	(11.842 to 29.716)
cg00283576	11	2465351	-2.993	0.158	-3.005	-0.073	-0.073	0.52	0.471	0.471	0.605	47.1	47.1	60.5	(36.286 to 58.094)
cg04104132	11	2465440	-1.679	0.161	-1.434	1.502	1.502	-10.729	0.0695	0.07	1.87E-14	6.95	7	1.87E-12	(2.732 to 13.448)

cg23623667	11	2465485	-2.41	0.247	-2.54	-0.52	-0.52	3.711	0.303	0.303	0.001	30.3	30.3	0.1	(20.657 to 41.040)
cg19779211	11	2465491	-2.109	0.169	-1.984	0.734	0.734	-5.241	0.233	0.2335	3.37E-06	23.3	23.35	0.000337	(14.648 to 33.551)
cg04204548	11	2465887	-3.12	0.218	-3.217	-0.439	-0.439	3.132	0.3315	0.3315	0.003	33.15	33.15	0.3	(23.221 to 44.028)
cg09336947	11	2465950	-3.316	0.538	-3.274	0.077	0.077	-0.551	0.4695	0.4695	0.584	46.95	46.95	58.4	(36.125 to 57.929)
cg05302416	11	2466037	-2.428	0.254	-2.134	1.144	1.144	-8.17	0.129	0.129	1.05E-10	12.9	12.9	1.05E-08	(6.542 to 21.395)
cg07127259	11	2466042	-3.108	0.254	-2.639	1.829	1.829	-13.062	0.0365	0.037	0	3.65	3.7	0	(1.068 to 8.294)
cg26417642	11	2466095	-3.593	0.222	-3.518	0.336	0.336	-2.401	0.369	0.369	0.02	36.9	36.9	2	(26.659 to 47.895)
cg06533200	11	2466138	-4.252	0.343	-4.024	0.657	0.657	-4.695	0.257	0.257	2.19E-05	25.7	25.7	0.00219	(16.658 to 36.148)
cg05760393	11	2466141	-4.033	0.395	-4.208	-0.44	-0.44	3.145	0.331	0.331	0.003	33.1	33.1	0.3	(23.165 to 43.964)
cg17575811	11	2466409	-2.76	0.227	-2.858	-0.425	-0.425	3.036	0.3365	0.3365	0.004	33.65	33.65	0.4	(23.660 to 44.530)
cg13071812	11	2466788	-2.893	0.112	-2.875	0.155	0.155	-1.11	0.4385	0.4385	0.272	43.85	43.85	27.2	(33.173 to 54.882)
cg05418487	11	2466889	-3.502	0.164	-3.474	0.166	0.166	-1.184	0.4345	0.4345	0.242	43.45	43.45	24.2	(32.791 to 54.483)
cg12578166	11	2466909	-1.934	0.126	-1.295	5.011	5.01	-35.788	3.72E-06	3.89E-06	0	0.000372	0.000389	0	(6.20E-08 to 0.003)
cg08895013	11	2468332	-2.794	0.155	-2.07	4.638	4.637	-33.12	1.33E-05	1.37E-05	0	0.001325	0.00137	0	(9.12E-07 to 0.010)
cg13298691	11	2470675	3.488	0.286	3.903	1.436	1.435	-10.252	0.0785	0.079	8.75E-14	7.85	7.9	8.75E-12	(3.255 to 14.740)
cg18016139	11	2470969	2.795	0.19	2.944	0.774	0.774	-5.529	0.2215	0.2215	1.24E-06	22.15	22.15	0.000124	(13.653 to 32.222)
cg27364242	11	2471064	1.834	0.135	2.098	1.935	1.935	-13.821	0.0295	0.0295	0	2.95	2.95	0	(0.763 to 6.999)
cg10673351	11	2471097	2.512	0.183	2.324	-1.016	-1.016	7.255	0.1575	0.1575	2.67E-09	15.75	15.75	2.67E-07	(8.598 to 24.848)
cg06159548	11	2481370	2.064	0.151	2.272	1.373	1.373	-9.806	0.088	0.088	3.84E-13	8.8	8.8	3.84E-11	(3.814 to 16.024)
cg26524638	11	2481449	2.715	0.192	2.726	0.056	0.056	-0.397	0.478	0.478	0.693	47.8	47.8	69.3	(36.945 to 58.765)
cg03600094	11	2481475	1.994	0.222	2.349	1.58	1.579	-11.281	0.0605	0.0605	3.11E-15	6.05	6.05	3.11E-13	(2.215 to 12.058)
cg25976755	11	2481495	2.812	0.189	2.847	0.187	0.187	-1.339	0.426	0.426	0.187	42.6	42.6	18.7	(31.985 to 53.636)
cg17341170	11	2481545	2.694	0.149	2.719	0.165	0.165	-1.179	0.435	0.435	0.244	43.5	43.5	24.4	(32.814 to 54.507)
cg25513073	11	2481641	2.14	0.146	1.934	-1.398	-1.398	9.983	0.084	0.0845	2.13E-13	8.4	8.45	2.13E-11	(3.584 to 15.506)
cg03365111	11	2481857	2.888	0.196	2.901	0.065	0.065	-0.462	0.4745	0.4745	0.646	47.45	47.45	64.6	(36.599 to 58.413)
cg16145209	11	2481909	2.142	0.128	2.201	0.457	0.457	-3.267	0.3245	0.325	0.002	32.45	32.5	0.2	(22.613 to 43.328)
cg23968213	11	2481965	2.031	0.166	2.272	1.446	1.446	-10.325	0.0775	0.0775	6.88E-14	7.75	7.75	6.88E-12	(3.170 to 14.536)
cg03170016	11	2482142	1.382	0.134	1.479	0.722	0.722	-5.155	0.237	0.237	4.53E-06	23.7	23.7	0.000453	(14.954 to 33.953)
cg25741120	11	2482223	2.869	0.202	2.737	-0.647	-0.647	4.62	0.2605	0.2605	2.81E-05	26.05	26.05	0.00281	(16.944 to 36.509)
cg03118430	11	2482233	3.114	0.235	3.353	1.006	1.006	-7.184	0.1595	0.16	3.44E-09	15.95	16	3.44E-07	(8.776 to 25.131)
cg26709929	11	2482240	2.711	0.287	2.96	0.859	0.859	-6.134	0.1975	0.1975	1.45E-07	19.75	19.75	1.45E-05	(11.700 to 29.513)

cg17291157	11	2482284	2.36	0.227	2.474	0.498	0.498	-3.559	0.31	0.3105	0.001	31	31.05	0.1	(21.314 to 41.815)
cg07210454	11	2482303	2.254	0.288	2.563	1.064	1.064	-7.601	0.146	0.1465	7.81E-10	14.6	14.65	7.81E-08	(7.772 to 23.504)
cg13840782	11	2482394	3.042	0.246	2.797	-0.987	-0.987	7.047	0.1645	0.1645	5.60E-09	16.45	16.45	5.60E-07	(9.124 to 25.679)
cg09393494	11	2482401	2.347	0.167	2.192	-0.92	-0.92	6.573	0.181	0.181	3.04E-08	18.1	18.1	3.04E-06	(10.407 to 27.633)
cg25946538	11	2482531	1.631	0.123	1.422	-1.683	-1.683	12.022	0.0495	0.0495	4.44E-16	4.95	4.95	4.44E-14	(1.651 to 10.362)
cg11878045	11	2482536	1.894	0.153	1.478	-2.685	-2.685	19.176	0.005	0.005	0	0.5	0.5	0	(0.046 to 1.761)
cg25266888	11	2482594	2.264	0.219	2.008	-1.161	-1.161	8.29	0.1255	0.126	6.91E-11	12.55	12.6	6.91E-09	(6.302 to 20.966)
cg23801748	11	2482735	3.333	0.204	3.448	0.557	0.556	-3.974	0.29	0.2905	0	29	29.05	0	(19.541 to 39.705)
cg27639104	11	2482929	3.264	0.24	3.346	0.335	0.335	-2.393	0.3695	0.3695	0.021	36.95	36.95	2.1	(26.700 to 47.939)
cg24376802	11	2485050	2.301	0.137	2.332	0.226	0.226	-1.615	0.411	0.411	0.113	41.1	41.1	11.3	(30.568 to 52.134)
cg26504274	11	2485409	3.557	0.214	3.818	1.21	1.21	-8.643	0.116	0.116	2.02E-11	11.6	11.6	2.02E-09	(5.636 to 19.736)
cg01734338	11	2491231	1.889	0.247	1.89	0.003	0.003	-0.02	0.499	0.499	0.984	49.9	49.9	98.4	(38.972 to 60.807)
cg09916212	11	2498390	3.184	0.282	2.805	-1.327	-1.327	9.478	0.0955	0.0955	1.16E-12	9.55	9.55	1.16E-10	(4.273 to 17.019)
cg01298120	11	2499341	3.167	0.182	3.399	1.263	1.263	-9.023	0.106	0.1065	5.45E-12	10.6	10.65	5.45E-10	(4.978 to 18.464)
cg07618453	11	2502181	-2.183	0.245	-2.782	-2.426	-2.426	17.328	0.0095	0.0095	0	0.95	0.95	0	(0.133 to 2.941)
cg19670883	11	2502357	-1.985	0.275	-2.759	-2.79	-2.789	19.924	0.0035	0.004	0	0.35	0.4	0	(0.030 to 1.415)
cg05125641	11	2503306	2.209	0.198	1.919	-1.452	-1.452	10.372	0.0765	0.0765	5.91E-14	7.65	7.65	5.91E-12	(3.117 to 14.408)
cg17035132	11	2504160	2.395	0.138	2.595	1.439	1.439	-10.276	0.0785	0.0785	8.08E-14	7.85	7.85	8.08E-12	(3.227 to 14.672)
cg06838584	11	2504581	2.926	0.309	3.223	0.951	0.951	-6.792	0.173	0.173	1.39E-08	17.3	17.3	1.39E-06	(9.800 to 26.720)
cg23272088	11	2504813	2.913	0.297	3.54	2.091	2.091	-14.935	0.021	0.021	0	2.1	2.1	0	(0.454 to 5.393)
cg11652182	11	2542384	2.981	0.213	3.138	0.73	0.73	-5.217	0.2345	0.2345	3.66E-06	23.45	23.45	0.000366	(14.734 to 33.664)
cg04719766	11	2542580	2.98	0.206	2.593	-1.862	-1.862	13.297	0.0345	0.0345	0	3.45	3.45	0	(0.964 to 7.874)
cg17229197	11	2542688	2.696	0.195	2.932	1.196	1.196	-8.544	0.1185	0.119	2.85E-11	11.85	11.9	2.85E-09	(5.817 to 20.075)
cg12949760	11	2542862	-0.507	0.229	-1.563	-4.562	-4.561	32.581	1.71E-05	1.77E-05	0	0.001705	0.001765	0	(1.53E-06 to 0.013)
cg24073146	11	2543204	2.709	0.176	2.659	-0.281	-0.281	2.006	0.39	0.39	0.05	39	39	5	(28.603 to 50.022)
cg02097203	11	2548282	-2.992	0.229	-3.298	-1.322	-1.322	9.441	0.096	0.0965	1.31E-12	9.6	9.65	1.31E-10	(4.327 to 17.133)
cg12520982	11	2548920	1.877	0.144	2.122	1.688	1.688	-12.058	0.049	0.049	4.44E-16	4.9	4.9	4.44E-14	(1.628 to 10.286)
cg18450293	11	2548955	2.944	0.256	3.006	0.238	0.238	-1.697	0.4065	0.4065	0.096	40.65	40.65	9.6	(30.154 to 51.692)
cg25554751	11	2548989	2.758	0.148	2.622	-0.913	-0.913	6.518	0.183	0.183	3.70E-08	18.3	18.3	3.70E-06	(10.564 to 27.865)
cg04666029	11	2552843	-2.43	0.117	-2.22	1.774	1.773	-12.666	0.041	0.0415	0	4.1	4.15	0	(1.265 to 9.040)
cg26512142	11	2554198	-2.9	0.091	-2.865	0.39	0.39	-2.786	0.349	0.349	0.008	34.9	34.9	0.8	(24.824 to 45.848)

cg24089935	11	2554295	-3.53	0.248	-3.517	0.05	0.05	-0.36	0.48	0.48	0.72	48	48	72	(37.144 to 58.967)
cg16465939	11	2554410	-1.797	0.142	-2.401	-4.21	-4.209	30.062	0	0	0	0	0	0	(1.43E-05 to 0.038)
cg04608933	11	2554801	0.918	0.158	0.828	-0.568	-0.567	4.053	0.2865	0.2865	0	28.65	28.65	0	(19.214 to 39.310)
cg18729298	11	2555203	-1.681	0.177	-1.463	1.216	1.216	-8.682	0.115	0.115	1.76E-11	11.5	11.5	1.76E-09	(5.564 to 19.600)
cg07824422	11	2555406	-0.57	0.154	-0.856	-1.837	-1.837	13.121	0.036	0.036	0	3.6	3.6	0	(1.041 to 8.188)
cg02258534	11	2555497	0.437	0.185	0.665	1.224	1.224	-8.74	0.1135	0.1135	1.44E-11	11.35	11.35	1.44E-09	(5.462 to 19.406)
cg05898618	11	2555576	1.985	0.391	2.394	1.033	1.033	-7.378	0.1535	0.1535	1.73E-09	15.35	15.35	1.73E-07	(8.299 to 24.368)
cg19255554	11	2556743	1.547	0.152	1.403	-0.937	-0.937	6.693	0.1765	0.1765	1.98E-08	17.65	17.65	1.98E-06	(10.072 to 27.132)
cg07640406	11	2558029	2.386	0.172	2.625	1.372	1.372	-9.797	0.088	0.0885	3.96E-13	8.8	8.85	3.96E-11	(3.826 to 16.051)
cg27246251	11	2559736	2.316	0.129	2.436	0.92	0.92	-6.571	0.181	0.181	3.06E-08	18.1	18.1	3.06E-06	(10.412 to 27.640)
cg02335306	11	2559880	2.904	0.183	3.114	1.141	1.141	-8.148	0.1295	0.13	1.13E-10	12.95	13	1.13E-08	(6.586 to 21.472)
cg22296808	11	2560641	2.051	0.136	1.918	-0.974	-0.974	6.956	0.1675	0.1675	7.76E-09	16.75	16.75	7.76E-07	(9.362 to 26.049)
cg07549149	11	2560783	2.444	0.267	2.173	-1.003	-1.002	7.16	0.1605	0.1605	3.75E-09	16.05	16.05	3.75E-07	(8.837 to 25.227)
cg22047766	11	2560864	2.711	0.181	2.544	-0.914	-0.913	6.524	0.1825	0.183	3.63E-08	18.25	18.3	3.63E-06	(10.547 to 27.840)
cg03030994	11	2562310	2.347	0.176	2.46	0.637	0.637	-4.549	0.2635	0.2635	3.56E-05	26.35	26.35	0.00356	(17.220 to 36.855)
cg26929889	11	2562496	3.129	0.183	3.173	0.239	0.239	-1.704	0.406	0.406	0.095	40.6	40.6	9.5	(30.116 to 51.651)
cg19388050	11	2562647	3.175	0.252	3.183	0.031	0.031	-0.222	0.4875	0.4875	0.826	48.75	48.75	82.6	(37.888 to 59.719)
cg17255148	11	2563802	2.612	0.229	2.442	-0.738	-0.738	5.272	0.232	0.232	3.03E-06	23.2	23.2	0.000303	(14.539 to 33.407)
cg26884837	11	2563897	2.364	0.293	1.9	-1.567	-1.567	11.194	0.0615	0.062	4.22E-15	6.15	6.2	4.22E-13	(2.291 to 12.271)
cg03905757	11	2566193	1.99	0.298	2.029	0.128	0.128	-0.913	0.4495	0.4495	0.366	44.95	44.95	36.6	(34.209 to 55.959)
cg20850984	11	2569120	2.615	0.246	2.574	-0.165	-0.165	1.179	0.435	0.435	0.244	43.5	43.5	24.4	(32.813 to 54.505)
cg17494446	11	2569380	3.195	0.207	2.825	-1.77	-1.77	12.644	0.0415	0.0415	0	4.15	4.15	0	(1.277 to 9.084)
cg23545049	11	2569474	2.573	0.275	2.598	0.088	0.088	-0.63	0.465	0.465	0.532	46.5	46.5	53.2	(35.707 to 57.502)
cg26639864	11	2569788	2.902	0.229	2.768	-0.581	-0.581	4.148	0.282	0.282	0	28.2	28.2	0	(18.824 to 38.836)
cg08419850	11	2569801	3.411	0.176	3.53	0.67	0.669	-4.782	0.253	0.253	1.63E-05	25.3	25.3	0.00163	(16.327 to 35.727)
cg20768429	11	2569832	3.183	0.261	3.493	1.177	1.177	-8.405	0.1225	0.1225	4.62E-11	12.25	12.25	4.62E-09	(6.078 to 20.558)
cg09197492	11	2571399	2.773	0.231	2.844	0.303	0.303	-2.163	0.3815	0.3815	0.035	38.15	38.15	3.5	(27.822 to 49.172)
cg15074690	11	2571484	2.478	0.18	2.855	2.07	2.069	-14.78	0.022	0.022	0	2.2	2.2	0	(0.489 to 5.596)
cg09390414	11	2571525	3.137	0.246	3.175	0.151	0.151	-1.08	0.44	0.44	0.286	44	44	28.6	(33.333 to 55.049)
cg03820779	11	2573972	2.831	0.218	2.906	0.344	0.344	-2.454	0.3665	0.3665	0.018	36.65	36.65	1.8	(26.404 to 47.612)
cg09254350	11	2574026	3.136	0.251	3.561	1.673	1.672	-11.944	0.0505	0.0505	4.44E-16	5.05	5.05	4.44E-14	(1.704 to 10.531)

cg00382572	11	2574042	2.58	0.277	2.597	0.061	0.061	-0.435	0.476	0.476	0.665	47.6	47.6	66.5	(36.742 to 58.558)
cg14947466	11	2583705	2.382	0.156	2.361	-0.133	-0.133	0.953	0.447	0.447	0.345	44.7	44.7	34.5	(33.998 to 55.740)
cg26344859	11	2584629	2.515	0.143	2.462	-0.367	-0.367	2.622	0.3575	0.3575	0.012	35.75	35.75	1.2	(25.599 to 46.717)
cg12424548	11	2591932	1.919	0.242	1.551	-1.502	-1.502	10.728	0.0695	0.07	1.87E-14	6.95	7	1.87E-12	(2.733 to 13.450)
cg08007665	11	2593857	2.135	0.194	2.404	1.371	1.37	-9.789	0.0885	0.0885	4.06E-13	8.85	8.85	4.06E-11	(3.836 to 16.074)
cg26750319	11	2593960	2.736	0.153	2.823	0.565	0.565	-4.037	0.287	0.2875	0	28.7	28.75	0	(19.281 to 39.392)
cg13536051	11	2594099	2.189	0.199	2.407	1.085	1.084	-7.746	0.1415	0.142	4.68E-10	14.15	14.2	4.68E-08	(7.443 to 22.954)
cg20751395	11	2594153	2.607	0.159	2.667	0.37	0.37	-2.642	0.3565	0.3565	0.011	35.65	35.65	1.1	(25.504 to 46.611)
cg13145504	11	2594840	1.294	0.18	1.343	0.272	0.272	-1.94	0.3935	0.3935	0.058	39.35	39.35	5.8	(28.928 to 50.374)
cg24492694	11	2595605	3.002	0.376	3.291	0.761	0.761	-5.438	0.225	0.225	1.70E-06	22.5	22.5	0.00017	(13.961 to 32.637)
cg23049234	11	2595718	2.821	0.183	2.792	-0.16	-0.16	1.141	0.437	0.437	0.26	43.7	43.7	26	(33.014 to 54.716)
cg12223985	11	2595800	2.971	0.211	2.903	-0.318	-0.318	2.271	0.376	0.376	0.028	37.6	37.6	2.8	(27.295 to 48.595)
cg06047778	11	2596300	2.62	0.129	2.15	-3.597	-3.597	25.69	0.0005	0.0005	0	0.05	0.05	0	(0.001 to 0.211)
cg06960356	11	2596416	3.129	0.247	3.231	0.408	0.408	-2.916	0.3425	0.3425	0.005	34.25	34.25	0.5	(24.215 to 45.161)
cg01616349	11	2596468	2.371	0.164	2.393	0.131	0.131	-0.936	0.448	0.448	0.354	44.8	44.8	35.4	(34.088 to 55.834)
cg18865126	11	2596606	2.248	0.183	2.401	0.83	0.83	-5.93	0.205	0.2055	3.00E-07	20.5	20.55	3.00E-05	(12.336 to 30.412)
cg20124933	11	2597564	3.189	0.298	3.331	0.471	0.471	-3.361	0.32	0.32	0.002	32	32	0.2	(22.190 to 42.838)
cg20170839	11	2597721	2.695	0.143	2.496	-1.372	-1.371	9.795	0.088	0.0885	3.98E-13	8.8	8.85	3.98E-11	(3.829 to 16.057)
cg00243281	11	2597838	1.781	0.169	1.996	1.257	1.256	-8.974	0.1075	0.1075	6.44E-12	10.75	10.75	6.44E-10	(5.059 to 18.625)
cg24609402	11	2598247	2.738	0.174	2.835	0.552	0.552	-3.944	0.2915	0.2915	0	29.15	29.15	0	(19.668 to 39.858)
cg06873192	11	2598347	2.207	0.147	2.313	0.709	0.709	-5.064	0.241	0.241	6.22E-06	24.1	24.1	0.000622	(15.284 to 34.384)
cg26411299	11	2598653	2.6	0.194	2.395	-1.045	-1.044	7.46	0.1505	0.151	1.29E-09	15.05	15.1	1.29E-07	(8.103 to 24.050)
cg25223664	11	2600975	1.765	0.16	2.036	1.675	1.675	-11.965	0.05	0.05	4.44E-16	5	5	4.44E-14	(1.690 to 10.486)
cg13612693	11	2602171	2.398	0.138	2.113	-2.052	-2.051	14.651	0.023	0.023	0	2.3	2.3	0	(0.520 to 5.771)
cg16412789	11	2602926	3.334	0.205	3.356	0.104	0.104	-0.746	0.4585	0.4585	0.459	45.85	45.85	45.9	(35.089 to 56.867)
cg22195512	11	2602980	2.082	0.124	2.085	0.026	0.026	-0.189	0.4895	0.4895	0.851	48.95	48.95	85.1	(38.062 to 59.894)
cg06827779	11	2603011	2.533	0.185	2.591	0.312	0.312	-2.229	0.378	0.378	0.03	37.8	37.8	3	(27.498 to 48.818)
cg04211344	11	2603221	2.815	0.167	2.894	0.467	0.467	-3.333	0.3215	0.3215	0.002	32.15	32.15	0.2	(22.316 to 42.984)
cg06264108	11	2603429	3.276	0.183	3.071	-1.107	-1.107	7.908	0.137	0.137	2.64E-10	13.7	13.7	2.64E-08	(7.089 to 22.350)
cg23040992	11	2603590	2.654	0.19	2.779	0.649	0.649	-4.634	0.2595	0.26	2.68E-05	25.95	26	0.00268	(16.890 to 36.440)
cg19030519	11	2604365	1.964	0.124	2.101	1.094	1.094	-7.812	0.1395	0.14	3.71E-10	13.95	14	3.71E-08	(7.298 to 22.707)

cg25175863	11	2604391	1.329	0.117	1.557	1.943	1.942	-13.875	0.029	0.029	0	2.9	2.9	0	(0.745 to 6.914)
cg20921686	11	2604499	2.549	0.196	2.136	-2.094	-2.094	14.957	0.0205	0.021	0	2.05	2.1	0	(0.449 to 5.364)
cg12263990	11	2606365	2.733	0.158	3.003	1.696	1.696	-12.111	0.048	0.048	2.22E-16	4.8	4.8	2.22E-14	(1.592 to 10.171)
cg08861541	11	2610149	3.12	0.196	3.025	-0.48	-0.479	3.424	0.317	0.317	0.001	31.7	31.7	0.1	(21.908 to 42.510)
cg16222896	11	2610580	2.883	0.209	3.145	1.242	1.242	-8.868	0.11	0.11	9.28E-12	11	11	9.28E-10	(5.239 to 18.976)
cg20828897	11	2610595	2.656	0.228	2.862	0.896	0.896	-6.399	0.1875	0.1875	5.65E-08	18.75	18.75	5.65E-06	(10.907 to 28.369)
cg26728427	11	2610741	2.681	0.161	2.7	0.118	0.118	-0.839	0.4535	0.4535	0.405	45.35	45.35	40.5	(34.596 to 56.359)
cg09315662	11	2612781	2.277	0.141	2.27	-0.051	-0.051	0.366	0.4795	0.4795	0.716	47.95	47.95	71.6	(37.111 to 58.933)
cg21695475	11	2612825	2.45	0.18	2.536	0.476	0.475	-3.396	0.3185	0.3185	0.001	31.85	31.85	0.1	(22.035 to 42.658)
cg13742956	11	2612876	2.34	0.192	2.351	0.059	0.058	-0.418	0.477	0.477	0.678	47.7	47.7	67.8	(36.835 to 58.653)
cg19323901	11	2614062	2.269	0.135	2.361	0.676	0.675	-4.825	0.251	0.2515	1.41E-05	25.1	25.15	0.00141	(16.165 to 35.521)
cg14089425	11	2616834	2.217	0.445	2.379	0.36	0.359	-2.568	0.3605	0.3605	0.013	36.05	36.05	1.3	(25.860 to 47.008)
cg25786675	11	2621273	3.082	0.206	3.441	1.724	1.723	-12.31	0.0455	0.0455	0	4.55	4.55	0	(1.468 to 9.754)
cg07040680	11	2621365	1.847	0.189	2.099	1.32	1.32	-9.427	0.0965	0.0965	1.37E-12	9.65	9.65	1.37E-10	(4.347 to 17.174)
cg07003739	11	2640527	1.609	0.158	1.517	-0.578	-0.578	4.126	0.283	0.283	0	28.3	28.3	0	(18.911 to 38.943)
cg02021157	11	2644078	2.209	0.176	2.512	1.712	1.711	-12.225	0.0465	0.0465	2.22E-16	4.65	4.65	2.22E-14	(1.520 to 9.932)
cg03660952	11	2644418	0.333	0.189	0.385	0.273	0.273	-1.95	0.393	0.393	0.057	39.3	39.3	5.7	(28.881 to 50.322)
cg19095714	11	2645098	3.246	0.365	3.2	-0.125	-0.125	0.89	0.4505	0.4505	0.378	45.05	45.05	37.8	(34.329 to 56.083)
cg08249467	11	2646113	2.71	0.257	3.247	2.07	2.069	-14.78	0.022	0.022	0	2.2	2.2	0	(0.489 to 5.596)
cg24932449	11	2672613	2.494	0.221	2.753	1.162	1.162	-8.302	0.1255	0.1255	6.63E-11	12.55	12.55	6.63E-09	(6.279 to 20.924)
cg18477587	11	2672740	2.356	0.191	2.516	0.83	0.829	-5.924	0.2055	0.2055	3.06E-07	20.55	20.55	3.06E-05	(12.355 to 30.439)
cg07556018	11	2677341	-0.604	0.237	-0.437	0.697	0.697	-4.977	0.2445	0.2445	8.38E-06	24.45	24.45	0.000838	(15.600 to 34.795)
cg13428066	11	2677768	-0.923	0.216	-1.126	-0.934	-0.934	6.668	0.1775	0.1775	2.17E-08	17.75	17.75	2.17E-06	(10.140 to 27.234)
cg05753930	11	2678450	2.639	0.248	2.832	0.771	0.77	-5.503	0.2225	0.2225	1.35E-06	22.25	22.25	0.000135	(13.740 to 32.340)
cg18106482	11	2680314	3.188	0.225	3.676	2.152	2.152	-15.369	0.018	0.018	0	1.8	1.8	0	(0.367 to 4.854)
cg06585606	11	2680854	3.109	0.245	2.698	-1.661	-1.66	11.859	0.0515	0.0515	4.44E-16	5.15	5.15	4.44E-14	(1.764 to 10.720)
cg06914593	11	2684842	2.671	0.137	2.876	1.481	1.481	-10.58	0.0725	0.0725	3.00E-14	7.25	7.25	3.00E-12	(2.888 to 13.843)
cg26190483	11	2689695	3.44	0.2	3.227	-1.053	-1.053	7.519	0.149	0.149	1.05E-09	14.9	14.9	1.05E-07	(7.963 to 23.820)
cg22239603	11	2690304	2.748	0.168	2.618	-0.767	-0.766	5.474	0.2235	0.2235	1.49E-06	22.35	22.35	0.000149	(13.838 to 32.472)
cg13203297	11	2691249	2.94	0.196	3.224	1.433	1.433	-10.234	0.079	0.079	9.28E-14	7.9	7.9	9.28E-12	(3.276 to 14.791)
cg15422307	11	2693503	1.454	0.211	2.104	3.047	3.047	-21.762	0.002	0.002	0	0.2	0.2	0	(0.009 to 0.805)

cg20689362	11	2694005	2.273	0.117	2.21	-0.532	-0.531	3.796	0.2985	0.299	0	29.85	29.9	0	(20.291 to 40.604)
cg13880539	11	2696589	2.902	0.234	3.189	1.21	1.21	-8.644	0.116	0.116	2.01E-11	11.6	11.6	2.01E-09	(5.633 to 19.731)
cg17402907	11	2697109	2.786	0.386	2.759	-0.068	-0.068	0.485	0.473	0.473	0.63	47.3	47.3	63	(36.477 to 58.289)
cg20402210	11	2697527	1.944	0.129	2.312	2.827	2.826	-20.186	0.0035	0.0035	0	0.35	0.35	0	(0.025 to 1.309)
cg06294475	11	2698623	-0.912	0.336	-2.111	-3.532	-3.531	25.224	0.0005	0.0005	0	0.05	0.05	0	(0.001 to 0.250)
cg03411938	11	2700003	0.421	0.163	0.43	0.054	0.054	-0.382	0.479	0.479	0.704	47.9	47.9	70.4	(37.026 to 58.847)
cg17128405	11	2700832	2.752	0.302	2.751	-0.003	-0.003	0.021	0.499	0.499	0.984	49.9	49.9	98.4	(38.971 to 60.807)
cg18759359	11	2700935	2.865	0.2	3.006	0.701	0.701	-5.006	0.2435	0.2435	7.59E-06	24.35	24.35	0.000759	(15.495 to 34.658)
cg20406758	11	2703128	1.914	0.228	1.744	-0.737	-0.737	5.265	0.232	0.2325	3.09E-06	23.2	23.25	0.000309	(14.561 to 33.437)
cg07683142	11	2704514	2.351	0.193	2.021	-1.69	-1.689	12.067	0.0485	0.049	2.22E-16	4.85	4.9	2.22E-14	(1.621 to 10.266)
cg01693193	11	2705153	2.052	0.181	1.977	-0.406	-0.406	2.898	0.3435	0.3435	0.006	34.35	34.35	0.6	(24.301 to 45.258)
cg13444966	11	2705357	2.795	0.259	3.35	2.118	2.118	-15.125	0.0195	0.0195	0	1.95	1.95	0	(0.414 to 5.151)
cg15861694	11	2706745	2.237	0.128	2.24	0.026	0.026	-0.184	0.49	0.49	0.855	49	49	85.5	(38.091 to 59.923)
cg08160246	11	2709914	1.371	0.12	1.345	-0.211	-0.211	1.509	0.417	0.417	0.138	41.7	41.7	13.8	(31.113 to 52.713)
cg02180189	11	2710486	1.305	0.21	1.793	2.305	2.305	-16.461	0.0125	0.013	0	1.25	1.3	0	(0.211 to 3.690)
cg11342234	11	2710838	0.69	0.186	0.281	-2.183	-2.182	15.588	0.017	0.017	0	1.7	1.7	0	(0.329 to 4.599)
cg07072281	11	2713432	2.299	0.145	2.036	-1.795	-1.795	12.822	0.0395	0.0395	0	3.95	3.95	0	(1.184 to 8.742)
cg19672982	11	2715154	2.702	0.232	2.819	0.498	0.498	-3.559	0.31	0.3105	0.001	31	31.05	0.1	(21.315 to 41.816)
cg13577072	11	2715286	1.415	0.159	1.066	-2.177	-2.176	15.546	0.017	0.017	0	1.7	1.7	0	(0.336 to 4.648)
cg25204743	11	2715837	0.657	0.177	0.144	-2.871	-2.87	20.502	0.003	0.003	0	0.3	0.3	0	(0.021 to 1.190)
cg11875582	11	2718307	2.284	0.326	1.42	-2.629	-2.628	18.774	0.0055	0.0055	0	0.55	0.55	0	(0.059 to 1.975)
cg27119222	11	2720229	0.316	0.145	-1.994	-15.738	-15.735	112.394	0	0	0	0	0	0	(1.43E-78 to 5.27E-35)
cg00000924	11	2720463	0.099	0.108	-1.861	-17.971	-17.968	128.34	0	0	0	0	0	0	(2.37E-102 to 1.20E-45)
cg11297256	11	2720810	0.468	0.157	-2.091	-16.142	-16.139	115.276	0	0	0	0	0	0	(1.23E-82 to 7.97E-37)
cg14392746	11	2721207	-0.158	0.119	-2.748	-21.639	-21.634	154.531	0	0	0	0	0	0	(3.23E-148 to 3.66E-66)
cg12077660	11	2721243	0.097	0.115	-0.74	-7.229	-7.228	51.626	1.47E-09	1.65E-09	0	1.47E-07	1.65E-07	0	(1.21E-16 to 3.48E-07)
cg03401726	11	2721248	0.208	0.118	-0.916	-9.414	-9.412	67.226	7.2E-13	8.85E-13	0	7.2E-11	8.85E-11	0	(3.26E-28 to 2.08E-12)
cg16739686	11	2721336	0.133	0.155	-2.459	-16.579	-16.576	118.401	0	0	0	0	0	0	(3.66E-87 to 7.51E-39)
cg08359167	11	2721351	0.07	0.122	-0.776	-6.853	-6.851	48.938	5.6E-09	6.2E-09	0	5.6E-07	6.2E-07	0	(5.98E-15 to 2.04E-06)
cg08446215	11	2721366	0.164	0.143	-2.543	-18.738	-18.735	133.819	0	0	0	0	0	0	(3.02E-111 to 1.25E-49)
cg26104781	11	2721383	0.22	0.152	-2.519	-17.878	-17.874	127.674	0	0	0	0	0	0	(2.68E-101 to 3.54E-45)

cg02219360	11	2721409	-0.003	0.143	-1.837	-12.734	-12.731	90.939	0	0	0	0	0	0	(1.60E-51 to 6.99E-23)
cg20699737	11	2721428	0.054	0.149	-2.085	-14.253	-14.251	101.79	0	0	0	0	0	0	(1.71E-64 to 1.07E-28)
cg26547719	11	2721437	-0.015	0.126	-1.528	-11.877	-11.875	84.82	2.22E-16	3.33E-16	0	2.22E-14	3.33E-14	0	(7.61E-45 to 6.93E-20)
cg07595203	11	2721480	-0.474	0.124	-2.51	-16.212	-16.209	115.776	0	0	0	0	0	0	(2.36E-83 to 3.81E-37)
cg09518720	11	2721591	0.392	0.216	-1.361	-8.04	-8.038	57.414	8.3E-11	9.65E-11	0	8.3E-09	9.65E-09	0	(1.37E-20 to 5.71E-09)
cg14958441	11	2721610	-0.374	0.084	-1.596	-14.456	-14.453	103.238	0	0	0	0	0	0	(2.46E-66 to 1.60E-29)
cg27323091	11	2721619	0.058	0.1	-0.959	-10.08	-10.078	71.985	7.75E-14	9.8E-14	0	7.75E-12	9.8E-12	0	(2.45E-32 to 2.89E-14)
cg01873334	11	2721632	0.558	0.141	-1.295	-12.986	-12.983	92.737	0	0	0	0	0	0	(1.43E-53 to 8.42E-24)
cg05816130	11	2721799	0.125	0.1	-1.513	-16.207	-16.204	115.743	0	0	0	0	0	0	(2.63E-83 to 4.00E-37)
cg14243741	11	2721817	0.379	0.13	-1.677	-15.674	-15.671	111.937	0	0	0	0	0	0	(6.15E-78 to 1.01E-34)
cg21137515	11	2721857	0.06	0.097	-1.824	-19.241	-19.238	137.412	0	0	0	0	0	0	(2.79E-117 to 2.50E-52)
cg05740879	11	2721866	0.174	0.104	-1.716	-17.913	-17.91	127.928	0	0	0	0	0	0	(1.06E-101 to 2.34E-45)
cg01893176	11	2721952	-0.383	0.086	-2.128	-20.029	-20.026	143.039	0	0	0	0	0	0	(4.73E-127 to 1.07E-56)
cg11993252	11	2721961	0.26	0.119	-0.931	-9.932	-9.93	70.926	1.27E-13	1.6E-13	0	1.27E-11	1.6E-11	0	(2.14E-31 to 7.67E-14)
cg26094482	11	2722073	-0.277	0.118	-2.047	-14.832	-14.829	105.919	0	0	0	0	0	0	(8.24E-70 to 4.43E-31)
cg26908876	11	2722076	-0.266	0.129	-2.882	-20.123	-20.119	143.704	0	0	0	0	0	0	(3.14E-128 to 3.17E-57)
cg03422070	11	2722082	0.19	0.116	-2.312	-21.423	-21.419	152.994	0	0	0	0	0	0	(2.72E-145 to 7.45E-65)
cg15651941	11	2722084	-0.071	0.135	-1.965	-13.841	-13.839	98.847	0	0	0	0	0	0	(7.84E-61 to 4.67E-27)
cg25306939	11	2722086	0.036	0.107	-2.316	-21.735	-21.73	155.215	0	0	0	0	0	0	(1.57E-149 to 9.49E-67)
cg06288089	11	2722119	-0.106	0.089	-2.302	-24.355	-24.351	173.933	0	0	0	0	0	0	(1.19E-187 to 8.64E-84)
cg02798157	11	2722195	-0.097	0.113	-1.931	-16.122	-16.118	115.132	0	0	0	0	0	0	(1.97E-82 to 9.85E-37)
cg27604721	11	2722258	0.406	0.132	-1.616	-15.18	-15.177	108.405	0	0	0	0	0	0	(4.10E-73 to 1.47E-32)
cg12433386	11	2722340	1.167	0.217	-0.602	-8.079	-8.078	57.698	7.25E-11	8.4E-11	0	7.25E-09	8.4E-09	0	(8.59E-21 to 4.62E-09)
cg07123182	11	2722391	2.992	0.229	2.803	-0.822	-0.822	5.873	0.2075	0.2075	3.68E-07	20.75	20.75	3.68E-05	(12.520 to 30.668)
cg16556677	11	2722401	1.768	0.244	0.846	-3.74	-3.739	26.708	0	0	0	0	0	0	(0.000 to 0.145)
cg26963277	11	2722407	2.27	0.223	1.761	-2.258	-2.258	16.126	0.014	0.0145	0	1.4	1.45	0	(0.251 to 4.020)
cg11272547	11	2722440	2.5	0.164	2.277	-1.344	-1.344	9.601	0.0925	0.0925	7.63E-13	9.25	9.25	7.63E-11	(4.095 to 16.639)
cg03654058	11	2722539	2.232	0.274	1.853	-1.369	-1.368	9.775	0.0885	0.089	4.26E-13	8.85	8.9	4.26E-11	(3.856 to 16.117)
cg06809295	11	2722625	2.649	0.158	2.586	-0.4	-0.4	2.855	0.3455	0.3455	0.006	34.55	34.55	0.6	(24.502 to 45.486)
cg19678029	11	2722663	2.698	0.19	2.735	0.194	0.194	-1.384	0.4235	0.4235	0.173	42.35	42.35	17.3	(31.752 to 53.391)
cg04762676	11	2722713	2.408	0.205	2.276	-0.637	-0.637	4.552	0.2635	0.2635	3.53E-05	26.35	26.35	0.00353	(17.209 to 36.842)

cg03512098	11	2730129	3.434	0.196	3.366	-0.346	-0.346	2.469	0.3655	0.3655	0.017	36.55	36.55	1.7	(26.332 to 47.533)
cg19764489	11	2730616	1.695	0.233	1.054	-2.723	-2.722	19.446	0.0045	0.0045	0	0.45	0.45	0	(0.040 to 1.629)
cg26438846	11	2731392	2.601	0.3	2.756	0.511	0.511	-3.647	0.306	0.306	0.001	30.6	30.6	0.1	(20.932 to 41.365)
cg07592519	11	2735101	3.136	0.257	3.142	0.025	0.025	-0.18	0.49	0.49	0.858	49	49	85.8	(38.113 to 59.945)
cg17227621	11	2741033	2.846	0.157	3.122	1.744	1.744	-12.454	0.0435	0.044	0	4.35	4.4	0	(1.383 to 9.462)
cg15782852	11	2742507	2.626	0.182	2.288	-1.846	-1.846	13.185	0.0355	0.0355	0	3.55	3.55	0	(1.012 to 8.073)
cg16811455	11	2742553	1.708	0.158	2.112	2.534	2.533	-18.096	0.0075	0.0075	0	0.75	0.75	0	(0.087 to 2.388)
cg17305275	11	2746675	2.563	0.281	2.877	1.111	1.11	-7.931	0.136	0.136	2.44E-10	13.6	13.6	2.44E-08	(7.041 to 22.267)
cg24725201	11	2753818	2.931	0.174	3.161	1.305	1.305	-9.322	0.099	0.099	1.97E-12	9.9	9.9	1.97E-10	(4.506 to 17.506)
cg15686157	11	2753885	1.798	0.178	1.672	-0.701	-0.701	5.009	0.243	0.2435	7.50E-06	24.3	24.35	0.00075	(15.482 to 34.641)
cg04907802	11	2753950	3.026	0.21	2.904	-0.579	-0.579	4.137	0.2825	0.2825	0	28.25	28.25	0	(18.867 to 38.889)
cg04894537	11	2763171	-1.115	0.175	-2.52	-7.941	-7.939	56.707	1.18E-10	1.36E-10	0	1.18E-08	1.36E-08	0	(4.38E-20 to 9.64E-09)
cg01917852	11	2772759	2.107	0.251	2.646	2.126	2.125	-15.18	0.0195	0.0195	0	1.95	1.95	0	(0.403 to 5.083)
cg16434897	11	2772799	1.988	0.212	2.423	2.032	2.032	-14.513	0.024	0.024	0	2.4	2.4	0	(0.555 to 5.963)
cg13579167	11	2772852	3.049	0.195	3.439	1.974	1.974	-14.098	0.027	0.027	0	2.7	2.7	0	(0.673 to 6.568)
cg11585942	11	2774756	2.385	0.149	2.403	0.119	0.119	-0.85	0.453	0.453	0.399	45.3	45.3	39.9	(34.537 to 56.299)
cg25356736	11	2775022	0.452	0.227	0.203	-1.087	-1.086	7.759	0.1415	0.1415	4.47E-10	14.15	14.15	4.47E-08	(7.415 to 22.906)
cg19975301	11	2779993	2.729	0.177	2.817	0.492	0.492	-3.515	0.3125	0.3125	0.001	31.25	31.25	0.1	(21.507 to 42.041)
cg05624382	11	2780050	2.85	0.173	2.804	-0.263	-0.263	1.878	0.397	0.397	0.066	39.7	39.7	6.6	(29.243 to 50.713)
cg11249934	11	2782519	1.322	0.188	0.808	-2.711	-2.71	19.36	0.0045	0.0045	0	0.45	0.45	0	(0.042 to 1.670)
cg06889525	11	2782615	2.5	0.147	2.713	1.435	1.435	-10.251	0.079	0.079	8.79E-14	7.9	7.9	8.79E-12	(3.256 to 14.742)
cg13893637	11	2783554	2.015	0.185	2.224	1.12	1.119	-7.996	0.134	0.1345	1.94E-10	13.4	13.45	1.94E-08	(6.902 to 22.028)
cg15467785	11	2783777	2.498	0.221	2.906	1.826	1.826	-13.042	0.037	0.037	0	3.7	3.7	0	(1.077 to 8.332)
cg15910264	11	2783880	2.388	0.169	2.305	-0.484	-0.484	3.456	0.3155	0.3155	0.001	31.55	31.55	0.1	(21.767 to 42.345)
cg06850651	11	2785580	2.198	0.289	2.582	1.317	1.316	-9.402	0.097	0.097	1.49E-12	9.7	9.7	1.49E-10	(4.384 to 17.253)
cg23750514	11	2790418	1.667	0.275	2.169	1.805	1.804	-12.889	0.0385	0.0385	0	3.85	3.85	0	(1.150 to 8.615)
cg05438727	11	2800047	-1.034	0.176	-1.947	-5.136	-5.135	36.676	2.43E-06	2.55E-06	0	0.000243	0.000255	0	(2.42E-08 to 0.002)
cg12427469	11	2809574	2.667	0.147	2.688	0.148	0.148	-1.057	0.4415	0.4415	0.296	44.15	44.15	29.6	(33.453 to 55.174)
cg06233503	11	2812674	-1.582	0.194	-1.411	0.872	0.872	-6.227	0.1935	0.194	1.04E-07	19.35	19.4	1.04E-05	(11.419 to 29.112)
cg23903244	11	2812987	-1.954	0.5	-1.898	0.112	0.112	-0.802	0.4555	0.4555	0.426	45.55	45.55	42.6	(34.791 to 56.560)
cg19728223	11	2813121	-2.157	0.409	-1.814	0.829	0.828	-5.918	0.2055	0.206	3.13E-07	20.55	20.6	3.13E-05	(12.376 to 30.468)

cg17820828	11	2813298	0.222	0.393	0.53	0.776	0.776	-5.543	0.2205	0.221	1.17E-06	22.05	22.1	0.000117	(13.603 to 32.155)
cg21752270	11	2813528	1.469	0.168	1.743	1.619	1.619	-11.563	0.056	0.056	1.33E-15	5.6	5.6	1.33E-13	(1.984 to 11.391)
cg12901038	11	2815078	2.705	0.133	2.727	0.164	0.164	-1.168	0.4355	0.4355	0.248	43.55	43.55	24.8	(32.870 to 54.565)
cg14384214	11	2817181	2.593	0.261	3.075	1.826	1.825	-13.039	0.037	0.037	0	3.7	3.7	0	(1.079 to 8.337)
cg24478595	11	2817646	2.896	0.15	2.895	-0.007	-0.007	0.05	0.497	0.497	0.961	49.7	49.7	96.1	(38.814 to 60.650)
cg16778148	11	2817922	2.572	0.173	2.737	0.944	0.944	-6.742	0.175	0.175	1.66E-08	17.5	17.5	1.66E-06	(9.937 to 26.927)
cg26879282	11	2817991	3.007	0.25	2.854	-0.604	-0.604	4.316	0.274	0.2745	7.69E-05	27.4	27.45	0.00769	(18.141 to 37.999)
cg01689159	11	2818128	2.22	0.118	2.462	2.024	2.023	-14.451	0.024	0.0245	0	2.4	2.45	0	(0.571 to 6.050)
cg00325528	11	2819073	2.283	0.122	2.393	0.891	0.891	-6.364	0.1885	0.1885	6.41E-08	18.85	18.85	6.41E-06	(11.009 to 28.519)
cg16419342	11	2821200	2.808	0.188	3.161	1.857	1.857	-13.263	0.0345	0.0345	0	3.45	3.45	0	(0.978 to 7.935)
cg04008557	11	2826328	2.349	0.117	2.224	-1.056	-1.056	7.54	0.148	0.148	9.70E-10	14.8	14.8	9.70E-08	(7.913 to 23.737)
cg27050114	11	2826795	3.169	0.325	2.806	-1.109	-1.108	7.917	0.1365	0.1365	2.56E-10	13.65	13.65	2.56E-08	(7.071 to 22.319)
cg12851635	11	2826951	3.143	0.203	3.405	1.277	1.277	-9.121	0.104	0.104	3.89E-12	10.4	10.4	3.89E-10	(4.819 to 18.146)
cg13253153	11	2826988	2.84	0.282	2.796	-0.152	-0.152	1.085	0.44	0.44	0.283	44	44	28.3	(33.305 to 55.020)
cg26671281	11	2828214	1.623	0.16	1.201	-2.608	-2.607	18.622	0.006	0.006	0	0.6	0.6	0	(0.064 to 2.062)
cg04759114	11	2828274	2.05	0.161	1.714	-2.059	-2.058	14.703	0.0225	0.0225	0	2.25	2.25	0	(0.507 to 5.700)
cg16609872	11	2828364	1.544	0.165	1.546	0.013	0.013	-0.091	0.495	0.495	0.928	49.5	49.5	92.8	(38.592 to 60.427)
cg25173341	11	2828525	1.927	1.068	-0.564	-2.309	-2.309	16.492	0.0125	0.0125	0	1.25	1.25	0	(0.208 to 3.661)
cg16324958	11	2828568	2.776	0.27	3.22	1.629	1.628	-11.632	0.055	0.055	1.11E-15	5.5	5.5	1.11E-13	(1.931 to 11.232)
cg07979757	11	2828652	1.905	0.293	1.448	-1.544	-1.544	11.029	0.0645	0.0645	7.11E-15	6.45	6.45	7.11E-13	(2.440 to 12.679)
cg19923326	11	2828778	1.859	0.2	1.46	-1.974	-1.973	14.094	0.027	0.027	0	2.7	2.7	0	(0.674 to 6.574)
cg04635504	11	2829241	2.294	0.151	2.073	-1.448	-1.447	10.338	0.077	0.077	6.59E-14	7.7	7.7	6.59E-12	(3.155 to 14.500)
cg03571121	11	2832861	1.953	0.151	2.039	0.567	0.567	-4.047	0.2865	0.287	0	28.65	28.7	0	(19.236 to 39.337)
cg03155200	11	2833259	-0.046	0.334	-1.513	-4.353	-4.352	31.084	3.41E-05	3.52E-05	0	0.00341	0.003515	0	(5.67E-06 to 0.025)
cg03197835	11	2835996	2.087	0.259	2.583	1.901	1.901	-13.578	0.0315	0.0315	0	3.15	3.15	0	(0.851 to 7.396)
cg26958174	11	2840699	2.611	0.161	2.678	0.418	0.417	-2.982	0.339	0.339	0.004	33.9	33.9	0.4	(23.910 to 44.815)
cg19852660	11	2846681	0.888	0.161	0.768	-0.736	-0.736	5.257	0.2325	0.2325	3.18E-06	23.25	23.25	0.000318	(14.591 to 33.475)
cg13859639	11	2846716	-0.39	0.143	-0.649	-1.802	-1.802	12.869	0.039	0.039	0	3.9	3.9	0	(1.160 to 8.653)
cg17416793	11	2846932	-0.261	0.205	-0.365	-0.502	-0.502	3.587	0.309	0.309	0.001	30.9	30.9	0.1	(21.192 to 41.671)
cg17333973	11	2847019	-0.529	0.137	-0.7	-1.247	-1.247	8.905	0.109	0.1095	8.18E-12	10.9	10.95	8.18E-10	(5.175 to 18.853)
cg06485603	11	2847258	-2.336	0.282	-3.662	-4.652	-4.651	33.225	1.26E-05	1.31E-05	0	0.00126	0.001305	0	(8.24E-07 to 0.010)

cg12141659	11	2847462	-1.508	0.208	-3.003	-7.097	-7.095	50.681	2.35E-09	2.63E-09	0	2.35E-07	2.63E-07	0	(4.89E-16 to 6.55E-07)
cg10151367	11	2847642	-2.063	0.22	-3.079	-4.57	-4.569	32.637	1.66E-05	1.72E-05	0	0.00166	0.00172	0	(1.45E-06 to 0.013)
cg21130221	11	2848310	-0.405	0.24	-0.802	-1.636	-1.635	11.681	0.054	0.0545	8.88E-16	5.4	5.45	8.88E-14	(1.893 to 11.119)
cg18272633	11	2848492	0.981	0.194	0.88	-0.519	-0.519	3.704	0.303	0.303	0.001	30.3	30.3	0.1	(20.688 to 41.076)

Supplementary Table 9. Performance of CH-test in detecting hypomethylation at the cardinal loci in an MLMD sample. The table presents 450k DNA methylation data across *KCNQ1* region in BWS-MLMD4 sample. The point estimate represents the estimated percentage of the control population that would be expected to obtain lower score than the case and 95% confidence interval of point estimates were calculated from the noncentrality parameter from a noncentral t-distribution along with the point and interval estimates of the effect size. Here, ILMNID = Illumina Infinium HumanMethylation450 array probe ID, Chr = chromosome, Map information = genomic location (hg19), Mean = Control mean, and SD = control standard deviation, adjusted-p = Adjusted p-value using false discovery rate method, z_{cc} = point estimate of the effect size.

			Control group			Significance test		Estimated percentage of the control population obtaining a lower score than the case			Estimated effect size	
ILMNID	Chr	Map Information	Mean	SD	Case score	t-value	p-value	Point	(95%CI)	Adjusted p	z_{cc}	(95%CI)
cg00000924	11	2720463	0.104	0.108	-0.93	-9.52	0	0	(0.000 to 0.000)	0	-9.615	(-11.490 to -7.642)
cg00243281	11	2597838	1.784	0.169	1.429	-2.08	0.021	2.1	(0.472 to 5.499)	0.239	-2.101	(-2.596 to -1.598)
cg00283576	11	2465351	-2.991	0.158	-2.769	1.388	0.086	8.6	(3.676 to 15.716)	0.377	1.402	(1.006 to 1.790)
cg00325528	11	2819073	2.286	0.122	2.231	-0.446	0.329	32.9	(22.982 to 43.753)	0.483	-0.45	(-0.739 to -0.157)
cg00382572	11	2574042	2.583	0.277	2.879	1.059	0.147	14.7	(7.867 to 23.662)	0.428	1.069	(0.717 to 1.414)
cg01178971	11	2464851	1.297	0.278	1.042	-0.907	0.184	18.4	(10.676 to 28.031)	0.446	-0.916	(-1.244 to -0.582)
cg01298120	11	2499341	3.17	0.182	3.305	0.739	0.232	23.2	(14.526 to 33.389)	0.462	0.746	(0.429 to 1.057)
cg01616349	11	2596468	2.374	0.164	2.308	-0.402	0.345	34.5	(24.434 to 45.409)	0.485	-0.406	(-0.692 to -0.115)
cg01689159	11	2818128	2.223	0.118	2.337	0.956	0.172	17.2	(9.714 to 26.589)	0.44	0.965	(0.625 to 1.298)
cg01693193	11	2705153	2.055	0.181	2.29	1.285	0.102	10.2	(4.725 to 17.956)	0.394	1.298	(0.917 to 1.672)

cg01734338	11	2491231	1.892	0.247	1.935	0.173	0.432	43.2	(32.535 to 54.215)	0.495	0.174	(-0.106 to 0.453)
cg01873334	11	2721632	0.562	0.141	-0.294	-6.01	0	0	(0.000 to 0.000)	0	-6.069	(-7.272 to -4.837)
cg01893176	11	2721952	-0.375	0.086	-1.249	-10.08	0	0	(0.000 to 0.000)	0	-10.18	(-12.162 to -8.093)
cg01917852	11	2772759	2.11	0.251	2.127	0.068	0.473	47.3	(36.482 to 58.294)	0.498	0.068	(-0.209 to 0.346)
cg02021157	11	2644078	2.212	0.176	2.281	0.389	0.349	34.9	(24.860 to 45.889)	0.486	0.393	(0.103 to 0.679)
cg02097203	11	2548282	-2.99	0.23	-3.03	-0.171	0.432	43.2	(32.582 to 54.263)	0.495	-0.173	(-0.451 to 0.107)
cg02180189	11	2710486	1.308	0.209	0.861	-2.114	0.02	2	(0.419 to 5.185)	0.232	-2.135	(-2.636 to -1.627)
cg02219360	11	2721409	0.003	0.142	-1.073	-7.507	0	0	(0.000 to 0.000)	0	-7.581	(-9.069 to -6.016)
cg02258534	11	2555497	0.441	0.184	0.856	2.229	0.015	1.5	(0.280 to 4.239)	0.209	2.251	(1.724 to 2.771)
cg02335306	11	2559880	2.907	0.183	2.982	0.407	0.343	34.3	(24.270 to 45.223)	0.485	0.411	(0.120 to 0.698)
cg02798157	11	2722195	-0.09	0.112	-0.959	-7.672	0	0	(0.000 to 0.000)	0	-7.748	(-9.267 to -6.150)
cg03030994	11	2562310	2.35	0.176	2.602	1.421	0.081	8.1	(3.378 to 15.029)	0.371	1.435	(1.035 to 1.828)
cg03118430	11	2482233	3.116	0.235	3.126	0.041	0.484	48.4	(37.500 to 59.327)	0.499	0.042	(-0.236 to 0.319)
cg03155200	11	2833259	-0.039	0.332	0.47	1.516	0.068	6.8	(2.632 to 13.189)	0.353	1.531	(1.118 to 1.938)
cg03170016	11	2482142	1.385	0.134	1.034	-2.588	0.006	0.6	(0.070 to 2.145)	0.142	-2.614	(-3.196 to -2.025)
cg03197835	11	2835996	2.09	0.259	2.358	1.026	0.155	15.5	(8.417 to 24.558)	0.432	1.036	(0.688 to 1.378)
cg03365111	11	2481857	2.891	0.195	2.754	-0.694	0.245	24.5	(15.671 to 34.885)	0.466	-0.701	(-1.008 to -0.388)
cg03371125	11	2464845	2.297	0.151	2.374	0.504	0.308	30.8	(21.137 to 41.606)	0.479	0.509	(0.212 to 0.802)
cg03401726	11	2721248	0.214	0.118	-0.578	-6.645	0	0	(0.000 to 0.000)	0	-6.711	(-8.034 to -5.321)
cg03411938	11	2700003	0.426	0.163	0.62	1.181	0.122	12.2	(6.028 to 20.467)	0.411	1.192	(0.825 to 1.552)
cg03422070	11	2722082	0.195	0.115	-1.1	-11.128	0	0	(0.000 to 0.000)	0	-11.239	(-13.424 to -8.939)
cg03512098	11	2730129	3.436	0.196	3.262	-0.882	0.191	19.1	(11.207 to 28.806)	0.448	-0.891	(-1.216 to -0.559)
cg03571121	11	2832861	1.956	0.151	2.082	0.826	0.206	20.6	(12.428 to 30.541)	0.454	0.835	(0.509 to 1.154)
cg03600094	11	2481475	1.998	0.222	2.234	1.052	0.149	14.9	(7.974 to 23.837)	0.429	1.063	(0.712 to 1.407)
cg03654058	11	2722539	2.236	0.274	2.108	-0.46	0.324	32.4	(22.542 to 43.246)	0.482	-0.464	(-0.754 to -0.170)
cg03660952	11	2644418	0.338	0.189	0.293	-0.234	0.408	40.8	(30.296 to 51.844)	0.492	-0.236	(-0.516 to 0.046)
cg03820779	11	2573972	2.834	0.217	2.757	-0.348	0.365	36.5	(26.264 to 47.458)	0.488	-0.351	(-0.635 to -0.064)
cg03905757	11	2566193	1.994	0.298	1.747	-0.819	0.208	20.8	(12.602 to 30.782)	0.455	-0.827	(-1.145 to -0.502)
cg04008557	11	2826328	2.352	0.117	2.367	0.129	0.449	44.9	(34.157 to 55.905)	0.496	0.13	(-0.149 to 0.408)
cg04055211	11	2459151	2.363	0.22	2.526	0.738	0.232	23.2	(14.555 to 33.428)	0.462	0.745	(0.428 to 1.056)
cg04104132	11	2465440	-1.672	0.162	-1.769	-0.592	0.278	27.8	(18.505 to 38.447)	0.474	-0.598	(-0.896 to -0.294)

cg04204548	11	2465887	-3.118	0.218	-3.869	-3.414	0.001	0.1	(0.001 to 0.336)	0.044	-3.448	(-4.180 to -2.710)
cg04211344	11	2603221	2.818	0.167	2.815	-0.013	0.495	49.5	(38.570 to 60.405)	0.5	-0.013	(-0.291 to 0.264)
cg04608933	11	2554801	0.922	0.158	0.907	-0.092	0.464	46.4	(35.574 to 57.365)	0.497	-0.093	(-0.370 to 0.186)
cg04635504	11	2829241	2.297	0.151	2.468	1.122	0.134	13.4	(6.869 to 21.969)	0.42	1.133	(0.773 to 1.486)
cg04666029	11	2552843	-2.426	0.117	-1.883	4.582	0	0	(0.000 to 0.012)	0.004	4.628	(3.671 to 5.562)
cg04719766	11	2542580	2.983	0.206	3.086	0.493	0.312	31.2	(21.477 to 42.006)	0.48	0.498	(0.202 to 0.790)
cg04759114	11	2828274	2.053	0.161	2.001	-0.318	0.376	37.6	(27.300 to 48.600)	0.489	-0.321	(-0.604 to -0.035)
cg04762676	11	2722713	2.411	0.204	2.09	-1.556	0.063	6.3	(2.367 to 12.479)	0.346	-1.571	(-1.983 to -1.151)
cg04894537	11	2763171	-1.106	0.175	-0.272	4.705	0	0	(0.000 to 0.008)	0.003	4.752	(3.771 to 5.709)
cg04902871	11	2464954	1.482	0.271	1.279	-0.743	0.231	23.1	(14.419 to 33.248)	0.462	-0.75	(-1.062 to -0.433)
cg04907802	11	2753950	3.029	0.209	3.115	0.406	0.343	34.3	(24.287 to 45.242)	0.485	0.41	(0.120 to 0.697)
cg05125641	11	2503306	2.212	0.198	1.993	-1.095	0.139	13.9	(7.274 to 22.667)	0.423	-1.106	(-1.456 to -0.750)
cg05302416	11	2466037	-2.424	0.255	-2.675	-0.973	0.168	16.8	(9.384 to 26.082)	0.438	-0.983	(-1.317 to -0.641)
cg05418487	11	2466889	-3.5	0.164	-3.454	0.279	0.391	39.1	(28.668 to 50.093)	0.491	0.282	(-0.002 to 0.563)
cg05438727	11	2800047	-1.024	0.176	-1.11	-0.478	0.317	31.7	(21.950 to 42.558)	0.481	-0.483	(-0.774 to -0.188)
cg05624382	11	2780050	2.853	0.173	2.799	-0.311	0.378	37.8	(27.523 to 48.845)	0.489	-0.315	(-0.597 to -0.029)
cg05740879	11	2721866	0.179	0.104	-0.859	-9.878	0	0	(0.000 to 0.000)	0	-9.977	(-11.920 to -7.931)
cg05753930	11	2678450	2.642	0.248	2.105	-2.149	0.018	1.8	(0.372 to 4.884)	0.225	-2.17	(-2.677 to -1.656)
cg05760393	11	2466141	-4.031	0.394	-4.235	-0.513	0.305	30.5	(20.871 to 41.293)	0.479	-0.518	(-0.811 to -0.220)
cg05816130	11	2721799	0.13	0.1	-0.803	-9.27	0	0	(0.000 to 0.000)	0	-9.362	(-11.189 to -7.440)
cg05898618	11	2555576	1.989	0.391	2.823	2.111	0.02	2	(0.423 to 5.210)	0.232	2.132	(1.625 to 2.633)
cg06047778	11	2596300	2.623	0.129	2.585	-0.287	0.388	38.8	(28.370 to 49.769)	0.49	-0.29	(-0.572 to -0.006)
cg06159548	11	2481370	2.067	0.151	2.05	-0.114	0.455	45.5	(34.725 to 56.493)	0.497	-0.115	(-0.393 to 0.163)
cg06233503	11	2812674	-1.574	0.195	-1.238	1.713	0.047	4.7	(1.515 to 9.913)	0.314	1.73	(1.287 to 2.166)
cg06264108	11	2603429	3.279	0.183	3.292	0.068	0.473	47.3	(36.456 to 58.267)	0.498	0.069	(-0.209 to 0.346)
cg06288089	11	2722119	-0.1	0.089	-1.162	-11.834	0	0	(0.000 to 0.000)	0	-11.951	(-14.273 to -9.508)
cg06294475	11	2698623	-0.903	0.336	-0.338	1.665	0.051	5.1	(1.740 to 10.646)	0.324	1.682	(1.246 to 2.111)
cg06485603	11	2847258	-2.332	0.283	-1.837	1.729	0.045	4.5	(1.445 to 9.678)	0.311	1.746	(1.300 to 2.185)
cg06533200	11	2466138	-4.251	0.344	-4.497	-0.709	0.241	24.1	(15.297 to 34.402)	0.465	-0.716	(-1.024 to -0.402)
cg06585606	11	2680854	3.111	0.245	3.15	0.156	0.439	43.9	(33.171 to 54.880)	0.495	0.157	(-0.123 to 0.435)
cg06809295	11	2722625	2.653	0.158	2.299	-2.222	0.015	1.5	(0.286 to 4.286)	0.211	-2.245	(-2.764 to -1.718)

cg06827779	11	2603011	2.536	0.185	2.527	-0.048	0.481	48.1	(37.229 to 59.053)	0.499	-0.049	(-0.326 to 0.229)
cg06838584	11	2504581	2.929	0.309	2.871	-0.185	0.427	42.7	(32.086 to 53.743)	0.494	-0.187	(-0.465 to 0.094)
cg06850651	11	2785580	2.201	0.289	2.658	1.565	0.062	6.2	(2.308 to 12.316)	0.344	1.58	(1.159 to 1.994)
cg06873192	11	2598347	2.21	0.147	2.036	-1.175	0.123	12.3	(6.108 to 20.612)	0.412	-1.187	(-1.546 to -0.820)
cg06889525	11	2782615	2.503	0.147	2.651	0.996	0.162	16.2	(8.953 to 25.411)	0.435	1.006	(0.662 to 1.344)
cg06914593	11	2684842	2.674	0.137	2.686	0.087	0.466	46.6	(35.753 to 57.549)	0.498	0.088	(-0.190 to 0.365)
cg06960356	11	2596416	3.132	0.247	3.201	0.278	0.391	39.1	(28.704 to 50.131)	0.491	0.281	(-0.003 to 0.562)
cg07003739	11	2640527	1.612	0.158	1.581	-0.191	0.425	42.5	(31.872 to 53.516)	0.494	-0.192	(-0.471 to 0.088)
cg07040680	11	2621365	1.85	0.189	2.059	1.093	0.14	14	(7.312 to 22.731)	0.424	1.104	(0.748 to 1.453)
cg07072281	11	2713432	2.302	0.145	2.363	0.416	0.34	34	(23.972 to 44.885)	0.484	0.42	(0.129 to 0.707)
cg07123182	11	2722391	2.995	0.228	2.686	-1.341	0.093	9.3	(4.127 to 16.709)	0.385	-1.355	(-1.736 to -0.966)
cg07127259	11	2466042	-3.106	0.254	-3.109	-0.011	0.496	49.6	(38.648 to 60.484)	0.5	-0.011	(-0.288 to 0.266)
cg07210454	11	2482303	2.257	0.288	2.527	0.927	0.179	17.9	(10.284 to 27.449)	0.443	0.936	(0.599 to 1.266)
cg07549149	11	2560783	2.447	0.267	2.413	-0.125	0.45	45	(34.303 to 56.056)	0.496	-0.127	(-0.404 to 0.152)
cg07556018	11	2677341	-0.595	0.236	0.392	4.132	0	0	(0.000 to 0.048)	0.012	4.174	(3.302 to 5.040)
cg07592519	11	2735101	3.139	0.257	2.989	-0.577	0.283	28.3	(18.924 to 38.958)	0.475	-0.583	(-0.881 to -0.280)
cg07595203	11	2721480	-0.466	0.124	-1.374	-7.263	0	0	(0.000 to 0.000)	0	-7.335	(-8.776 to -5.819)
cg07618453	11	2502181	-2.178	0.246	-2.119	0.24	0.406	40.6	(30.061 to 51.593)	0.492	0.243	(-0.040 to 0.523)
cg07640406	11	2558029	2.389	0.172	2.357	-0.19	0.425	42.5	(31.908 to 53.554)	0.494	-0.191	(-0.470 to 0.089)
cg07683142	11	2704514	2.354	0.193	2.024	-1.694	0.048	4.8	(1.600 to 10.196)	0.318	-1.711	(-2.144 to -1.270)
cg07824422	11	2555406	-0.561	0.153	-0.268	1.891	0.032	3.2	(0.878 to 7.513)	0.278	1.91	(1.439 to 2.375)
cg07979757	11	2828652	1.908	0.293	2.129	0.746	0.23	23	(14.336 to 33.137)	0.462	0.754	(0.436 to 1.065)
cg08007665	11	2593857	2.139	0.194	2.213	0.379	0.353	35.3	(25.196 to 46.267)	0.486	0.383	(0.094 to 0.668)
cg08160246	11	2709914	1.374	0.12	1.29	-0.691	0.246	24.6	(15.758 to 34.998)	0.466	-0.698	(-1.004 to -0.385)
cg08249467	11	2646113	2.713	0.257	2.778	0.252	0.401	40.1	(29.641 to 51.142)	0.492	0.254	(-0.029 to 0.535)
cg08359167	11	2721351	0.076	0.122	-0.664	-6.017	0	0	(0.000 to 0.000)	0	-6.077	(-7.281 to -4.844)
cg08419850	11	2569801	3.414	0.176	3.249	-0.93	0.179	17.9	(10.222 to 27.357)	0.443	-0.939	(-1.269 to -0.602)
cg08446215	11	2721366	0.169	0.142	-0.999	-8.119	0	0	(0.000 to 0.000)	0	-8.2	(-9.805 to -6.511)
cg08861541	11	2610149	3.123	0.196	3.118	-0.023	0.491	49.1	(38.197 to 60.031)	0.499	-0.023	(-0.300 to 0.254)
cg08895013	11	2468332	-2.792	0.155	-2.845	-0.34	0.368	36.8	(26.527 to 47.749)	0.488	-0.343	(-0.627 to -0.056)
cg09197492	11	2571399	2.776	0.231	2.386	-1.672	0.05	5	(1.708 to 10.545)	0.322	-1.688	(-2.118 to -1.251)

cg09254350	11	2574026	3.139	0.251	2.977	-0.641	0.262	26.2	(17.105 to 36.710)	0.47	-0.648	(-0.950 to -0.340)
cg09315662	11	2612781	2.28	0.141	2.031	-1.757	0.043	4.3	(1.330 to 9.276)	0.305	-1.774	(-2.217 to -1.324)
cg09336947	11	2465950	-3.314	0.538	-3.469	-0.285	0.388	38.8	(28.441 to 49.846)	0.49	-0.288	(-0.570 to -0.004)
cg09390414	11	2571525	3.14	0.246	2.994	-0.587	0.28	28	(18.648 to 38.622)	0.474	-0.593	(-0.891 to -0.289)
cg09393494	11	2482401	2.35	0.167	2.34	-0.058	0.477	47.7	(36.836 to 58.654)	0.498	-0.059	(-0.336 to 0.219)
cg09518720	11	2721591	0.397	0.215	-0.597	-4.571	0	0	(0.000 to 0.013)	0.005	-4.617	(-5.548 to -3.662)
cg09916212	11	2498390	3.186	0.282	2.883	-1.065	0.146	14.6	(7.762 to 23.487)	0.427	-1.076	(-1.421 to -0.723)
cg10151367	11	2847642	-2.058	0.221	-2.301	-1.089	0.141	14.1	(7.369 to 22.828)	0.424	-1.1	(-1.449 to -0.745)
cg10425885	11	2458734	2.357	0.142	2.328	-0.201	0.421	42.1	(31.476 to 53.098)	0.494	-0.203	(-0.482 to 0.078)
cg10673351	11	2471097	2.515	0.183	2.651	0.734	0.233	23.3	(14.652 to 33.556)	0.463	0.741	(0.425 to 1.051)
cg10678459	11	2465103	1.961	0.243	1.581	-1.553	0.063	6.3	(2.383 to 12.523)	0.346	-1.569	(-1.980 to -1.149)
cg11025829	11	2465136	-1.14	0.16	-1.174	-0.212	0.416	41.6	(31.072 to 52.670)	0.493	-0.214	(-0.494 to 0.067)
cg11249934	11	2782519	1.325	0.188	1.032	-1.544	0.065	6.5	(2.445 to 12.693)	0.348	-1.559	(-1.969 to -1.141)
cg11272547	11	2722440	2.503	0.164	2.135	-2.224	0.015	1.5	(0.284 to 4.276)	0.21	-2.246	(-2.765 to -1.720)
cg11297256	11	2720810	0.473	0.157	-0.65	-7.099	0	0	(0.000 to 0.000)	0	-7.169	(-8.579 to -5.687)
cg11342234	11	2710838	0.694	0.185	0.375	-1.703	0.047	4.7	(1.560 to 10.065)	0.316	-1.72	(-2.155 to -1.278)
cg11363972	11	2465249	0.358	0.176	0.105	-1.428	0.08	8	(3.322 to 14.899)	0.37	-1.442	(-1.835 to -1.041)
cg11585942	11	2774756	2.388	0.149	2.61	1.468	0.074	7.4	(2.988 to 14.094)	0.362	1.483	(1.076 to 1.882)
cg11652182	11	2542384	2.984	0.213	3.125	0.655	0.258	25.8	(16.720 to 36.226)	0.469	0.662	(0.352 to 0.965)
cg11875582	11	2718307	2.287	0.325	2.494	0.628	0.266	26.6	(17.474 to 37.173)	0.471	0.634	(0.327 to 0.936)
cg11878045	11	2482536	1.897	0.153	1.757	-0.904	0.185	18.5	(10.743 to 28.130)	0.446	-0.913	(-1.240 to -0.579)
cg11993252	11	2721961	0.265	0.118	-0.55	-6.819	0	0	(0.000 to 0.000)	0	-6.887	(-8.244 to -5.461)
cg12077660	11	2721243	0.103	0.114	-0.651	-6.536	0	0	(0.000 to 0.000)	0	-6.601	(-7.903 to -5.266)
cg12141659	11	2847462	-1.5	0.209	-1.456	0.21	0.417	41.7	(31.168 to 52.772)	0.493	0.212	(-0.070 to 0.491)
cg12223985	11	2595800	2.974	0.211	3.111	0.645	0.261	26.1	(17.001 to 36.581)	0.47	0.651	(0.343 to 0.954)
cg12263990	11	2606365	2.736	0.158	2.442	-1.843	0.036	3.6	(1.023 to 8.115)	0.288	-1.861	(-2.318 to -1.397)
cg12424548	11	2591932	1.922	0.242	1.703	-0.896	0.187	18.7	(10.919 to 28.387)	0.447	-0.904	(-1.231 to -0.571)
cg12427469	11	2809574	2.67	0.147	2.647	-0.153	0.439	43.9	(33.261 to 54.974)	0.495	-0.155	(-0.433 to 0.125)
cg12433386	11	2722340	1.17	0.217	0.629	-2.475	0.008	0.8	(0.110 to 2.681)	0.162	-2.499	(-3.062 to -1.930)
cg12520982	11	2548920	1.88	0.144	2.136	1.764	0.042	4.2	(1.303 to 9.178)	0.304	1.781	(1.330 to 2.225)
cg12578166	11	2466909	-1.928	0.127	-1.989	-0.476	0.318	31.8	(22.019 to 42.639)	0.481	-0.481	(-0.772 to -0.186)

cg12851635	11	2826951	3.146	0.203	3.237	0.445	0.329	32.9	(23.004 to 43.778)	0.483	0.45	(0.157 to 0.739)
cg12901038	11	2815078	2.708	0.133	2.623	-0.634	0.264	26.4	(17.290 to 36.942)	0.471	-0.641	(-0.943 to -0.333)
cg12949760	11	2542862	-0.499	0.228	-0.12	1.645	0.053	5.3	(1.846 to 10.974)	0.328	1.661	(1.228 to 2.087)
cg13071812	11	2466788	-2.891	0.112	-2.869	0.195	0.423	42.3	(31.700 to 53.335)	0.494	0.197	(-0.084 to 0.476)
cg13145504	11	2594840	1.297	0.18	1.069	-1.252	0.108	10.8	(5.110 to 18.724)	0.4	-1.265	(-1.634 to -0.888)
cg13203297	11	2691249	2.943	0.196	3.253	1.566	0.062	6.2	(2.298 to 12.290)	0.344	1.582	(1.161 to 1.996)
cg13253153	11	2826988	2.843	0.282	3.1	0.905	0.185	18.5	(10.716 to 28.089)	0.446	0.914	(0.580 to 1.242)
cg13298691	11	2470675	3.49	0.286	3.468	-0.077	0.469	46.9	(36.127 to 57.932)	0.498	-0.078	(-0.355 to 0.200)
cg13428066	11	2677768	-0.914	0.216	-0.284	2.892	0.003	0.3	(0.019 to 1.137)	0.097	2.921	(2.278 to 3.557)
cg13444966	11	2705357	2.798	0.259	3.214	1.586	0.06	6	(2.177 to 11.950)	0.34	1.602	(1.178 to 2.019)
cg13536051	11	2594099	2.192	0.199	2.139	-0.263	0.397	39.7	(29.243 to 50.713)	0.491	-0.266	(-0.546 to 0.018)
cg13577072	11	2715286	1.418	0.159	1.59	1.072	0.144	14.4	(7.649 to 23.299)	0.426	1.083	(0.729 to 1.429)
cg13579167	11	2772852	3.052	0.195	3.71	3.335	0.001	0.1	(0.002 to 0.408)	0.05	3.369	(2.645 to 4.086)
cg13612693	11	2602171	2.401	0.138	2.279	-0.881	0.191	19.1	(11.219 to 28.823)	0.448	-0.89	(-1.215 to -0.559)
cg13742956	11	2612876	2.343	0.192	2.389	0.236	0.407	40.7	(30.214 to 51.756)	0.492	0.238	(-0.044 to 0.518)
cg13840782	11	2482394	3.045	0.246	2.872	-0.696	0.245	24.5	(15.623 to 34.823)	0.466	-0.703	(-1.010 to -0.390)
cg13859639	11	2846716	-0.382	0.142	0.074	3.182	0.001	0.1	(0.005 to 0.590)	0.064	3.214	(2.518 to 3.903)
cg13880539	11	2696589	2.905	0.234	3.205	1.27	0.105	10.5	(4.900 to 18.308)	0.397	1.283	(0.904 to 1.655)
cg13893637	11	2783554	2.019	0.185	2.139	0.645	0.261	26.1	(16.985 to 36.560)	0.47	0.652	(0.344 to 0.955)
cg14089425	11	2616834	2.221	0.445	1.949	-0.605	0.274	27.4	(18.123 to 37.977)	0.473	-0.611	(-0.911 to -0.306)
cg14243741	11	2721817	0.384	0.129	-0.645	-7.865	0	0	(0.000 to 0.000)	0	-7.944	(-9.500 to -6.306)
cg14384214	11	2817181	2.596	0.261	2.952	1.348	0.092	9.2	(4.057 to 16.557)	0.384	1.362	(0.972 to 1.744)
cg14392746	11	2721207	-0.151	0.118	-1.192	-8.737	0	0	(0.000 to 0.000)	0	-8.824	(-10.547 to -7.010)
cg14408823	11	2458481	1.834	0.157	2.166	2.092	0.021	2.1	(0.453 to 5.389)	0.236	2.113	(1.608 to 2.610)
cg14947466	11	2583705	2.385	0.156	2.284	-0.639	0.263	26.3	(17.169 to 36.791)	0.47	-0.645	(-0.947 to -0.337)
cg14958441	11	2721610	-0.366	0.083	-1.041	-8.019	0	0	(0.000 to 0.000)	0	-8.099	(-9.685 to -6.431)
cg15033776	11	2463739	3.001	0.309	2.525	-1.522	0.067	6.7	(2.591 to 13.081)	0.352	-1.537	(-1.945 to -1.123)
cg15074690	11	2571484	2.481	0.18	2.548	0.369	0.357	35.7	(25.553 to 46.666)	0.487	0.372	(0.084 to 0.657)
cg15422307	11	2693503	1.457	0.211	1.641	0.861	0.197	19.7	(11.649 to 29.442)	0.451	0.87	(0.541 to 1.193)
cg15467785	11	2783777	2.501	0.221	2.712	0.942	0.175	17.5	(9.973 to 26.982)	0.442	0.952	(0.613 to 1.283)
cg15651941	11	2722084	-0.065	0.135	-1.556	-10.945	0	0	(0.000 to 0.000)	0	-11.053	(-13.203 to -8.791)

cg15686157	11	2753885	1.801	0.178	1.914	0.627	0.267	26.7	(17.491 to 37.194)	0.471	0.634	(0.327 to 0.935)
cg15782852	11	2742507	2.629	0.181	2.48	-0.817	0.209	20.9	(12.652 to 30.852)	0.455	-0.825	(-1.143 to -0.500)
cg15861694	11	2706745	2.24	0.128	1.947	-2.263	0.014	1.4	(0.247 to 3.986)	0.203	-2.285	(-2.811 to -1.752)
cg15910264	11	2783880	2.391	0.169	2.258	-0.78	0.22	22	(13.510 to 32.029)	0.459	-0.788	(-1.103 to -0.467)
cg15932136	11	2465028	0.469	0.193	0.228	-1.239	0.111	11.1	(5.274 to 19.044)	0.402	-1.251	(-1.619 to -0.876)
cg16145209	11	2481909	2.145	0.128	2.259	0.875	0.193	19.3	(11.352 to 29.015)	0.449	0.884	(0.553 to 1.208)
cg16222896	11	2610580	2.886	0.209	3.127	1.147	0.129	12.9	(6.505 to 21.328)	0.416	1.158	(0.795 to 1.514)
cg16324958	11	2828568	2.779	0.27	3.169	1.432	0.079	7.9	(3.284 to 14.809)	0.369	1.446	(1.045 to 1.841)
cg16412789	11	2602926	3.337	0.205	3.456	0.577	0.283	28.3	(18.948 to 38.987)	0.475	0.582	(0.280 to 0.880)
cg16419342	11	2821200	2.811	0.188	2.761	-0.268	0.395	39.5	(29.074 to 50.532)	0.491	-0.27	(-0.551 to 0.013)
cg16434897	11	2772799	1.992	0.212	2.25	1.206	0.117	11.7	(5.697 to 19.851)	0.407	1.217	(0.847 to 1.581)
cg16465939	11	2554410	-1.79	0.143	-1.663	0.882	0.191	19.1	(11.208 to 28.807)	0.448	0.891	(0.559 to 1.216)
cg16556677	11	2722401	1.771	0.244	1.341	-1.746	0.044	4.4	(1.375 to 9.436)	0.307	-1.763	(-2.204 to -1.314)
cg16609872	11	2828364	1.548	0.165	1.674	0.761	0.225	22.5	(13.962 to 32.639)	0.46	0.769	(0.450 to 1.082)
cg16739686	11	2721336	0.138	0.154	-0.969	-7.112	0	0	(0.000 to 0.000)	0	-7.183	(-8.595 to -5.698)
cg16778148	11	2817922	2.575	0.173	2.51	-0.373	0.356	35.6	(25.414 to 46.511)	0.486	-0.376	(-0.662 to -0.088)
cg16811455	11	2742553	1.712	0.158	2.009	1.864	0.034	3.4	(0.957 to 7.847)	0.284	1.883	(1.415 to 2.343)
cg17035132	11	2504160	2.398	0.138	2.292	-0.763	0.224	22.4	(13.914 to 32.574)	0.46	-0.771	(-1.084 to -0.452)
cg17128405	11	2700832	2.755	0.302	2.463	-0.957	0.172	17.2	(9.678 to 26.535)	0.44	-0.967	(-1.300 to -0.627)
cg17227621	11	2741033	2.849	0.157	2.96	0.703	0.243	24.3	(15.428 to 34.572)	0.465	0.71	(0.397 to 1.018)
cg17229197	11	2542688	2.699	0.195	2.183	-2.617	0.006	0.6	(0.062 to 2.023)	0.137	-2.643	(-3.231 to -2.049)
cg17255148	11	2563802	2.615	0.228	2.743	0.552	0.292	29.2	(19.673 to 39.864)	0.476	0.558	(0.257 to 0.853)
cg17291157	11	2482284	2.364	0.227	2.417	0.234	0.408	40.8	(30.301 to 51.849)	0.492	0.236	(-0.046 to 0.516)
cg17305275	11	2746675	2.566	0.28	2.86	1.038	0.152	15.2	(8.219 to 24.238)	0.431	1.048	(0.699 to 1.390)
cg17333973	11	2847019	-0.52	0.136	0.051	4.161	0	0	(0.000 to 0.044)	0.011	4.203	(3.325 to 5.074)
cg17341170	11	2481545	2.697	0.149	2.897	1.333	0.094	9.4	(4.210 to 16.885)	0.387	1.347	(0.959 to 1.727)
cg17402907	11	2697109	2.789	0.385	3.241	1.161	0.126	12.6	(6.295 to 20.952)	0.414	1.173	(0.808 to 1.530)
cg17416793	11	2846932	-0.253	0.205	0.262	2.494	0.008	0.8	(0.102 to 2.580)	0.158	2.519	(1.946 to 3.085)
cg17494446	11	2569380	3.198	0.207	3.578	1.82	0.037	3.7	(1.097 to 8.409)	0.292	1.838	(1.378 to 2.291)
cg17575811	11	2466409	-2.758	0.227	-2.838	-0.348	0.365	36.5	(26.255 to 47.447)	0.488	-0.351	(-0.636 to -0.064)
cg17820828	11	2813298	0.227	0.391	-0.063	-0.734	0.233	23.3	(14.633 to 33.531)	0.462	-0.742	(-1.052 to -0.425)

cg18016139	11	2470969	2.798	0.19	2.978	0.936	0.177	17.7	(10.099 to 27.172)	0.442	0.945	(0.608 to 1.276)
cg18106482	11	2680314	3.19	0.225	3.065	-0.551	0.292	29.2	(19.700 to 39.896)	0.476	-0.557	(-0.852 to -0.256)
cg18272633	11	2848492	0.985	0.194	1.071	0.442	0.33	33	(23.108 to 43.897)	0.483	0.447	(0.154 to 0.735)
cg18450293	11	2548955	2.947	0.256	3.487	2.087	0.021	2.1	(0.461 to 5.437)	0.237	2.107	(1.604 to 2.604)
cg18477587	11	2672740	2.359	0.191	2.591	1.207	0.117	11.7	(5.678 to 19.817)	0.407	1.219	(0.848 to 1.582)
cg18729298	11	2555203	-1.673	0.178	-1.985	-1.736	0.044	4.4	(1.416 to 9.578)	0.309	-1.753	(-2.193 to -1.306)
cg18759359	11	2700935	2.868	0.2	2.56	-1.527	0.067	6.7	(2.555 to 12.987)	0.351	-1.543	(-1.951 to -1.127)
cg18865126	11	2596606	2.251	0.183	2.397	0.789	0.217	21.7	(13.293 to 31.734)	0.458	0.797	(0.475 to 1.113)
cg19095714	11	2645098	3.249	0.365	2.535	-1.937	0.029	2.9	(0.760 to 6.983)	0.268	-1.956	(-2.428 to -1.477)
cg19255554	11	2556743	1.55	0.152	1.69	0.91	0.184	18.4	(10.617 to 27.943)	0.445	0.919	(0.585 to 1.247)
cg19323901	11	2614062	2.272	0.135	2.196	-0.561	0.289	28.9	(19.401 to 39.536)	0.476	-0.567	(-0.863 to -0.265)
cg19388050	11	2562647	3.178	0.252	3.16	-0.07	0.472	47.2	(36.402 to 58.213)	0.498	-0.071	(-0.348 to 0.207)
cg19670883	11	2502357	-1.979	0.276	-1.934	0.163	0.436	43.6	(32.894 to 54.591)	0.495	0.165	(-0.115 to 0.443)
cg19672982	11	2715154	2.705	0.232	3.14	1.857	0.035	3.5	(0.979 to 7.939)	0.285	1.875	(1.409 to 2.334)
cg19678029	11	2722663	2.701	0.19	2.697	-0.022	0.491	49.1	(38.221 to 60.054)	0.499	-0.023	(-0.300 to 0.255)
cg19698309	11	2464959	2.626	0.184	2.508	-0.636	0.264	26.4	(17.239 to 36.878)	0.47	-0.643	(-0.945 to -0.335)
cg19728223	11	2813121	-2.152	0.411	-2.432	-0.675	0.252	25.2	(16.192 to 35.554)	0.468	-0.681	(-0.987 to -0.370)
cg19764489	11	2730616	1.699	0.233	1.51	-0.801	0.214	21.4	(13.023 to 31.365)	0.456	-0.809	(-1.125 to -0.486)
cg19779211	11	2465491	-2.104	0.17	-2.072	0.184	0.427	42.7	(32.115 to 53.772)	0.494	0.186	(-0.095 to 0.464)
cg19852660	11	2846681	0.892	0.161	1.096	1.258	0.107	10.7	(5.045 to 18.596)	0.399	1.27	(0.893 to 1.641)
cg19923326	11	2828778	1.862	0.2	2.115	1.248	0.109	10.9	(5.168 to 18.837)	0.4	1.26	(0.884 to 1.629)
cg19936757	11	2452218	1.298	0.146	1.273	-0.17	0.433	43.3	(32.636 to 54.320)	0.495	-0.172	(-0.450 to 0.109)
cg19975301	11	2779993	2.732	0.177	2.619	-0.632	0.265	26.5	(17.366 to 37.037)	0.471	-0.638	(-0.940 to -0.331)
cg20124933	11	2597564	3.192	0.298	3.051	-0.468	0.321	32.1	(22.289 to 42.953)	0.481	-0.472	(-0.762 to -0.178)
cg20170839	11	2597721	2.698	0.143	2.718	0.139	0.445	44.5	(33.781 to 55.516)	0.496	0.141	(-0.139 to 0.418)
cg20402210	11	2697527	1.947	0.129	2.117	1.304	0.099	9.9	(4.519 to 17.534)	0.391	1.317	(0.933 to 1.693)
cg20406758	11	2703128	1.917	0.228	1.705	-0.923	0.18	18	(10.352 to 27.550)	0.444	-0.932	(-1.262 to -0.596)
cg20429584	11	2461432	2.686	0.207	2.719	0.157	0.438	43.8	(33.106 to 54.812)	0.495	0.159	(-0.121 to 0.437)
cg20533553	11	2464822	1.832	0.167	1.964	0.785	0.218	21.8	(13.382 to 31.855)	0.458	0.793	(0.472 to 1.109)
cg20689362	11	2694005	2.276	0.117	2.181	-0.799	0.214	21.4	(13.060 to 31.415)	0.457	-0.807	(-1.124 to -0.484)
cg20699737	11	2721428	0.06	0.148	-0.847	-6.065	0	0	(0.000 to 0.000)	0	-6.126	(-7.338 to -4.883)

cg20751395	11	2594153	2.61	0.159	2.472	-0.857	0.198	19.8	(11.746 to 29.580)	0.451	-0.865	(-1.188 to -0.537)
cg20768429	11	2569832	3.186	0.261	3.462	1.049	0.15	15	(8.029 to 23.928)	0.429	1.059	(0.709 to 1.403)
cg20828897	11	2610595	2.659	0.228	2.712	0.23	0.41	41	(30.447 to 52.005)	0.492	0.232	(-0.050 to 0.512)
cg20850984	11	2569120	2.619	0.246	2.642	0.092	0.463	46.3	(35.544 to 57.335)	0.497	0.093	(-0.185 to 0.371)
cg20921686	11	2604499	2.552	0.195	2.876	1.64	0.054	5.4	(1.870 to 11.049)	0.329	1.656	(1.224 to 2.081)
cg21130221	11	2848310	-0.397	0.239	-0.062	1.389	0.086	8.6	(3.668 to 15.696)	0.377	1.403	(1.007 to 1.791)
cg21137515	11	2721857	0.066	0.097	-0.941	-10.324	0	0	(0.000 to 0.000)	0	-10.426	(-12.456 to -8.290)
cg21190593	11	2451058	1.113	0.268	0.882	-0.851	0.2	20	(11.885 to 29.777)	0.452	-0.859	(-1.181 to -0.531)
cg21695475	11	2612825	2.453	0.18	2.546	0.511	0.306	30.6	(20.932 to 41.365)	0.479	0.516	(0.218 to 0.809)
cg21752270	11	2813528	1.472	0.168	1.809	1.988	0.026	2.6	(0.643 to 6.422)	0.257	2.008	(1.520 to 2.488)
cg22047766	11	2560864	2.714	0.18	2.571	-0.785	0.218	21.8	(13.383 to 31.856)	0.458	-0.793	(-1.108 to -0.472)
cg22195512	11	2602980	2.085	0.124	1.917	-1.346	0.092	9.2	(4.079 to 16.605)	0.384	-1.359	(-1.742 to -0.970)
cg22239603	11	2690304	2.751	0.167	2.803	0.305	0.381	38.1	(27.761 to 49.105)	0.49	0.308	(0.022 to 0.590)
cg22296808	11	2560641	2.054	0.136	2.217	1.188	0.12	12	(5.929 to 20.284)	0.41	1.2	(0.832 to 1.561)
cg23040992	11	2603590	2.657	0.19	2.692	0.183	0.428	42.8	(32.136 to 53.795)	0.494	0.185	(-0.095 to 0.464)
cg23049234	11	2595718	2.824	0.183	2.874	0.268	0.395	39.5	(29.048 to 50.503)	0.491	0.271	(-0.013 to 0.552)
cg23267890	11	2464970	0.42	0.196	0.222	-1.003	0.16	16	(8.837 to 25.228)	0.435	-1.013	(-1.351 to -0.667)
cg23272088	11	2504813	2.916	0.296	2.272	-2.153	0.018	1.8	(0.366 to 4.846)	0.224	-2.175	(-2.682 to -1.660)
cg23545049	11	2569474	2.576	0.275	2.671	0.341	0.367	36.7	(26.489 to 47.707)	0.488	0.345	(0.058 to 0.628)
cg23623667	11	2465485	-2.406	0.248	-2.643	-0.946	0.174	17.4	(9.902 to 26.875)	0.441	-0.955	(-1.287 to -0.617)
cg23750514	11	2790418	1.67	0.275	2.162	1.769	0.042	4.2	(1.282 to 9.102)	0.303	1.787	(1.334 to 2.232)
cg23801748	11	2482735	3.336	0.204	3.085	-1.221	0.114	11.4	(5.494 to 19.468)	0.405	-1.233	(-1.599 to -0.861)
cg23903244	11	2812987	-1.949	0.502	-2.208	-0.511	0.306	30.6	(20.926 to 41.358)	0.479	-0.516	(-0.809 to -0.218)
cg23968213	11	2481965	2.034	0.166	2.04	0.035	0.486	48.6	(37.730 to 59.560)	0.499	0.035	(-0.242 to 0.313)
cg24073146	11	2543204	2.712	0.176	2.784	0.406	0.343	34.3	(24.281 to 45.235)	0.485	0.41	(0.120 to 0.697)
cg24079038	11	2465279	-0.404	0.17	-0.672	-1.558	0.063	6.3	(2.349 to 12.430)	0.345	-1.574	(-1.987 to -1.154)
cg24089935	11	2554295	-3.528	0.248	-3.453	0.299	0.383	38.3	(27.961 to 49.324)	0.49	0.302	(0.017 to 0.584)
cg24376802	11	2485050	2.305	0.137	2.192	-0.819	0.208	20.8	(12.592 to 30.769)	0.455	-0.827	(-1.146 to -0.502)
cg24478595	11	2817646	2.899	0.15	2.908	0.065	0.474	47.4	(36.579 to 58.393)	0.498	0.066	(-0.212 to 0.343)
cg24492694	11	2595605	3.005	0.375	2.902	-0.272	0.394	39.4	(28.935 to 50.381)	0.491	-0.274	(-0.555 to 0.010)
cg24609402	11	2598247	2.741	0.174	2.694	-0.267	0.395	39.5	(29.089 to 50.548)	0.491	-0.27	(-0.551 to 0.014)

cg24725201	11	2753818	2.934	0.174	3.048	0.65	0.259	25.9	(16.849 to 36.388)	0.469	0.657	(0.348 to 0.960)
cg24932449	11	2672613	2.497	0.221	2.408	-0.402	0.345	34.5	(24.426 to 45.399)	0.485	-0.406	(-0.693 to -0.116)
cg25173341	11	2828525	1.931	1.066	2.528	0.554	0.291	29.1	(19.629 to 39.812)	0.476	0.559	(0.258 to 0.855)
cg25175863	11	2604391	1.332	0.117	1.484	1.291	0.101	10.1	(4.659 to 17.823)	0.393	1.304	(0.922 to 1.679)
cg25204743	11	2715837	0.661	0.176	0.333	-1.838	0.036	3.6	(1.038 to 8.175)	0.289	-1.857	(-2.312 to -1.393)
cg25223664	11	2600975	1.768	0.16	1.936	1.038	0.152	15.2	(8.223 to 24.244)	0.431	1.048	(0.698 to 1.390)
cg25266888	11	2482594	2.268	0.219	1.867	-1.813	0.038	3.8	(1.123 to 8.509)	0.294	-1.831	(-2.283 to -1.372)
cg25306939	11	2722086	0.042	0.107	-1.348	-12.894	0	0	(0.000 to 0.000)	0	-13.022	(-15.549 to -10.363)
cg25356736	11	2775022	0.457	0.227	0.594	0.599	0.276	27.6	(18.306 to 38.203)	0.473	0.605	(0.300 to 0.904)
cg25513073	11	2481641	2.143	0.146	2.057	-0.585	0.281	28.1	(18.702 to 38.687)	0.474	-0.591	(-0.889 to -0.287)
cg25554751	11	2548989	2.761	0.147	2.582	-1.202	0.118	11.8	(5.742 to 19.936)	0.408	-1.214	(-1.577 to -0.844)
cg25741120	11	2482223	2.872	0.202	2.611	-1.284	0.103	10.3	(4.742 to 17.991)	0.395	-1.297	(-1.670 to -0.916)
cg25786675	11	2621273	3.084	0.206	3.029	-0.267	0.395	39.5	(29.091 to 50.549)	0.491	-0.27	(-0.551 to 0.014)
cg25946538	11	2482531	1.634	0.123	1.438	-1.584	0.06	6	(2.189 to 11.985)	0.34	-1.6	(-2.016 to -1.176)
cg25976755	11	2481495	2.814	0.189	2.916	0.534	0.298	29.8	(20.219 to 40.518)	0.478	0.539	(0.240 to 0.834)
cg26094482	11	2722073	-0.27	0.118	-1.261	-8.344	0	0	(0.000 to 0.000)	0	-8.427	(-10.075 to -6.693)
cg26104781	11	2721383	0.225	0.151	-0.858	-7.093	0	0	(0.000 to 0.000)	0	-7.164	(-8.572 to -5.682)
cg26190483	11	2689695	3.442	0.2	3.351	-0.453	0.326	32.6	(22.742 to 43.477)	0.482	-0.458	(-0.747 to -0.164)
cg26344859	11	2584629	2.518	0.143	2.463	-0.381	0.352	35.2	(25.124 to 46.186)	0.486	-0.385	(-0.671 to -0.096)
cg26411299	11	2598653	2.603	0.194	2.716	0.574	0.284	28.4	(19.020 to 39.074)	0.475	0.58	(0.277 to 0.877)
cg26417642	11	2466095	-3.592	0.222	-3.176	1.851	0.035	3.5	(0.997 to 8.009)	0.287	1.87	(1.404 to 2.328)
cg26438846	11	2731392	2.604	0.3	2.528	-0.251	0.401	40.1	(29.663 to 51.166)	0.492	-0.254	(-0.534 to 0.029)
cg26504274	11	2485409	3.559	0.214	3.269	-1.346	0.092	9.2	(4.079 to 16.604)	0.384	-1.359	(-1.742 to -0.970)
cg26512142	11	2554198	-2.898	0.091	-2.911	-0.137	0.446	44.6	(33.871 to 55.608)	0.496	-0.138	(-0.416 to 0.141)
cg26524638	11	2481449	2.718	0.192	2.966	1.278	0.104	10.4	(4.815 to 18.138)	0.396	1.29	(0.910 to 1.663)
cg26547719	11	2721437	-0.009	0.126	-0.793	-6.184	0	0	(0.000 to 0.000)	0	-6.245	(-7.480 to -4.979)
cg26639864	11	2569788	2.905	0.229	3.394	2.114	0.02	2	(0.420 to 5.187)	0.232	2.135	(1.627 to 2.636)
cg26671281	11	2828214	1.626	0.16	1.836	1.302	0.099	9.9	(4.540 to 17.578)	0.391	1.315	(0.932 to 1.691)
cg26709929	11	2482240	2.714	0.287	2.539	-0.604	0.274	27.4	(18.138 to 37.995)	0.473	-0.611	(-0.910 to -0.306)
cg26728427	11	2610741	2.684	0.161	2.849	1.016	0.157	15.7	(8.594 to 24.843)	0.433	1.026	(0.679 to 1.366)
cg26750319	11	2593960	2.739	0.153	2.724	-0.091	0.464	46.4	(35.594 to 57.386)	0.497	-0.092	(-0.369 to 0.186)

cg26879282	11	2817991	3.01	0.25	2.964	-0.18	0.429	42.9	(32.263 to 53.929)	0.494	-0.182	(-0.460 to 0.099)
cg26884837	11	2563897	2.367	0.293	2.653	0.964	0.17	17	(9.544 to 26.329)	0.439	0.974	(0.633 to 1.308)
cg26908876	11	2722076	-0.259	0.128	-1.54	-9.906	0	0	(0.000 to 0.000)	0	-10.005	(-11.954 to -7.953)
cg26929889	11	2562496	3.132	0.183	2.839	-1.59	0.059	5.9	(2.154 to 11.886)	0.339	-1.605	(-2.023 to -1.181)
cg26958174	11	2840699	2.614	0.161	2.431	-1.128	0.132	13.2	(6.777 to 21.809)	0.419	-1.139	(-1.493 to -0.779)
cg26963277	11	2722407	2.274	0.224	1.842	-1.912	0.031	3.1	(0.823 to 7.273)	0.273	-1.931	(-2.398 to -1.456)
cg27050114	11	2826795	3.172	0.325	3.157	-0.048	0.481	48.1	(37.253 to 59.077)	0.499	-0.048	(-0.325 to 0.230)
cg27119222	11	2720229	0.321	0.145	-1.235	-10.634	0	0	(0.000 to 0.000)	0	-10.739	(-12.829 to -8.540)
cg27246251	11	2559736	2.32	0.129	2.224	-0.74	0.231	23.1	(14.494 to 33.347)	0.462	-0.747	(-1.058 to -0.430)
cg27323091	11	2721619	0.064	0.099	-0.585	-6.463	0	0	(0.000 to 0.000)	0	-6.527	(-7.816 to -5.207)
cg27364242	11	2471064	1.837	0.135	2.213	2.759	0.004	0.4	(0.034 to 1.510)	0.115	2.787	(2.167 to 3.399)
cg27604721	11	2722258	0.411	0.132	-0.572	-7.399	0	0	(0.000 to 0.000)	0	-7.473	(-8.940 to -5.930)
cg27639104	11	2482929	3.267	0.24	2.848	-1.733	0.045	4.5	(1.426 to 9.613)	0.31	-1.751	(-2.190 to -1.304)

Supplementary Table 10. Performance of CH-test in detecting hypomethylation at the SNRPN locus in MLMD samples. The table presents 450k DNA methylation data across *SNRPN* region in BWS-MLMD4 and TND-MLMD5 samples. The point estimate represents the estimated percentage of the control population that would be expected to obtain lower score than the case and 95% confidence interval of point estimates were calculated from the noncentrality parameter from a noncentral t-distribution along with the point and interval estimates of the effect size. Here, ILMNID = Illumina Infinium HumanMethylation450 array probe ID, Chr = chromosome, Map information = genomic location (hg19), Mean = Control mean, and SD = control standard deviation, adjusted-p = Adjusted p-value using false discovery rate method, z_{cc} = point estimate of the effect size.

				Control group			Significance test		Estimated percentage of the control population obtaining a lower score than the case			Estimated effect size	
Case ID	ILMNID	Chr	Map Information	Mean	SD	Case score	t-value	p-value	Point	(95%CI)	Adjusted p	z_{cc}	(95%CI)
BWS-MLMD4	cg22259765	15	25119502	-0.7	0.261	-0.179	1.977	0.027	2.7	(0.666 to 6.533)	0.26	1.997	(1.511 to 2.475)
TND-MLMD5	cg22259765	15	25119502	-0.164	0.291	0.16	1.104	0.138	13.8	(7.143 to 22.443)	0.422	1.115	(0.757 to 1.465)
BWS-MLMD4	cg27644292	15	25123287	0.333	0.353	-1.11	-4.045	9.25E-05	0.00925	(4.01E-05 to 0.062)	0.014	-4.085	(-4.935 to -3.230)
TND-MLMD5	cg27644292	15	25123287	0.653	0.479	-1.121	-3.666	0	0	(0.000 to 0.176)	0.045	-3.702	(-4.481 to -2.918)
BWS-MLMD4	cg08560373	15	25123381	-0.975	0.344	-1.842	-2.495	0.008	0.8	(0.102 to 2.578)	0.158	-2.52	(-3.086 to -1.947)
TND-MLMD5	cg08560373	15	25123381	-0.652	0.516	-1.942	-2.476	0.008	0.8	(0.110 to 2.676)	0.194	-2.5	(-3.063 to -1.931)
BWS-MLMD4	cg25657700	15	25123491	0.409	0.207	-0.509	-4.399	2.93E-05	0.00293	(4.58E-06 to 0.022)	0.007	-4.443	(-5.342 to -3.520)
TND-MLMD5	cg25657700	15	25123491	0.717	0.247	-0.453	-4.688	1.12E-05	0.00112	(6.41E-07 to 0.009)	0.005	-4.735	(-5.689 to -3.758)
BWS-MLMD4	cg24993443	15	25123549	-0.197	0.389	-0.84	-1.634	0.054	5.4	(1.903 to 11.148)	0.33	-1.65	(-2.074 to -1.219)
TND-MLMD5	cg24993443	15	25123549	0.025	0.529	-0.94	-1.807	0.038	3.8	(1.144 to 8.590)	0.314	-1.825	(-2.275 to -1.366)
BWS-MLMD4	cg21870668	15	25123731	-0.298	0.244	-1.205	-3.67	0	0	(0.000 to 0.174)	0.028	-3.707	(-4.486 to -2.922)
TND-MLMD5	cg21870668	15	25123731	0.164	0.311	-0.974	-3.622	0	0	(0.000 to 0.198)	0.048	-3.658	(-4.428 to -2.881)
BWS-MLMD4	cg21061553	15	25124213	1.975	0.345	2.264	0.827	0.206	20.6	(12.403 to 30.506)	0.454	0.836	(0.510 to 1.155)
TND-MLMD5	cg21061553	15	25124213	2.269	0.387	2.583	0.804	0.213	21.3	(12.948 to 31.261)	0.454	0.812	(0.488 to 1.129)
BWS-MLMD4	cg12509344	15	25127701	2.655	0.295	2.693	0.128	0.449	44.9	(34.207 to 55.957)	0.496	0.129	(-0.150 to 0.407)
TND-MLMD5	cg12509344	15	25127701	2.664	0.252	2.645	-0.075	0.47	47	(36.204 to 58.010)	0.497	-0.076	(-0.353 to 0.202)
BWS-MLMD4	cg23075611	15	25145254	-0.378	0.142	-1.039	-4.621	1.40E-05	0.0014	(1.02E-06 to 0.011)	0.004	-4.667	(-5.608 to -3.703)
TND-MLMD5	cg23075611	15	25145254	-0.027	0.158	-0.921	-5.615	4.57E-07	4.57E-05	(5.31E-10 to 0.000)	0.00038	-5.67	(-6.798 to -4.515)
BWS-MLMD4	cg09206878	15	25165585	1.496	0.256	1.533	0.145	0.443	44.3	(33.572 to 55.298)	0.496	0.146	(-0.133 to 0.424)

TND-MLMD5	cg09206878	15	25165585	1.757	0.237	2.405	2.705	0.005	0.5	(0.043 to 1.692)	0.156	2.732	(2.122 to 3.334)
BWS-MLMD4	cg11152012	15	25196084	0.54	0.147	0.29	-1.685	0.049	4.9	(1.643 to 10.336)	0.319	-1.702	(-2.134 to -1.263)
TND-MLMD5	cg11152012	15	25196084	0.713	0.176	0.584	-0.724	0.236	23.6	(14.898 to 33.881)	0.461	-0.731	(-1.041 to -0.416)
BWS-MLMD4	cg21746532	15	25198793	2.171	0.402	2.731	1.379	0.087	8.7	(3.762 to 15.908)	0.378	1.392	(0.998 to 1.779)
TND-MLMD5	cg21746532	15	25198793	2.289	0.223	2.698	1.818	0.038	3.8	(1.105 to 8.442)	0.312	1.836	(1.376 to 2.289)
BWS-MLMD4	cg01432432	15	25199028	2.931	0.259	2.938	0.029	0.489	48.9	(37.980 to 59.811)	0.499	0.029	(-0.248 to 0.306)
TND-MLMD5	cg01432432	15	25199028	2.936	0.185	3.597	3.547	0	0	(0.001 to 0.240)	0.055	3.582	(2.820 to 4.339)
BWS-MLMD4	cg08372135	15	25199057	3.143	0.245	3.681	2.171	0.017	1.7	(0.343 to 4.693)	0.22	2.193	(1.675 to 2.703)
TND-MLMD5	cg08372135	15	25199057	3.038	0.214	3.02	-0.088	0.465	46.5	(35.728 to 57.524)	0.497	-0.088	(-0.366 to 0.190)
BWS-MLMD4	cg03858387	15	25199164	1.483	0.173	1.711	1.304	0.099	9.9	(4.516 to 17.529)	0.391	1.317	(0.933 to 1.694)
TND-MLMD5	cg03858387	15	25199164	1.571	0.207	1.99	2.001	0.025	2.5	(0.615 to 6.278)	0.278	2.021	(1.532 to 2.504)
BWS-MLMD4	cg02152271	15	25199270	2.38	0.328	2.122	-0.778	0.22	22	(13.564 to 32.102)	0.459	-0.786	(-1.100 to -0.465)
TND-MLMD5	cg02152271	15	25199270	2.433	0.165	2.378	-0.334	0.37	37	(26.741 to 47.985)	0.486	-0.337	(-0.621 to -0.051)
BWS-MLMD4	cg18506672	15	25200253	-0.132	0.104	-1.413	-12.226	1.11E-16	1.11E-14	(1.66E-47 to 4.43E-21)	5.37E-13	-12.347	(-14.745 to -9.824)
TND-MLMD5	cg18506672	15	25200253	0.082	0.179	-2.012	-11.598	6.66E-16	6.66E-14	(9.11E-43 to 5.94E-19)	1.77E-12	-11.713	(-13.989 to -9.318)
BWS-MLMD4	cg02125271	15	25200406	0.887	0.139	-0.47	-9.694	2.80E-13	2.80E-11	(6.49E-30 to 3.56E-13)	7.68E-10	-9.79	(-11.698 to -7.782)
TND-MLMD5	cg02125271	15	25200406	0.931	0.139	-1.141	-14.718	0	0	(9.54E-69 to 1.33E-30)	0	-14.864	(-17.744 to -11.832)
BWS-MLMD4	cg26875073	15	25200490	0.17	0.179	-1.382	-8.593	1.20E-11	1.20E-09	(1.62E-23 to 2.72E-10)	2.52E-08	-8.678	(-10.374 to -6.894)
TND-MLMD5	cg26875073	15	25200490	0.021	0.215	-1.646	-7.689	2.86E-10	2.86E-08	(7.86E-19 to 3.56E-08)	4.21E-07	-7.765	(-9.288 to -6.164)
BWS-MLMD4	cg22159025	15	25201020	-0.45	0.189	-1.972	-7.965	1.08E-10	1.08E-08	(3.29E-20 to 8.46E-09)	1.99E-07	-8.045	(-9.620 to -6.387)
TND-MLMD5	cg22159025	15	25201020	-0.236	0.245	-1.781	-6.232	5.13E-08	5.13E-06	(2.37E-12 to 2.60E-05)	5.29E-05	-6.294	(-7.539 to -5.019)
BWS-MLMD4	cg22555495	15	25201224	-0.168	0.086	-1.165	-11.464	8.88E-16	8.88E-14	(8.61E-42 to 1.63E-18)	3.69E-12	-11.578	(-13.828 to -9.210)
TND-MLMD5	cg22555495	15	25201224	0.374	0.136	-1.033	-10.268	4.15E-14	4.15E-12	(1.50E-33 to 8.23E-15)	9.59E-11	-10.37	(-12.389 to -8.245)
BWS-MLMD4	cg01614564	15	25201429	0.165	0.104	-0.706	-8.295	3.39E-11	3.39E-09	(6.45E-22 to 1.43E-09)	6.74E-08	-8.378	(-10.017 to -6.653)
TND-MLMD5	cg01614564	15	25201429	0.662	0.096	-0.567	-12.642	0	0	(8.75E-51 to 1.50E-22)	0	-12.768	(-15.246 to -10.160)
BWS-MLMD4	cg13073261	15	25201732	-0.01	0.129	-1.306	-9.926	1.29E-13	1.29E-11	(2.31E-31 to 7.94E-14)	3.71E-10	-10.025	(-11.978 to -7.970)
TND-MLMD5	cg13073261	15	25201732	0.002	0.17	-1.325	-7.726	2.51E-10	2.51E-08	(5.18E-19 to 2.94E-08)	3.71E-07	-7.803	(-9.332 to -6.193)
BWS-MLMD4	cg20775837	15	25203270	2.194	0.321	2.293	0.304	0.381	38.1	(27.797 to 49.144)	0.49	0.307	(0.021 to 0.589)
TND-MLMD5	cg20775837	15	25203270	2.175	0.245	2.668	1.994	0.026	2.6	(0.630 to 6.354)	0.279	2.014	(1.526 to 2.495)
BWS-MLMD4	cg14851390	15	25215375	2.34	0.253	2.214	-0.496	0.311	31.1	(21.377 to 41.889)	0.48	-0.501	(-0.793 to -0.205)
TND-MLMD5	cg14851390	15	25215375	2.247	0.232	2.564	1.354	0.091	9.1	(3.999 to 16.432)	0.39	1.368	(0.977 to 1.751)

BWS-MLMD4	cg25705379	15	25221513	1.87	0.224	2.061	0.841	0.202	20.2	(12.093 to 30.071)	0.453	0.85	(0.522 to 1.170)
TND-MLMD5	cg25705379	15	25221513	2.107	0.193	2.401	1.508	0.069	6.9	(2.691 to 13.342)	0.366	1.523	(1.110 to 1.928)
BWS-MLMD4	cg04195863	15	25223574	1.786	0.205	2.25	2.246	0.015	1.5	(0.263 to 4.112)	0.206	2.268	(1.738 to 2.791)
TND-MLMD5	cg04195863	15	25223574	2.013	0.273	2.599	2.12	0.02	2	(0.410 to 5.129)	0.256	2.142	(1.632 to 2.643)
BWS-MLMD4	cg26582085	15	25225929	2.559	0.286	3.089	1.835	0.036	3.6	(1.047 to 8.211)	0.29	1.854	(1.391 to 2.309)
TND-MLMD5	cg26582085	15	25225929	2.909	0.275	2.683	-0.814	0.21	21	(12.716 to 30.942)	0.453	-0.822	(-1.140 to -0.498)
BWS-MLMD4	cg26724572	15	25226703	2.829	0.281	2.83	0.002	0.499	49.9	(38.991 to 60.827)	0.5	0.002	(-0.275 to 0.280)
TND-MLMD5	cg26724572	15	25226703	2.827	0.183	2.745	-0.44	0.331	33.1	(23.168 to 43.966)	0.48	-0.445	(-0.733 to -0.152)
BWS-MLMD4	cg11166999	15	25226929	3.24	0.353	3.194	-0.131	0.448	44.8	(34.104 to 55.850)	0.496	-0.132	(-0.410 to 0.147)
TND-MLMD5	cg11166999	15	25226929	3.266	0.257	3.307	0.16	0.437	43.7	(33.011 to 54.713)	0.494	0.161	(-0.118 to 0.440)
BWS-MLMD4	cg22934878	15	25227003	3.078	0.232	3.312	1.001	0.161	16.1	(8.863 to 25.269)	0.435	1.011	(0.666 to 1.349)
TND-MLMD5	cg22934878	15	25227003	3.126	0.157	2.956	-1.069	0.145	14.5	(7.700 to 23.384)	0.426	-1.079	(-1.426 to -0.726)
BWS-MLMD4	cg05894557	15	25227036	2.074	0.22	2.67	2.679	0.005	0.5	(0.048 to 1.782)	0.127	2.706	(2.101 to 3.304)
TND-MLMD5	cg05894557	15	25227036	2.137	0.236	3.297	4.873	5.97E-06	0.000597	(1.72E-07 to 0.005)	0.003	4.921	(3.909 to 5.909)
BWS-MLMD4	cg14853933	15	25228929	1.533	0.244	2.415	3.573	0	0	(0.001 to 0.225)	0.034	3.608	(2.841 to 4.369)
TND-MLMD5	cg14853933	15	25228929	1.738	0.277	2.299	2.002	0.025	2.5	(0.614 to 6.276)	0.278	2.021	(1.532 to 2.504)
BWS-MLMD4	cg26410550	15	25229256	2.176	0.352	2.643	1.313	0.098	9.8	(4.420 to 17.328)	0.39	1.326	(0.941 to 1.704)
TND-MLMD5	cg26410550	15	25229256	2.259	0.204	3.001	3.597	0	0	(0.001 to 0.211)	0.05	3.633	(2.861 to 4.399)
BWS-MLMD4	cg02891726	15	25229522	2.482	0.193	3.041	2.866	0.003	0.3	(0.021 to 1.202)	0.1	2.895	(2.256 to 3.527)
TND-MLMD5	cg02891726	15	25229522	2.262	0.142	2.311	0.343	0.366	36.6	(26.411 to 47.620)	0.485	0.347	(0.060 to 0.631)

Supplementary Table 11. Performance of CH-test in detecting hypomethylation at the GNAS locus in MLMD samples. The table presents 450k DNA methylation data across *GNAS* region in TND-MLMD2 and BWS-MLMD4 samples. The point estimate represents the estimated percentage of the control population that would be expected to obtain lower score than the case and 95% confidence interval of point estimates were calculated from the noncentrality parameter from a noncentral t-distribution along with the point and interval estimates of the effect size. Here, ILMNID = Illumina Infinium HumanMethylation450 array probe ID, Chr = chromosome, Map information = genomic location (hg19), Mean = Control mean, and SD = control standard deviation, adjusted-p = Adjusted p-value using false discovery rate method, z_{cc} = point estimate of the effect size.

				Control group			Significance test		Estimated percentage of the control population obtaining a lower score than the case			Estimated effect size	
Case ID	ILMNIND	Chr	Map Information	Mean	SD	Case score	t-value	p-value	Point	(95%CI)	Adjusted p	Z_{cc}	(95%CI)
BWS-MLMD4	cg07050493	20	57392572	2.521	0.163	2.539	0.113	0.455	45.5	(34.778 to 56.547)	0.497	0.114	(-0.165 to 0.391)
TND-MLMD2	cg07050493	20	57392572	2.713	0.158	2.821	0.678	0.25	25	(16.094 to 35.430)	0.457	0.685	(0.374 to 0.991)
BWS-MLMD4	cg13869899	20	57392884	2.731	0.192	2.973	1.246	0.109	10.9	(5.185 to 18.871)	0.401	1.259	(0.883 to 1.627)
TND-MLMD2	cg13869899	20	57392884	2.606	0.099	2.29	-3.153	0.001	0.1	(0.005 to 0.630)	0.054	-3.185	(-3.869 to -2.495)
BWS-MLMD4	cg14977365	20	57392898	2.703	0.265	2.77	0.25	0.402	40.2	(29.718 to 51.225)	0.492	0.252	(-0.031 to 0.533)
TND-MLMD2	cg14977365	20	57392898	2.703	0.248	2.468	-0.938	0.176	17.6	(10.056 to 27.108)	0.428	-0.947	(-1.278 to -0.610)
BWS-MLMD4	cg25188071	20	57393277	2.371	0.215	1.799	-2.637	0.006	0.6	(0.057 to 1.943)	0.134	-2.663	(-3.254 to -2.066)
TND-MLMD2	cg25188071	20	57393277	2.439	0.235	2.232	-0.872	0.194	19.4	(11.417 to 29.108)	0.436	-0.881	(-1.205 to -0.550)
BWS-MLMD4	cg21983491	20	57393380	1.26	0.119	1.175	-0.699	0.244	24.4	(15.533 to 34.708)	0.466	-0.706	(-1.014 to -0.393)
TND-MLMD2	cg21983491	20	57393380	1.455	0.096	1.597	1.46	0.075	7.5	(3.058 to 14.264)	0.342	1.474	(1.069 to 1.872)
BWS-MLMD4	cg11954355	20	57393844	-0.554	0.25	-0.93	-1.49	0.071	7.1	(2.822 to 13.677)	0.358	-1.505	(-1.908 to -1.095)
TND-MLMD2	cg11954355	20	57393844	-0.086	0.294	-0.511	-1.43	0.08	8	(3.304 to 14.856)	0.348	-1.444	(-1.838 to -1.043)
BWS-MLMD4	cg03030267	20	57394000	1.19	0.405	0.808	-0.935	0.177	17.7	(10.119 to 27.202)	0.442	-0.944	(-1.275 to -0.607)
TND-MLMD2	cg03030267	20	57394000	1.604	0.361	1.002	-1.653	0.052	5.2	(1.801 to 10.836)	0.302	-1.67	(-2.097 to -1.235)
BWS-MLMD4	cg15428904	20	57394097	2.268	0.262	2.566	1.131	0.132	13.2	(6.728 to 21.723)	0.418	1.143	(0.782 to 1.496)
TND-MLMD2	cg15428904	20	57394097	2.514	0.2	2.576	0.302	0.382	38.2	(27.843 to 49.195)	0.486	0.305	(0.020 to 0.587)
BWS-MLMD4	cg10309886	20	57407747	-1.724	0.191	-1.008	3.702	0	0	(0.000 to 0.160)	0.027	3.739	(2.948 to 4.524)
TND-MLMD2	cg10309886	20	57407747	-1.419	0.296	-0.105	4.396	2.96E-05	0.00296	(4.69E-06 to 0.022)	0.007	4.439	(3.518 to 5.338)
BWS-MLMD4	cg03613625	20	57408174	1.036	0.143	1.328	2.021	0.024	2.4	(0.576 to 6.078)	0.251	2.041	(1.548 to 2.526)
TND-MLMD2	cg03613625	20	57408174	1.392	0.126	1.673	2.201	0.016	1.6	(0.309 to 4.452)	0.187	2.223	(1.700 to 2.739)

BWS-MLMD4	cg23699918	20	57408730	0.266	0.1	0.581	3.132	0.001	0.1	(0.006 to 0.662)	0.069	3.164	(2.477 to 3.844)
TND-MLMD2	cg23699918	20	57408730	0.769	0.059	1.186	6.999	3.32E-09	3.32E-07	(1.34E-15 to 1.03E-06)	4.05E-06	7.069	(5.606 to 8.460)
BWS-MLMD4	cg00636769	20	57408795	1.857	0.282	2.155	1.043	0.151	15.1	(8.127 to 24.088)	0.43	1.054	(0.703 to 1.397)
TND-MLMD2	cg00636769	20	57408795	2.129	0.274	2.44	1.123	0.133	13.3	(6.846 to 21.930)	0.402	1.135	(0.775 to 1.487)
BWS-MLMD4	cg26873158	20	57409309	1.967	0.211	2.108	0.661	0.256	25.6	(16.549 to 36.009)	0.469	0.668	(0.358 to 0.972)
TND-MLMD2	cg26873158	20	57409309	2.159	0.175	2.501	1.932	0.03	3	(0.772 to 7.039)	0.244	1.951	(1.473 to 2.422)
BWS-MLMD4	cg16682926	20	57411775	2.798	0.237	3.063	1.103	0.138	13.8	(7.151 to 22.458)	0.422	1.114	(0.757 to 1.465)
TND-MLMD2	cg16682926	20	57411775	2.732	0.172	2.948	1.249	0.109	10.9	(5.151 to 18.805)	0.382	1.261	(0.885 to 1.630)
BWS-MLMD4	cg04780380	20	57412945	1.223	0.479	1.438	0.442	0.33	33	(23.108 to 43.897)	0.483	0.447	(0.154 to 0.735)
TND-MLMD2	cg04780380	20	57412945	1.738	0.304	1.873	0.44	0.331	33.1	(23.161 to 43.959)	0.477	0.445	(0.152 to 0.734)
BWS-MLMD4	cg07964163	20	57413417	0.977	0.21	1.077	0.472	0.32	32	(22.154 to 42.796)	0.481	0.476	(0.182 to 0.767)
TND-MLMD2	cg07964163	20	57413417	1.233	0.174	1.704	2.676	0.005	0.5	(0.048 to 1.794)	0.106	2.703	(2.098 to 3.301)
BWS-MLMD4	cg10459054	20	57413436	2.476	0.178	2.6	0.692	0.246	24.6	(15.718 to 34.946)	0.466	0.699	(0.387 to 1.006)
TND-MLMD2	cg10459054	20	57413436	2.49	0.139	2.762	1.928	0.03	3	(0.781 to 7.083)	0.245	1.947	(1.470 to 2.417)
BWS-MLMD4	cg27642181	20	57413694	2.91	0.181	2.917	0.035	0.486	48.6	(37.731 to 59.561)	0.499	0.035	(-0.242 to 0.313)
TND-MLMD2	cg27642181	20	57413694	2.943	0.184	2.842	-0.542	0.295	29.5	(19.961 to 40.210)	0.469	-0.548	(-0.843 to -0.248)
BWS-MLMD4	cg17071192	20	57413698	2.795	0.216	3.037	1.108	0.137	13.7	(7.080 to 22.335)	0.421	1.119	(0.761 to 1.470)
TND-MLMD2	cg17071192	20	57413698	2.871	0.175	2.833	-0.214	0.416	41.6	(31.016 to 52.611)	0.491	-0.216	(-0.495 to 0.066)
BWS-MLMD4	cg07434855	20	57413700	2.192	0.208	2.094	-0.468	0.321	32.1	(22.280 to 42.943)	0.481	-0.472	(-0.763 to -0.178)
TND-MLMD2	cg07434855	20	57413700	2.16	0.143	1.799	-2.494	0.008	0.8	(0.102 to 2.584)	0.133	-2.518	(-3.084 to -1.946)
BWS-MLMD4	cg04132853	20	57414039	0.284	0.18	0.541	1.417	0.081	8.1	(3.411 to 15.106)	0.371	1.431	(1.032 to 1.824)
TND-MLMD2	cg04132853	20	57414039	0.752	0.127	1.768	7.934	1.20E-10	1.20E-08	(4.73E-20 to 9.98E-09)	1.81E-07	8.013	(6.362 to 9.583)
BWS-MLMD4	cg25090051	20	57414059	0.017	0.163	0.389	2.264	0.014	1.4	(0.246 to 3.979)	0.203	2.286	(1.753 to 2.812)
TND-MLMD2	cg25090051	20	57414059	0.428	0.12	1.165	6.068	9.20E-08	9.20E-06	(1.06E-11 to 5.18E-05)	8.03E-05	6.128	(4.885 to 7.341)
BWS-MLMD4	cg00732970	20	57414162	-0.583	0.107	-0.4	1.697	0.048	4.8	(1.589 to 10.159)	0.317	1.714	(1.273 to 2.147)
TND-MLMD2	cg00732970	20	57414162	0.123	0.11	0.971	7.599	3.94E-10	3.94E-08	(2.15E-18 to 5.61E-08)	5.42E-07	7.675	(6.091 to 9.180)
BWS-MLMD4	cg17696847	20	57414217	-0.118	0.218	-0.071	0.214	0.416	41.6	(31.011 to 52.606)	0.493	0.216	(-0.065 to 0.496)
TND-MLMD2	cg17696847	20	57414217	0.365	0.143	1.045	4.716	1.02E-05	0.00102	(5.29E-07 to 0.008)	0.004	4.763	(3.780 to 5.721)
BWS-MLMD4	cg23732978	20	57414274	0.423	0.624	1.201	1.235	0.111	11.1	(5.319 to 19.132)	0.402	1.248	(0.873 to 1.615)
TND-MLMD2	cg23732978	20	57414274	0.749	0.64	1.266	0.8	0.214	21.4	(13.048 to 31.398)	0.444	0.807	(0.485 to 1.124)
BWS-MLMD4	cg20019489	20	57414351	0.727	0.318	0.924	0.614	0.271	27.1	(17.858 to 37.650)	0.472	0.62	(0.315 to 0.921)

TND-MLMD2	cg20019489	20	57414351	1.16	0.217	1.949	3.605	0	0	(0.001 to 0.207)	0.028	3.64	(2.867 to 4.408)
BWS-MLMD4	cg02274728	20	57414407	0.044	0.126	0.402	2.818	0.003	0.3	(0.026 to 1.333)	0.107	2.846	(2.216 to 3.469)
TND-MLMD2	cg02274728	20	57414407	0.49	0.137	1.382	6.447	2.39E-08	2.39E-06	(3.19E-13 to 1.03E-05)	2.46E-05	6.511	(5.194 to 7.796)
BWS-MLMD4	cg12054318	20	57414529	0.081	0.1	0.221	1.39	0.085	8.5	(3.660 to 15.679)	0.377	1.403	(1.008 to 1.792)
TND-MLMD2	cg12054318	20	57414529	0.433	0.15	1.253	5.423	8.94E-07	8.94E-05	(2.55E-09 to 0.001)	0.001	5.477	(4.358 to 6.568)
BWS-MLMD4	cg11996914	20	57414578	0.15	0.109	-0.171	-2.904	0.003	0.3	(0.018 to 1.107)	0.095	-2.933	(-3.572 to -2.288)
TND-MLMD2	cg11996914	20	57414578	0.202	0.127	0.589	3.03	0.002	0.2	(0.010 to 0.837)	0.064	3.06	(2.392 to 3.722)
BWS-MLMD4	cg21330323	20	57414596	-0.006	0.108	-0.343	-3.097	0.002	0.2	(0.007 to 0.718)	0.073	-3.128	(-3.801 to -2.448)
TND-MLMD2	cg21330323	20	57414596	0.06	0.144	0.28	1.516	0.068	6.8	(2.637 to 13.201)	0.331	1.531	(1.117 to 1.937)
BWS-MLMD4	cg06163629	20	57414884	0.045	0.144	-0.182	-1.555	0.063	6.3	(2.370 to 12.488)	0.346	-1.571	(-1.983 to -1.151)
TND-MLMD2	cg06163629	20	57414884	-0.011	0.14	0.46	3.343	0.001	0.1	(0.002 to 0.401)	0.041	3.376	(2.652 to 4.095)
BWS-MLMD4	cg14597908	20	57414960	-0.2	0.084	-0.287	-1.024	0.155	15.5	(8.451 to 24.613)	0.432	-1.035	(-1.375 to -0.687)
TND-MLMD2	cg14597908	20	57414960	0.211	0.128	0.783	4.404	2.89E-05	0.00289	(4.45E-06 to 0.021)	0.007	4.447	(3.524 to 5.348)
BWS-MLMD4	cg00765653	20	57415144	0.265	0.128	-0.013	-2.147	0.018	1.8	(0.374 to 4.897)	0.225	-2.168	(-2.675 to -1.655)
TND-MLMD2	cg00765653	20	57415144	0.314	0.122	0.753	3.548	0	0	(0.001 to 0.240)	0.03	3.583	(2.820 to 4.340)
BWS-MLMD4	cg14728235	20	57415177	0.234	0.123	-0.025	-2.079	0.021	2.1	(0.474 to 5.512)	0.239	-2.099	(-2.594 to -1.597)
TND-MLMD2	cg14728235	20	57415177	0.356	0.13	0.669	2.386	0.01	1	(0.156 to 3.175)	0.152	2.41	(1.856 to 2.957)
BWS-MLMD4	cg05558390	20	57415377	-0.753	0.146	-0.931	-1.207	0.117	11.7	(5.679 to 19.817)	0.407	-1.219	(-1.582 to -0.848)
TND-MLMD2	cg05558390	20	57415377	-0.669	0.186	-0.142	2.81	0.004	0.4	(0.027 to 1.357)	0.088	2.838	(2.209 to 3.459)
BWS-MLMD4	cg19296354	20	57415697	-0.04	0.132	0.065	0.782	0.219	21.9	(13.461 to 31.963)	0.458	0.79	(0.469 to 1.105)
TND-MLMD2	cg19296354	20	57415697	0.331	0.173	0.976	3.7	0	0	(0.000 to 0.161)	0.024	3.736	(2.946 to 4.521)
BWS-MLMD4	cg24975842	20	57416221	-0.197	0.118	-0.409	-1.786	0.04	4	(1.217 to 8.867)	0.299	-1.804	(-2.252 to -1.349)
TND-MLMD2	cg24975842	20	57416221	-0.022	0.182	0.347	2.012	0.025	2.5	(0.594 to 6.173)	0.226	2.032	(1.540 to 2.515)
BWS-MLMD4	cg18619398	20	57416506	-0.095	0.104	-0.055	0.386	0.351	35.1	(24.971 to 46.014)	0.486	0.39	(0.100 to 0.675)
TND-MLMD2	cg18619398	20	57416506	0.112	0.132	0.469	2.684	0.005	0.5	(0.047 to 1.764)	0.104	2.711	(2.105 to 3.310)
BWS-MLMD4	cg00952145	20	57417152	-0.651	0.097	-0.59	0.615	0.271	27.1	(17.844 to 37.632)	0.472	0.621	(0.315 to 0.921)
TND-MLMD2	cg00952145	20	57417152	-0.238	0.161	0.438	4.16	6.39E-05	0.00639	(1.96E-05 to 0.044)	0.011	4.202	(3.324 to 5.073)
BWS-MLMD4	cg20582984	20	57417233	0.252	0.127	0.198	-0.423	0.337	33.7	(23.744 to 44.626)	0.484	-0.427	(-0.715 to -0.135)
TND-MLMD2	cg20582984	20	57417233	0.874	0.165	1.675	4.804	7.56E-06	0.000756	(2.83E-07 to 0.006)	0.003	4.852	(3.852 to 5.827)
BWS-MLMD4	cg18949315	20	57418015	-0.035	0.139	-0.119	-0.598	0.276	27.6	(18.338 to 38.241)	0.473	-0.603	(-0.903 to -0.299)
TND-MLMD2	cg18949315	20	57418015	0.391	0.119	1.298	7.521	5.20E-10	5.20E-08	(5.15E-18 to 8.32E-08)	7.05E-07	7.595	(6.028 to 9.086)

BWS-MLMD4	cg14943539	20	57420942	1.126	0.231	1.088	-0.161	0.436	43.6	(32.958 to 54.657)	0.495	-0.163	(-0.441 to 0.117)
TND-MLMD2	cg14943539	20	57420942	1.522	0.159	1.802	1.753	0.043	4.3	(1.345 to 9.329)	0.281	1.771	(1.321 to 2.213)
BWS-MLMD4	cg21779904	20	57425157	-0.414	0.109	-0.004	3.73	0	0	(0.000 to 0.149)	0.025	3.767	(2.971 to 4.557)
TND-MLMD2	cg21779904	20	57425157	-0.014	0.187	0.498	2.712	0.005	0.5	(0.041 to 1.667)	0.101	2.739	(2.128 to 3.343)
BWS-MLMD4	cg03547757	20	57425515	1.422	0.196	1.3	-0.617	0.27	27	(17.790 to 37.565)	0.472	-0.623	(-0.923 to -0.317)
TND-MLMD2	cg03547757	20	57425515	1.64	0.21	1.191	-2.116	0.02	2	(0.417 to 5.168)	0.204	-2.137	(-2.638 to -1.629)
BWS-MLMD4	cg18935491	20	57425979	0.244	0.122	-0.341	-4.731	9.66E-06	0.000966	(4.74E-07 to 0.007)	0.003	-4.778	(-5.740 to -3.793)
TND-MLMD2	cg18935491	20	57425979	0.186	0.158	-0.769	-5.977	1.27E-07	1.27E-05	(2.37E-11 to 7.52E-05)	0	-6.037	(-7.233 to -4.811)
BWS-MLMD4	cg24203465	20	57425986	0.049	0.097	-0.165	-2.192	0.017	1.7	(0.319 to 4.527)	0.216	-2.214	(-2.728 to -1.693)
TND-MLMD2	cg24203465	20	57425986	0.417	0.149	-0.739	-7.704	2.72E-10	2.72E-08	(6.63E-19 to 3.29E-08)	3.87E-07	-7.781	(-9.306 to -6.176)
BWS-MLMD4	cg26102503	20	57425994	-0.171	0.148	-0.419	-1.663	0.051	5.1	(1.753 to 10.687)	0.324	-1.679	(-2.108 to -1.243)
TND-MLMD2	cg26102503	20	57425994	0.293	0.21	-1.036	-6.259	4.66E-08	4.66E-06	(1.85E-12 to 2.32E-05)	4.50E-05	-6.321	(-7.571 to -5.041)
BWS-MLMD4	cg03344105	20	57426131	-0.131	0.083	-0.517	-4.582	1.60E-05	0.0016	(1.34E-06 to 0.012)	0.004	-4.627	(-5.561 to -3.670)
TND-MLMD2	cg03344105	20	57426131	0.307	0.158	-0.932	-7.772	2.14E-10	2.14E-08	(3.07E-19 to 2.33E-08)	3.07E-07	-7.849	(-9.388 to -6.230)
BWS-MLMD4	cg14660776	20	57426138	-0.376	0.082	-0.633	-3.125	0.001	0.1	(0.006 to 0.674)	0.069	-3.156	(-3.834 to -2.471)
TND-MLMD2	cg14660776	20	57426138	-0.019	0.134	-1.298	-9.459	6.17E-13	6.17E-11	(1.74E-28 to 1.57E-12)	1.29E-09	-9.553	(-11.416 to -7.593)
BWS-MLMD4	cg19592829	20	57426215	-0.07	0.082	-0.333	-3.191	0.001	0.1	(0.005 to 0.577)	0.063	-3.223	(-3.914 to -2.526)
TND-MLMD2	cg19592829	20	57426215	0.288	0.12	-0.464	-6.185	6.07E-08	6.07E-06	(3.67E-12 to 3.18E-05)	5.68E-05	-6.246	(-7.482 to -4.980)
BWS-MLMD4	cg20401058	20	57426240	-0.317	0.105	-0.965	-6.102	8.16E-08	8.16E-06	(7.79E-12 to 4.50E-05)	7.70E-05	-6.162	(-7.382 to -4.912)
TND-MLMD2	cg20401058	20	57426240	0.149	0.199	-1.383	-7.641	3.39E-10	3.39E-08	(1.34E-18 to 4.53E-08)	4.71E-07	-7.718	(-9.231 to -6.125)
BWS-MLMD4	cg11669839	20	57426322	-0.418	0.093	-0.81	-4.172	6.15E-05	0.00615	(1.82E-05 to 0.043)	0.011	-4.213	(-5.087 to -3.334)
TND-MLMD2	cg11669839	20	57426322	-0.011	0.129	-1.069	-8.146	5.73E-11	5.73E-09	(3.90E-21 to 3.23E-09)	9.03E-08	-8.227	(-9.837 to -6.533)
BWS-MLMD4	cg17841572	20	57426368	-0.311	0.094	-0.59	-2.939	0.003	0.3	(0.015 to 1.025)	0.091	-2.969	(-3.614 to -2.317)
TND-MLMD2	cg17841572	20	57426368	0.108	0.132	-1.04	-8.63	1.06E-11	1.06E-09	(1.02E-23 to 2.20E-10)	1.84E-08	-8.716	(-10.419 to -6.923)
BWS-MLMD4	cg08166863	20	57426391	0.116	0.134	-0.206	-2.383	0.011	1.1	(0.157 to 3.190)	0.179	-2.407	(-2.954 to -1.854)
TND-MLMD2	cg08166863	20	57426391	0.119	0.139	-0.572	-4.918	5.12E-06	0.000512	(1.23E-07 to 0.004)	0.002	-4.967	(-5.963 to -3.946)
BWS-MLMD4	cg06200857	20	57426420	0.125	0.329	-0.252	-1.137	0.131	13.1	(6.650 to 21.585)	0.418	-1.148	(-1.502 to -0.786)
TND-MLMD2	cg06200857	20	57426420	0.628	0.368	-1.009	-4.4	2.92E-05	0.00292	(4.56E-06 to 0.021)	0.007	-4.444	(-5.344 to -3.521)
BWS-MLMD4	cg08091561	20	57426425	-0.031	0.354	-0.356	-0.909	0.184	18.4	(10.647 to 27.987)	0.445	-0.918	(-1.246 to -0.583)
TND-MLMD2	cg08091561	20	57426425	0.48	0.391	-1.121	-4.06	8.83E-05	0.00883	(3.67E-05 to 0.059)	0.013	-4.1	(-4.952 to -3.242)
BWS-MLMD4	cg22860367	20	57426538	-0.343	0.09	-1.039	-7.648	3.31E-10	3.31E-08	(1.25E-18 to 4.38E-08)	5.67E-07	-7.724	(-9.239 to -6.130)

TND-MLMD2	cg22860367	20	57426538	0.185	0.131	-1.63	-13.743	0	0	(5.63E-60 to 1.13E-26)	0	-13.88	(-16.571 to -11.047)
BWS-MLMD4	cg07947033	20	57426545	0.016	0.268	-0.304	-1.183	0.121	12.1	(5.992 to 20.400)	0.411	-1.195	(-1.555 to -0.827)
TND-MLMD2	cg07947033	20	57426545	0.01	0.256	-1.445	-5.616	4.55E-07	4.55E-05	(5.26E-10 to 0.000)	0	-5.671	(-6.799 to -4.516)
BWS-MLMD4	cg06693667	20	57426570	0.033	0.087	-0.133	-1.884	0.033	3.3	(0.900 to 7.607)	0.28	-1.902	(-2.366 to -1.432)
TND-MLMD2	cg06693667	20	57426570	0.45	0.12	-0.832	-10.571	1.54E-14	1.54E-12	(1.48E-35 to 1.03E-15)	3.82E-11	-10.677	(-12.754 to -8.490)
BWS-MLMD4	cg15631127	20	57426580	0.129	0.113	-0.269	-3.489	0.001	0.1	(0.001 to 0.278)	0.039	-3.524	(-4.269 to -2.772)
TND-MLMD2	cg15631127	20	57426580	0.159	0.108	-0.419	-5.314	1.31E-06	0.000131	(6.09E-09 to 0.001)	0.001	-5.366	(-6.437 to -4.269)
BWS-MLMD4	cg23484981	20	57426626	-0.218	0.722	-0.826	-0.833	0.204	20.4	(12.272 to 30.323)	0.454	-0.842	(-1.161 to -0.515)
TND-MLMD2	cg23484981	20	57426626	0.155	0.359	-1.515	-4.606	1.47E-05	0.00147	(1.13E-06 to 0.011)	0.005	-4.652	(-5.590 to -3.690)
BWS-MLMD4	cg04677683	20	57426743	-0.106	0.207	-0.353	-1.184	0.121	12.1	(5.983 to 20.383)	0.41	-1.196	(-1.556 to -0.828)
TND-MLMD2	cg04677683	20	57426743	0.326	0.247	-1.512	-7.37	8.87E-10	8.87E-08	(2.66E-17 to 1.75E-07)	1.18E-06	-7.444	(-8.905 to -5.906)
BWS-MLMD4	cg15160445	20	57426749	0.255	0.26	0.023	-0.885	0.19	19	(11.137 to 28.704)	0.448	-0.894	(-1.219 to -0.562)
TND-MLMD2	cg15160445	20	57426749	0.706	0.243	-1.206	-7.804	1.91E-10	1.91E-08	(2.13E-19 to 1.97E-08)	2.76E-07	-7.881	(-9.426 to -6.256)
BWS-MLMD4	cg25326570	20	57426757	0.112	0.232	-0.465	-2.46	0.009	0.9	(0.117 to 2.759)	0.165	-2.484	(-3.044 to -1.918)
TND-MLMD2	cg25326570	20	57426757	0.543	0.22	-1.411	-8.781	6.26E-12	6.26E-10	(1.47E-24 to 9.22E-11)	1.12E-08	-8.869	(-10.601 to -7.046)
BWS-MLMD4	cg23249369	20	57426759	0.257	0.155	-0.458	-4.561	1.71E-05	0.00171	(1.55E-06 to 0.013)	0.005	-4.606	(-5.536 to -3.653)
TND-MLMD2	cg23249369	20	57426759	0.299	0.168	-1.794	-12.338	0	0	(2.22E-48 to 1.79E-21)	0	-12.461	(-14.880 to -9.915)
BWS-MLMD4	cg01715551	20	57426789	-0.185	0.094	-0.788	-6.345	3.43E-08	3.43E-06	(8.34E-13 to 1.60E-05)	3.70E-05	-6.408	(-7.674 to -5.111)
TND-MLMD2	cg01715551	20	57426789	0.174	0.13	-1.262	-10.954	4.44E-15	4.44E-13	(3.66E-38 to 6.94E-17)	1.20E-11	-11.062	(-13.214 to -8.798)
BWS-MLMD4	cg14176797	20	57426801	-0.073	0.084	-0.651	-6.845	5.77E-09	5.77E-07	(6.48E-15 to 2.11E-06)	7.42E-06	-6.913	(-8.274 to -5.481)
TND-MLMD2	cg14176797	20	57426801	0.367	0.125	-1.229	-12.596	0	0	(2.06E-50 to 2.20E-22)	0	-12.721	(-15.190 to -10.122)
BWS-MLMD4	cg04457481	20	57426835	0	0.093	-0.958	-10.154	6.04E-14	6.04E-12	(8.19E-33 to 1.77E-14)	1.91E-10	-10.255	(-12.252 to -8.154)
TND-MLMD2	cg04457481	20	57426835	0.309	0.135	-1.886	-16.15	0	0	(1.02E-82 to 7.33E-37)	0	-16.311	(-19.468 to -12.986)
BWS-MLMD4	cg21938532	20	57426931	0.102	0.143	-0.766	-6.023	1.08E-07	1.08E-05	(1.57E-11 to 6.22E-05)	9.68E-05	-6.083	(-7.288 to -4.848)
TND-MLMD2	cg21938532	20	57426931	0.119	0.154	-2.124	-14.416	0	0	(5.78E-66 to 2.34E-29)	0	-14.559	(-17.381 to -11.589)
BWS-MLMD4	cg03606258	20	57426935	-0.2	1.383	-0.706	-0.362	0.359	35.9	(25.777 to 46.916)	0.487	-0.366	(-0.650 to -0.077)
TND-MLMD2	cg03606258	20	57426935	0.344	1.171	-1.666	-1.7	0.048	4.8	(1.575 to 10.115)	0.293	-1.717	(-2.151 to -1.275)
BWS-MLMD4	cg22989942	20	57426950	0.16	0.118	-0.598	-6.347	3.41E-08	3.41E-06	(8.18E-13 to 1.59E-05)	3.68E-05	-6.41	(-7.676 to -5.112)
TND-MLMD2	cg22989942	20	57426950	0.294	0.135	-1.458	-12.812	0	0	(3.73E-52 to 3.64E-23)	0	-12.94	(-15.451 to -10.297)
BWS-MLMD4	cg14309385	20	57427010	0.098	0.114	-0.305	-3.51	0	0	(0.001 to 0.264)	0.037	-3.545	(-4.294 to -2.789)
TND-MLMD2	cg14309385	20	57427010	0.172	0.124	-0.986	-9.215	1.42E-12	1.42E-10	(4.90E-27 to 7.04E-12)	2.84E-09	-9.306	(-11.122 to -7.395)

BWS-MLMD4	cg05309239	20	57427017	-0.078	0.108	-0.65	-5.229	1.76E-06	0.000176	(1.18E-08 to 0.001)	0.001	-5.281	(-6.336 to -4.200)
TND-MLMD2	cg05309239	20	57427017	-0.017	0.158	-1.874	-11.608	5.55E-16	5.55E-14	(7.69E-43 to 5.50E-19)	1.70E-12	-11.723	(-14.001 to -9.326)
BWS-MLMD4	cg12573482	20	57427030	-0.306	0.09	-0.765	-5.035	3.43E-06	0.000343	(5.19E-08 to 0.003)	0.001	-5.085	(-6.104 to -4.041)
TND-MLMD2	cg12573482	20	57427030	0.183	0.122	-1.782	-15.982	0	0	(5.11E-81 to 4.23E-36)	0	-16.141	(-19.267 to -12.851)
BWS-MLMD4	cg06324048	20	57427103	0.035	0.129	-1.137	-8.983	3.12E-12	3.12E-10	(1.07E-25 to 2.82E-11)	7.09E-09	-9.073	(-10.844 to -7.209)
TND-MLMD2	cg06324048	20	57427103	0.453	0.207	-2.402	-13.629	0	0	(5.50E-59 to 3.14E-26)	0	-13.765	(-16.434 to -10.955)
BWS-MLMD4	cg13728472	20	57427117	-0.388	0.132	-1.027	-4.806	7.50E-06	0.00075	(2.78E-07 to 0.006)	0.003	-4.854	(-5.829 to -3.854)
TND-MLMD2	cg13728472	20	57427117	-0.285	0.194	-1.775	-7.6	3.92E-10	3.92E-08	(2.13E-18 to 5.58E-08)	5.41E-07	-7.676	(-9.182 to -6.092)
BWS-MLMD4	cg24617313	20	57427146	-0.192	0.625	-0.359	-0.265	0.396	39.6	(29.185 to 50.651)	0.491	-0.267	(-0.548 to 0.016)
TND-MLMD2	cg24617313	20	57427146	-0.09	0.593	-1.994	-3.18	0.001	0.1	(0.005 to 0.592)	0.052	-3.212	(-3.900 to -2.517)
BWS-MLMD4	cg03908391	20	57427170	0.306	0.174	-0.536	-4.781	8.16E-06	0.000816	(3.33E-07 to 0.006)	0.003	-4.829	(-5.799 to -3.833)
TND-MLMD2	cg03908391	20	57427170	0.269	0.224	-1.69	-8.66	9.51E-12	9.51E-10	(6.90E-24 to 1.85E-10)	1.67E-08	-8.747	(-10.456 to -6.948)
BWS-MLMD4	cg22290117	20	57427173	0.213	0.157	-0.544	-4.763	8.66E-06	0.000866	(3.78E-07 to 0.007)	0.003	-4.811	(-5.778 to -3.819)
TND-MLMD2	cg22290117	20	57427173	0.612	0.194	-1.151	-8.999	2.95E-12	2.95E-10	(8.63E-26 to 2.56E-11)	5.60E-09	-9.089	(-10.863 to -7.222)
BWS-MLMD4	cg26496204	20	57427210	0.011	0.132	-0.623	-4.759	8.80E-06	0.00088	(3.91E-07 to 0.007)	0.003	-4.806	(-5.772 to -3.815)
TND-MLMD2	cg26496204	20	57427210	0.46	0.169	-1.542	-11.745	4.44E-16	4.44E-14	(7.38E-44 to 1.92E-19)	1.37E-12	-11.862	(-14.166 to -9.437)
BWS-MLMD4	cg16833551	20	57427237	0.797	0.19	-0.179	-5.075	2.99E-06	0.000299	(3.85E-08 to 0.002)	0.001	-5.125	(-6.151 to -4.074)
TND-MLMD2	cg16833551	20	57427237	1.199	0.186	-1.433	-13.979	0	0	(4.84E-62 to 1.34E-27)	0	-14.118	(-16.855 to -11.237)
BWS-MLMD4	cg03010274	20	57427274	-0.403	0.114	-1.076	-5.861	1.92E-07	1.92E-05	(6.57E-11 to 0.000)	0	-5.919	(-7.093 to -4.716)
TND-MLMD2	cg03010274	20	57427274	0.009	0.173	-1.723	-9.922	1.30E-13	1.30E-11	(2.46E-31 to 8.16E-14)	2.91E-10	-10.021	(-11.973 to -7.967)
BWS-MLMD4	cg25652859	20	57427412	-0.814	0.191	-1.321	-2.626	0.006	0.6	(0.060 to 1.987)	0.136	-2.652	(-3.241 to -2.056)
TND-MLMD2	cg25652859	20	57427412	-0.412	0.314	-2.592	-6.872	5.24E-09	5.24E-07	(4.92E-15 to 1.86E-06)	6.14E-06	-6.94	(-8.307 to -5.503)
BWS-MLMD4	cg12321149	20	57427426	-0.724	0.184	-0.999	-1.478	0.073	7.3	(2.918 to 13.919)	0.361	-1.492	(-1.893 to -1.084)
TND-MLMD2	cg12321149	20	57427426	-0.318	0.247	-2.161	-7.398	8.04E-10	8.04E-08	(1.97E-17 to 1.53E-07)	1.08E-06	-7.472	(-8.939 to -5.929)
BWS-MLMD4	cg27304369	20	57427483	-0.314	0.215	-1.209	-4.118	7.32E-05	0.00732	(2.55E-05 to 0.050)	0.012	-4.159	(-5.022 to -3.290)
TND-MLMD2	cg27304369	20	57427483	-0.32	0.244	-2.749	-9.874	1.53E-13	1.53E-11	(4.91E-31 to 1.11E-13)	3.38E-10	-9.973	(-11.916 to -7.928)
BWS-MLMD4	cg26811638	20	57427493	-0.522	0.178	-1.234	-3.967	0	0	(6.45E-05 to 0.077)	0.016	-4.006	(-4.841 to -3.166)
TND-MLMD2	cg26811638	20	57427493	-0.452	0.251	-2.256	-7.105	2.28E-09	2.28E-07	(4.48E-16 to 6.29E-07)	2.85E-06	-7.176	(-8.586 to -5.692)
BWS-MLMD4	cg26534489	20	57427495	-0.318	0.149	-0.794	-3.174	0.001	0.1	(0.005 to 0.601)	0.065	-3.205	(-3.893 to -2.512)
TND-MLMD2	cg26534489	20	57427495	-0.287	0.188	-1.464	-6.212	5.51E-08	5.51E-06	(2.86E-12 to 2.83E-05)	5.23E-05	-6.274	(-7.514 to -5.002)
BWS-MLMD4	cg08587534	20	57427503	-0.02	0.163	-0.653	-3.837	0	0	(0.000 to 0.111)	0.021	-3.875	(-4.686 to -3.059)

TND-MLMD2	cg08587534	20	57427503	0.013	0.174	-1.671	-9.572	4.21E-13	4.21E-11	(3.62E-29 to 7.72E-13)	8.88E-10	-9.667	(-11.552 to -7.684)
BWS-MLMD4	cg04257105	20	57427713	-0.346	0.19	-0.909	-2.931	0.003	0.3	(0.016 to 1.044)	0.092	-2.96	(-3.603 to -2.310)
TND-MLMD2	cg04257105	20	57427713	-0.26	0.244	-1.756	-6.073	9.03E-08	9.03E-06	(1.01E-11 to 5.07E-05)	7.93E-05	-6.133	(-7.348 to -4.889)
BWS-MLMD4	cg20528838	20	57427730	-0.959	0.196	-1.43	-2.378	0.011	1.1	(0.160 to 3.221)	0.18	-2.402	(-2.948 to -1.849)
TND-MLMD2	cg20528838	20	57427730	-0.868	0.272	-2.556	-6.139	7.13E-08	7.13E-06	(5.54E-12 to 3.84E-05)	6.48E-05	-6.201	(-7.427 to -4.943)
BWS-MLMD4	cg27661264	20	57427738	-0.979	0.186	-1.413	-2.309	0.013	1.3	(0.208 to 3.661)	0.193	-2.332	(-2.866 to -1.791)
TND-MLMD2	cg27661264	20	57427738	-0.579	0.267	-2.315	-6.436	2.48E-08	2.48E-06	(3.55E-13 to 1.08E-05)	2.55E-05	-6.5	(-7.783 to -5.185)
BWS-MLMD4	cg19589727	20	57427762	-0.935	0.171	-1.203	-1.555	0.063	6.3	(2.372 to 12.494)	0.346	-1.57	(-1.982 to -1.151)
TND-MLMD2	cg19589727	20	57427762	-0.578	0.252	-2.209	-6.399	2.84E-08	2.84E-06	(5.04E-13 to 1.27E-05)	2.86E-05	-6.462	(-7.738 to -5.155)
BWS-MLMD4	cg10302550	20	57427821	-0.286	0.168	-1.025	-4.348	3.46E-05	0.00346	(5.84E-06 to 0.025)	0.007	-4.391	(-5.298 to -3.479)
TND-MLMD2	cg10302550	20	57427821	-0.239	0.24	-2.109	-7.722	2.55E-10	2.55E-08	(5.39E-19 to 3.00E-08)	3.64E-07	-7.799	(-9.328 to -6.190)
BWS-MLMD4	cg17414107	20	57427830	-0.201	0.21	-0.864	-3.122	0.002	0.2	(0.006 to 0.677)	0.07	-3.154	(-3.832 to -2.469)
TND-MLMD2	cg17414107	20	57427830	0.244	0.306	-2.334	-8.337	2.93E-11	2.93E-09	(3.88E-22 to 1.14E-09)	4.81E-08	-8.42	(-10.067 to -6.687)
BWS-MLMD4	cg00943909	20	57427942	-0.286	0.177	-1.012	-4.071	8.52E-05	0.00852	(3.42E-05 to 0.058)	0.013	-4.111	(-4.966 to -3.251)
TND-MLMD2	cg00943909	20	57427942	-0.181	0.247	-2.137	-7.825	1.77E-10	1.77E-08	(1.68E-19 to 1.77E-08)	2.57E-07	-7.902	(-9.451 to -6.273)
BWS-MLMD4	cg19640589	20	57427973	-0.135	0.21	-0.848	-3.359	0.001	0.1	(0.002 to 0.386)	0.048	-3.392	(-4.113 to -2.664)
TND-MLMD2	cg19640589	20	57427973	-0.046	0.258	-2.332	-8.786	6.17E-12	6.17E-10	(1.39E-24 to 8.99E-11)	1.11E-08	-8.873	(-10.606 to -7.049)
BWS-MLMD4	cg24346429	20	57428032	-0.336	0.21	-0.543	-0.978	0.166	16.6	(9.287 to 25.933)	0.437	-0.988	(-1.323 to -0.645)
TND-MLMD2	cg24346429	20	57428032	0.198	0.207	-0.112	-1.481	0.072	7.2	(2.889 to 13.846)	0.338	-1.496	(-1.897 to -1.087)
BWS-MLMD4	cg24058407	20	57428282	1.482	0.236	1.387	-0.398	0.346	34.6	(24.546 to 45.535)	0.485	-0.402	(-0.689 to -0.112)
TND-MLMD2	cg24058407	20	57428282	1.569	0.142	1.234	-2.339	0.012	1.2	(0.186 to 3.468)	0.161	-2.362	(-2.901 to -1.816)
BWS-MLMD4	cg21809160	20	57428309	2.789	0.183	2.737	-0.276	0.392	39.2	(28.760 to 50.192)	0.491	-0.279	(-0.560 to 0.005)
TND-MLMD2	cg21809160	20	57428309	2.834	0.127	2.505	-2.566	0.007	0.7	(0.076 to 2.243)	0.122	-2.591	(-3.170 to -2.006)
BWS-MLMD4	cg09583957	20	57428315	1.947	0.327	2.24	0.887	0.19	19	(11.088 to 28.634)	0.448	0.896	(0.564 to 1.222)
TND-MLMD2	cg09583957	20	57428315	2.02	0.242	1.56	-1.883	0.033	3.3	(0.901 to 7.613)	0.254	-1.902	(-2.365 to -1.432)
BWS-MLMD4	cg25367568	20	57428437	1.121	0.188	0.605	-2.717	0.005	0.5	(0.040 to 1.648)	0.121	-2.744	(-3.350 to -2.133)
TND-MLMD2	cg25367568	20	57428437	1.361	0.161	1.138	-1.375	0.088	8.8	(3.800 to 15.992)	0.359	-1.388	(-1.774 to -0.995)
BWS-MLMD4	cg21988465	20	57429277	0.174	0.144	-0.643	-5.622	4.45E-07	4.45E-05	(4.98E-10 to 0.000)	0	-5.678	(-6.807 to -4.521)
TND-MLMD2	cg21988465	20	57429277	0.326	0.18	-2.352	-14.713	0	0	(1.05E-68 to 1.39E-30)	0	-14.86	(-17.739 to -11.829)
BWS-MLMD4	cg07284407	20	57429858	-0.007	0.133	-0.594	-4.384	3.08E-05	0.00308	(5.07E-06 to 0.023)	0.007	-4.427	(-5.324 to -3.508)
TND-MLMD2	cg07284407	20	57429858	0.434	0.164	-1.774	-13.331	0	0	(1.90E-56 to 4.32E-25)	0	-13.464	(-16.075 to -10.715)

BWS-MLMD4	cg21625881	20	57430313	0.281	0.113	-0.153	-3.797	0	0	(0.000 to 0.124)	0.023	-3.835	(-4.638 to -3.026)
TND-MLMD2	cg21625881	20	57430313	0.518	0.134	-1.171	-12.526	0	0	(7.44E-50 to 3.91E-22)	0	-12.65	(-15.106 to -10.066)
BWS-MLMD4	cg14203179	20	57430663	0.01	0.125	-0.404	-3.277	0.001	0.1	(0.003 to 0.470)	0.055	-3.31	(-4.016 to -2.597)
TND-MLMD2	cg14203179	20	57430663	0.038	0.176	-1.608	-9.282	1.13E-12	1.13E-10	(1.98E-27 to 4.68E-12)	2.27E-09	-9.374	(-11.203 to -7.450)
BWS-MLMD4	cg09437522	20	57431202	-0.49	0.117	-0.944	-3.83	0	0	(0.000 to 0.113)	0.021	-3.868	(-4.677 to -3.053)
TND-MLMD2	cg09437522	20	57431202	0.003	0.157	-2.196	-13.88	0	0	(3.62E-61 to 3.31E-27)	0	-14.018	(-16.735 to -11.157)
BWS-MLMD4	cg10468484	20	57431303	-0.17	0.117	-0.8	-5.324	1.26E-06	0.000126	(5.61E-09 to 0.001)	0.001	-5.377	(-6.450 to -4.277)
TND-MLMD2	cg10468484	20	57431303	-0.001	0.145	-2.068	-14.095	0	0	(4.46E-63 to 4.60E-28)	0	-14.236	(-16.995 to -11.331)
BWS-MLMD4	cg15122327	20	57435146	2.563	0.294	2.67	0.361	0.36	36	(25.793 to 46.933)	0.487	0.365	(0.077 to 0.650)
TND-MLMD2	cg15122327	20	57435146	2.692	0.198	3.108	2.078	0.021	2.1	(0.475 to 5.520)	0.212	2.098	(1.596 to 2.593)
BWS-MLMD4	cg10797197	20	57444000	0.081	0.184	-0.196	-1.494	0.071	7.1	(2.796 to 13.612)	0.357	-1.509	(-1.912 to -1.098)
TND-MLMD2	cg10797197	20	57444000	0.065	0.181	0.258	1.052	0.149	14.9	(7.982 to 23.852)	0.413	1.062	(0.711 to 1.406)
BWS-MLMD4	cg01565918	20	57457492	3.445	0.275	3.637	0.692	0.246	24.6	(15.740 to 34.975)	0.466	0.698	(0.386 to 1.005)
TND-MLMD2	cg01565918	20	57457492	3.344	0.163	3.54	1.186	0.121	12.1	(5.957 to 20.336)	0.392	1.198	(0.830 to 1.558)
BWS-MLMD4	cg27484541	20	57461542	2.983	0.293	3.038	0.186	0.426	42.6	(32.024 to 53.677)	0.494	0.188	(-0.092 to 0.467)
TND-MLMD2	cg27484541	20	57461542	3.012	0.213	3.187	0.815	0.21	21	(12.690 to 30.905)	0.442	0.823	(0.499 to 1.141)
BWS-MLMD4	cg17658854	20	57462798	2.046	0.201	2.133	0.43	0.335	33.5	(23.502 to 44.349)	0.484	0.434	(0.142 to 0.722)
TND-MLMD2	cg17658854	20	57462798	2.22	0.126	2.133	-0.683	0.249	24.9	(15.961 to 35.259)	0.457	-0.69	(-0.996 to -0.378)
BWS-MLMD4	cg20782596	20	57462978	2.734	0.159	2.366	-2.294	0.013	1.3	(0.220 to 3.765)	0.196	-2.317	(-2.848 to -1.779)
TND-MLMD2	cg20782596	20	57462978	2.704	0.155	2.614	-0.577	0.283	28.3	(18.931 to 38.967)	0.466	-0.583	(-0.880 to -0.280)
BWS-MLMD4	cg24276988	20	57463106	2.373	0.252	2.084	-1.136	0.131	13.1	(6.658 to 21.600)	0.418	-1.147	(-1.502 to -0.786)
TND-MLMD2	cg24276988	20	57463106	2.499	0.285	2.191	-1.071	0.145	14.5	(7.664 to 23.325)	0.41	-1.082	(-1.428 to -0.728)
BWS-MLMD4	cg22741626	20	57463265	0.781	0.204	0.43	-1.705	0.047	4.7	(1.549 to 10.027)	0.316	-1.722	(-2.157 to -1.280)
TND-MLMD2	cg22741626	20	57463265	1.157	0.156	0.356	-5.081	2.93E-06	0.000293	(3.68E-08 to 0.002)	0.001	-5.131	(-6.158 to -4.079)
BWS-MLMD4	cg18997188	20	57463270	0.434	0.17	0.075	-2.092	0.021	2.1	(0.453 to 5.386)	0.236	-2.113	(-2.610 to -1.609)
TND-MLMD2	cg18997188	20	57463270	0.931	0.169	-0.092	-5.986	1.23E-07	1.23E-05	(2.19E-11 to 7.25E-05)	0	-6.046	(-7.243 to -4.818)
BWS-MLMD4	cg20213508	20	57463325	0.872	0.18	0.705	-0.916	0.182	18.2	(10.496 to 27.765)	0.444	-0.925	(-1.254 to -0.590)
TND-MLMD2	cg20213508	20	57463325	1.251	0.167	0.391	-5.112	2.63E-06	0.000263	(2.91E-08 to 0.002)	0.001	-5.162	(-6.195 to -4.104)
BWS-MLMD4	cg04779428	20	57463355	0.545	0.194	0.329	-1.101	0.138	13.8	(7.192 to 22.527)	0.422	-1.112	(-1.462 to -0.755)
TND-MLMD2	cg04779428	20	57463355	0.968	0.181	0.002	-5.273	1.51E-06	0.000151	(8.40E-09 to 0.001)	0.001	-5.325	(-6.388 to -4.236)
BWS-MLMD4	cg04019914	20	57463357	0.805	0.192	0.934	0.668	0.254	25.4	(16.373 to 35.786)	0.468	0.675	(0.364 to 0.979)

TND-MLMD2	cg04019914	20	57463357	1.224	0.144	0.603	-4.258	4.65E-05	0.00465	(1.05E-05 to 0.033)	0.009	-4.3	(-5.190 to -3.404)
BWS-MLMD4	cg11357538	20	57463397	0.043	0.092	-0.441	-5.198	1.95E-06	0.000195	(1.50E-08 to 0.001)	0.001	-5.25	(-6.299 to -4.175)
TND-MLMD2	cg11357538	20	57463397	0.437	0.111	-1.335	-15.741	0	0	(1.33E-78 to 5.12E-35)	0	-15.898	(-18.977 to -12.657)
BWS-MLMD4	cg20008140	20	57463455	-0.073	0.201	-0.291	-1.074	0.144	14.4	(7.614 to 23.241)	0.426	-1.085	(-1.432 to -0.731)
TND-MLMD2	cg20008140	20	57463455	0.376	0.245	-0.866	-5.016	3.66E-06	0.000366	(5.97E-08 to 0.003)	0.002	-5.066	(-6.081 to -4.026)
BWS-MLMD4	cg11480267	20	57463503	-0.072	0.238	-0.458	-1.608	0.057	5.7	(2.050 to 11.584)	0.335	-1.624	(-2.044 to -1.196)
TND-MLMD2	cg11480267	20	57463503	0.251	0.345	-1.392	-4.719	1.01E-05	0.00101	(5.19E-07 to 0.008)	0.004	-4.765	(-5.725 to -3.782)
BWS-MLMD4	cg10011623	20	57463527	-0.53	0.107	-0.938	-3.791	0	0	(0.000 to 0.126)	0.023	-3.828	(-4.630 to -3.021)
TND-MLMD2	cg10011623	20	57463527	-0.027	0.208	-1.466	-6.846	5.74E-09	5.74E-07	(6.39E-15 to 2.10E-06)	6.67E-06	-6.914	(-8.276 to -5.482)
BWS-MLMD4	cg01748573	20	57463530	-0.259	0.14	-0.764	-3.582	0	0	(0.001 to 0.219)	0.033	-3.618	(-4.381 to -2.849)
TND-MLMD2	cg01748573	20	57463530	0.219	0.227	-1.682	-8.296	3.38E-11	3.38E-09	(6.39E-22 to 1.43E-09)	5.51E-08	-8.378	(-10.017 to -6.654)
BWS-MLMD4	cg17334845	20	57463572	-0.227	0.096	-0.628	-4.138	6.86E-05	0.00686	(2.25E-05 to 0.047)	0.011	-4.179	(-5.046 to -3.306)
TND-MLMD2	cg17334845	20	57463572	0.131	0.146	-1.939	-14.04	0	0	(1.38E-62 to 7.63E-28)	0	-14.18	(-16.929 to -11.287)
BWS-MLMD4	cg26767990	20	57463615	-0.103	0.114	-0.568	-4.026	9.83E-05	0.00983	(4.51E-05 to 0.065)	0.014	-4.066	(-4.912 to -3.214)
TND-MLMD2	cg26767990	20	57463615	-0.182	0.155	-1.179	-6.354	3.32E-08	3.32E-06	(7.63E-13 to 1.54E-05)	3.30E-05	-6.418	(-7.685 to -5.118)
BWS-MLMD4	cg17652507	20	57463653	-0.376	0.155	-0.914	-3.441	0.001	0.1	(0.001 to 0.314)	0.042	-3.476	(-4.212 to -2.733)
TND-MLMD2	cg17652507	20	57463653	-0.141	0.211	-2.131	-9.34	9.22E-13	9.22E-11	(8.89E-28 to 3.26E-12)	1.88E-09	-9.433	(-11.273 to -7.497)
BWS-MLMD4	cg22407822	20	57463658	-0.164	0.157	-0.768	-3.802	0	0	(0.000 to 0.122)	0.022	-3.84	(-4.644 to -3.030)
TND-MLMD2	cg22407822	20	57463658	-0.052	0.199	-1.7	-8.199	4.75E-11	4.75E-09	(2.06E-21 to 2.42E-09)	7.65E-08	-8.281	(-9.901 to -6.576)
BWS-MLMD4	cg07341934	20	57463711	-0.146	0.119	-0.505	-2.996	0.002	0.2	(0.012 to 0.903)	0.084	-3.026	(-3.681 to -2.364)
TND-MLMD2	cg07341934	20	57463711	0.466	0.194	-1.338	-9.198	1.50E-12	1.50E-10	(6.10E-27 to 7.77E-12)	2.94E-09	-9.29	(-11.102 to -7.382)
BWS-MLMD4	cg23496597	20	57463725	-0.104	0.174	-0.652	-3.12	0.002	0.2	(0.006 to 0.681)	0.07	-3.151	(-3.829 to -2.467)
TND-MLMD2	cg23496597	20	57463725	0.021	0.184	-1.478	-8.085	7.10E-11	7.10E-09	(8.08E-21 to 4.49E-09)	1.10E-07	-8.165	(-9.763 to -6.483)
BWS-MLMD4	cg25308079	20	57463763	0.201	0.111	-0.249	-3.997	0	0	(5.37E-05 to 0.071)	0.015	-4.037	(-4.878 to -3.191)
TND-MLMD2	cg25308079	20	57463763	0.227	0.128	-0.626	-6.58	1.49E-08	1.49E-06	(8.90E-14 to 5.72E-06)	1.59E-05	-6.645	(-7.956 to -5.302)
BWS-MLMD4	cg03014008	20	57463767	-0.242	0.164	-0.783	-3.271	0.001	0.1	(0.003 to 0.478)	0.055	-3.303	(-4.009 to -2.592)
BWS-MLMD4	cg09772382	20	57463775	0.383	0.132	-0.067	-3.375	0.001	0.1	(0.002 to 0.370)	0.047	-3.409	(-4.134 to -2.678)
TND-MLMD2	cg09772382	20	57463775	0.371	0.11	-0.526	-8.056	7.86E-11	7.86E-09	(1.14E-20 to 5.24E-09)	1.21E-07	-8.136	(-9.729 to -6.460)
BWS-MLMD4	cg15222215	20	57463783	0.046	0.132	-0.441	-3.662	0	0	(0.000 to 0.178)	0.029	-3.699	(-4.476 to -2.915)
TND-MLMD2	cg15222215	20	57463783	0.095	0.174	-1.332	-8.131	6.03E-11	6.03E-09	(4.65E-21 to 3.50E-09)	9.44E-08	-8.212	(-9.819 to -6.521)
BWS-MLMD4	cg11244758	20	57463900	-0.036	0.132	-0.403	-2.745	0.004	0.4	(0.036 to 1.557)	0.117	-2.772	(-3.382 to -2.155)

TND-MLMD2	cg11244758	20	57463900	0.129	0.147	-0.85	-6.601	1.38E-08	1.38E-06	(7.24E-14 to 5.20E-06)	1.49E-05	-6.667	(-7.981 to -5.319)
BWS-MLMD4	cg18160880	20	57463903	0.587	0.134	0.077	-3.78	0	0	(0.000 to 0.130)	0.023	-3.817	(-4.617 to -3.012)
TND-MLMD2	cg18160880	20	57463903	0.731	0.147	-0.715	-9.721	2.55E-13	2.55E-11	(4.42E-30 to 3.00E-13)	5.51E-10	-9.818	(-11.731 to -7.804)
BWS-MLMD4	cg05926269	20	57463906	0.688	0.141	0.292	-2.778	0.004	0.4	(0.031 to 1.451)	0.112	-2.806	(-3.422 to -2.183)
TND-MLMD2	cg05926269	20	57463906	0.793	0.148	-0.703	-10.008	9.79E-14	9.79E-12	(7.03E-32 to 4.65E-14)	2.23E-10	-10.108	(-12.076 to -8.036)
BWS-MLMD4	cg03821543	20	57463925	-0.018	0.171	-0.587	-3.287	0.001	0.1	(0.003 to 0.459)	0.054	-3.32	(-4.028 to -2.605)
TND-MLMD2	cg03821543	20	57463925	0.022	0.179	-1.618	-9.074	2.29E-12	2.29E-10	(3.21E-26 to 1.64E-11)	4.37E-09	-9.164	(-10.953 to -7.282)
BWS-MLMD4	cg01538522	20	57463974	-0.632	0.129	-0.842	-1.607	0.057	5.7	(2.054 to 11.597)	0.335	-1.623	(-2.043 to -1.195)
TND-MLMD2	cg01538522	20	57463974	-0.1	0.199	-1.653	-7.729	2.49E-10	2.49E-08	(5.01E-19 to 2.90E-08)	3.56E-07	-7.806	(-9.336 to -6.196)
BWS-MLMD4	cg00267746	20	57463984	-0.284	0.162	-0.592	-1.879	0.033	3.3	(0.914 to 7.665)	0.281	-1.898	(-2.360 to -1.428)
TND-MLMD2	cg00267746	20	57463984	-0.207	0.183	-1.554	-7.298	1.15E-09	1.15E-07	(5.80E-17 to 2.49E-07)	1.49E-06	-7.371	(-8.819 to -5.848)
BWS-MLMD4	cg09885502	20	57463991	-0.12	1.632	-0.437	-0.192	0.424	42.4	(31.812 to 53.453)	0.494	-0.194	(-0.473 to 0.087)
TND-MLMD2	cg09885502	20	57463991	0.119	1.812	-0.872	-0.542	0.295	29.5	(19.987 to 40.241)	0.469	-0.547	(-0.842 to -0.247)
BWS-MLMD4	cg03837903	20	57464000	0.152	0.254	-0.102	-0.988	0.164	16.4	(9.099 to 25.640)	0.436	-0.998	(-1.335 to -0.654)
TND-MLMD2	cg03837903	20	57464000	0.675	0.315	-0.784	-4.59	1.55E-05	0.00155	(1.27E-06 to 0.012)	0.005	-4.635	(-5.570 to -3.677)
BWS-MLMD4	cg23159236	20	57464002	-0.617	0.152	-0.85	-1.522	0.067	6.7	(2.592 to 13.084)	0.352	-1.537	(-1.944 to -1.122)
TND-MLMD2	cg23159236	20	57464002	-0.417	0.199	-1.617	-5.97	1.30E-07	1.30E-05	(2.52E-11 to 7.74E-05)	0	-6.029	(-7.224 to -4.805)
BWS-MLMD4	cg22798925	20	57464129	0.003	0.14	-0.407	-2.909	0.003	0.3	(0.017 to 1.095)	0.094	-2.938	(-3.577 to -2.292)
TND-MLMD2	cg22798925	20	57464129	0.177	0.17	-1.104	-7.445	6.80E-10	6.80E-08	(1.18E-17 to 1.21E-07)	9.19E-07	-7.519	(-8.995 to -5.966)
BWS-MLMD4	cg20126878	20	57464571	-0.235	0.094	-0.64	-4.247	4.82E-05	0.00482	(1.13E-05 to 0.034)	0.009	-4.289	(-5.177 to -3.396)
TND-MLMD2	cg20126878	20	57464571	0.273	0.162	-1.61	-11.492	8.88E-16	8.88E-14	(5.36E-42 to 1.32E-18)	2.66E-12	-11.607	(-13.862 to -9.233)
BWS-MLMD4	cg27027803	20	57464742	0.173	0.089	-0.313	-5.423	8.94E-07	8.94E-05	(2.55E-09 to 0.001)	0.001	-5.477	(-6.568 to -4.358)
TND-MLMD2	cg27027803	20	57464742	0.635	0.173	-1.505	-12.258	0	0	(9.38E-48 to 3.43E-21)	0	-12.38	(-14.783 to -9.850)
BWS-MLMD4	cg08997444	20	57464970	0.581	0.125	0.178	-3.193	0.001	0.1	(0.005 to 0.575)	0.063	-3.224	(-3.915 to -2.527)
TND-MLMD2	cg08997444	20	57464970	0.86	0.169	-1.021	-11.051	3.33E-15	3.33E-13	(7.62E-39 to 3.43E-17)	9.37E-12	-11.161	(-13.331 to -8.877)
BWS-MLMD4	cg22639787	20	57464973	0.814	0.149	0.315	-3.325	0.001	0.1	(0.002 to 0.419)	0.051	-3.358	(-4.074 to -2.637)
TND-MLMD2	cg22639787	20	57464973	0.785	0.15	-0.847	-10.798	7.33E-15	7.33E-13	(4.31E-37 to 2.10E-16)	1.92E-11	-10.905	(-13.027 to -8.673)
BWS-MLMD4	cg05960039	20	57465123	-0.685	0.129	-1.005	-2.459	0.009	0.9	(0.117 to 2.760)	0.165	-2.484	(-3.044 to -1.917)
TND-MLMD2	cg05960039	20	57465123	-0.416	0.171	-1.828	-8.197	4.78E-11	4.78E-09	(2.11E-21 to 2.45E-09)	7.68E-08	-8.279	(-9.899 to -6.574)
BWS-MLMD4	cg09604333	20	57465125	-0.213	0.104	-0.6	-3.677	0	0	(0.000 to 0.171)	0.028	-3.713	(-4.494 to -2.927)
TND-MLMD2	cg09604333	20	57465125	-0.091	0.13	-1.1	-7.656	3.22E-10	3.22E-08	(1.14E-18 to 4.21E-08)	4.49E-07	-7.732	(-9.248 to -6.137)

BWS-MLMD4	cg06047881	20	57465132	-0.508	0.144	-0.738	-1.583	0.06	6	(2.195 to 12.002)	0.34	-1.599	(-2.015 to -1.175)
TND-MLMD2	cg06047881	20	57465132	0.056	0.163	-1.535	-9.666	3.07E-13	3.07E-11	(9.61E-30 to 4.25E-13)	6.55E-10	-9.762	(-11.665 to -7.760)
BWS-MLMD4	cg20018057	20	57465139	-0.368	0.142	-0.863	-3.459	0.001	0.1	(0.001 to 0.301)	0.041	-3.493	(-4.233 to -2.747)
TND-MLMD2	cg20018057	20	57465139	0.097	0.153	-1.543	-10.644	1.22E-14	1.22E-12	(4.78E-36 to 6.20E-16)	3.11E-11	-10.75	(-12.842 to -8.549)
BWS-MLMD4	cg10748817	20	57465175	0.026	0.164	-0.576	-3.639	0	0	(0.000 to 0.189)	0.03	-3.676	(-4.449 to -2.896)
TND-MLMD2	cg10748817	20	57465175	0.057	0.206	-1.513	-7.556	4.59E-10	4.59E-08	(3.48E-18 to 6.97E-08)	6.28E-07	-7.631	(-9.128 to -6.056)
BWS-MLMD4	cg25983380	20	57465439	-1.253	0.377	-2.104	-2.238	0.015	1.5	(0.270 to 4.170)	0.208	-2.26	(-2.782 to -1.731)
TND-MLMD2	cg25983380	20	57465439	-1.211	0.515	-2.949	-3.345	0.001	0.1	(0.002 to 0.399)	0.041	-3.378	(-4.097 to -2.653)
BWS-MLMD4	cg07909402	20	57465445	-1.719	0.369	-2.371	-1.748	0.043	4.3	(1.364 to 9.396)	0.307	-1.766	(-2.208 to -1.317)
TND-MLMD2	cg07909402	20	57465445	-1.59	0.515	-3.738	-4.134	6.95E-05	0.00695	(2.31E-05 to 0.048)	0.011	-4.175	(-5.042 to -3.303)
BWS-MLMD4	cg03264550	20	57465448	-1.038	0.289	-1.697	-2.26	0.014	1.4	(0.249 to 4.008)	0.204	-2.282	(-2.808 to -1.750)
TND-MLMD2	cg03264550	20	57465448	-0.879	0.367	-2.435	-4.2	5.62E-05	0.00562	(1.52E-05 to 0.039)	0.01	-4.242	(-5.121 to -3.357)
BWS-MLMD4	cg14104369	20	57465535	-2.264	0.175	-2.416	-0.858	0.198	19.8	(11.722 to 29.545)	0.451	-0.867	(-1.189 to -0.538)
TND-MLMD2	cg14104369	20	57465535	-2.048	0.211	-1.91	0.647	0.26	26	(16.929 to 36.490)	0.46	0.654	(0.345 to 0.957)
BWS-MLMD4	cg09241929	20	57465560	-3.419	0.295	-3.184	0.79	0.217	21.7	(13.278 to 31.714)	0.458	0.798	(0.476 to 1.113)
TND-MLMD2	cg09241929	20	57465560	-3.395	0.289	-3.253	0.487	0.314	31.4	(21.673 to 42.235)	0.473	0.492	(0.196 to 0.783)
BWS-MLMD4	cg10144604	20	57465599	-3.201	0.198	-3.275	-0.368	0.357	35.7	(25.556 to 46.669)	0.487	-0.372	(-0.657 to -0.084)
TND-MLMD2	cg10144604	20	57465599	-3.06	0.176	-3.022	0.213	0.416	41.6	(31.035 to 52.631)	0.491	0.216	(-0.066 to 0.495)
BWS-MLMD4	cg00466827	20	57465691	-3.217	0.206	-3.452	-1.131	0.132	13.2	(6.733 to 21.731)	0.418	-1.142	(-1.496 to -0.781)
TND-MLMD2	cg00466827	20	57465691	-3.178	0.158	-3.175	0.02	0.492	49.2	(38.294 to 60.128)	0.499	0.021	(-0.257 to 0.298)
BWS-MLMD4	cg18300848	20	57465695	-3.594	0.227	-3.935	-1.491	0.071	7.1	(2.819 to 13.669)	0.358	-1.505	(-1.908 to -1.095)
TND-MLMD2	cg18300848	20	57465695	-3.513	0.229	-3.304	0.905	0.185	18.5	(10.717 to 28.091)	0.432	0.914	(0.580 to 1.242)
BWS-MLMD4	cg07516978	20	57465773	-3.596	0.324	-3.708	-0.34	0.368	36.8	(26.530 to 47.752)	0.488	-0.343	(-0.627 to -0.056)
BWS-MLMD4	cg07824914	20	57465815	-3.494	0.171	-3.327	0.965	0.17	17	(9.539 to 26.322)	0.439	0.974	(0.633 to 1.308)
TND-MLMD2	cg07824914	20	57465815	-3.541	0.192	-3.678	-0.711	0.24	24	(15.223 to 34.304)	0.454	-0.718	(-1.027 to -0.404)
BWS-MLMD4	cg23143233	20	57465864	-3.503	0.224	-3.371	0.585	0.281	28.1	(18.715 to 38.703)	0.474	0.59	(0.287 to 0.888)
TND-MLMD2	cg23143233	20	57465864	-3.568	0.234	-3.911	-1.45	0.077	7.7	(3.134 to 14.450)	0.344	-1.465	(-1.861 to -1.060)
BWS-MLMD4	cg12372477	20	57465915	-3.401	0.158	-3.258	0.894	0.188	18.8	(10.940 to 28.418)	0.447	0.903	(0.570 to 1.230)
TND-MLMD2	cg12372477	20	57465915	-3.598	0.202	-3.551	0.231	0.409	40.9	(30.403 to 51.958)	0.49	0.233	(-0.049 to 0.513)
BWS-MLMD4	cg22885821	20	57465921	-3.244	0.125	-3.092	1.202	0.118	11.8	(5.748 to 19.948)	0.408	1.214	(0.843 to 1.576)
TND-MLMD2	cg22885821	20	57465921	-3.196	0.102	-3.228	-0.313	0.378	37.8	(27.471 to 48.788)	0.485	-0.316	(-0.599 to -0.030)

BWS-MLMD4	cg21222634	20	57465926	-3.53	0.323	-3.707	-0.544	0.294	29.4	(19.919 to 40.159)	0.477	-0.549	(-0.845 to -0.249)
TND-MLMD2	cg21222634	20	57465926	-3.612	0.144	-3.738	-0.865	0.196	19.6	(11.565 to 29.321)	0.437	-0.874	(-1.197 to -0.544)
BWS-MLMD4	cg19002779	20	57465993	-3.109	0.225	-3.168	-0.258	0.399	39.9	(29.423 to 50.907)	0.491	-0.26	(-0.541 to 0.023)
TND-MLMD2	cg19002779	20	57465993	-2.798	0.186	-2.791	0.035	0.486	48.6	(37.732 to 59.562)	0.499	0.035	(-0.242 to 0.313)
BWS-MLMD4	cg16993684	20	57466131	-4.393	0.493	-4.483	-0.18	0.429	42.9	(32.266 to 53.931)	0.494	-0.182	(-0.460 to 0.099)
BWS-MLMD4	cg18668503	20	57466137	-3.003	0.372	-2.746	0.684	0.249	24.9	(15.945 to 35.238)	0.467	0.691	(0.379 to 0.997)
TND-MLMD2	cg18668503	20	57466137	-2.864	0.29	-3.262	-1.358	0.09	9	(3.962 to 16.351)	0.362	-1.371	(-1.755 to -0.980)
BWS-MLMD4	cg09598225	20	57466839	-3.417	0.13	-3.308	0.828	0.206	20.6	(12.380 to 30.474)	0.454	0.837	(0.511 to 1.156)
TND-MLMD2	cg09598225	20	57466839	-3.363	0.125	-3.418	-0.435	0.333	33.3	(23.325 to 44.147)	0.477	-0.44	(-0.728 to -0.147)
BWS-MLMD4	cg10003667	20	57466900	-3.289	0.27	-3.503	-0.786	0.218	21.8	(13.375 to 31.846)	0.458	-0.794	(-1.109 to -0.472)
TND-MLMD2	cg10003667	20	57466900	-3.132	0.219	-2.922	0.951	0.173	17.3	(9.796 to 26.714)	0.426	0.961	(0.621 to 1.293)
BWS-MLMD4	cg06147822	20	57466905	-3.367	0.237	-3.612	-1.021	0.156	15.6	(8.504 to 24.699)	0.433	-1.031	(-1.372 to -0.684)
TND-MLMD2	cg06147822	20	57466905	-3.299	0.225	-3.61	-1.365	0.089	8.9	(3.892 to 16.197)	0.36	-1.379	(-1.763 to -0.986)
BWS-MLMD4	cg13895966	20	57467268	-2.917	0.265	-2.962	-0.167	0.434	43.4	(32.754 to 54.444)	0.495	-0.168	(-0.447 to 0.112)
TND-MLMD2	cg13895966	20	57467268	-2.78	0.221	-3.225	-1.999	0.026	2.6	(0.619 to 6.301)	0.229	-2.019	(-2.501 to -1.530)
BWS-MLMD4	cg21971807	20	57471654	1.947	0.197	1.674	-1.378	0.087	8.7	(3.768 to 15.921)	0.378	-1.392	(-1.778 to -0.998)
TND-MLMD2	cg21971807	20	57471654	2.105	0.212	2.119	0.061	0.476	47.6	(36.738 to 58.554)	0.497	0.062	(-0.216 to 0.339)
BWS-MLMD4	cg11021321	20	57471660	1.79	0.176	1.279	-2.868	0.003	0.3	(0.021 to 1.197)	0.1	-2.897	(-3.529 to -2.258)
TND-MLMD2	cg11021321	20	57471660	1.77	0.23	1.585	-0.797	0.215	21.5	(13.097 to 31.466)	0.444	-0.805	(-1.122 to -0.483)
BWS-MLMD4	cg05297437	20	57471672	2.244	0.19	1.846	-2.066	0.022	2.2	(0.494 to 5.629)	0.241	-2.087	(-2.580 to -1.587)
TND-MLMD2	cg05297437	20	57471672	2.141	0.167	2.091	-0.296	0.384	38.4	(28.055 to 49.426)	0.486	-0.299	(-0.581 to -0.014)
BWS-MLMD4	cg00940140	20	57480494	2.478	0.278	2.315	-0.583	0.281	28.1	(18.761 to 38.759)	0.474	-0.589	(-0.887 to -0.286)
TND-MLMD2	cg00940140	20	57480494	2.647	0.258	2.372	-1.052	0.149	14.9	(7.980 to 23.848)	0.413	-1.062	(-1.406 to -0.711)
BWS-MLMD4	cg14208013	20	57485765	3.145	0.245	3.427	1.144	0.129	12.9	(6.542 to 21.395)	0.417	1.155	(0.793 to 1.511)
TND-MLMD2	cg14208013	20	57485765	3.165	0.177	3.426	1.457	0.076	7.6	(3.076 to 14.310)	0.343	1.472	(1.067 to 1.870)
BWS-MLMD4	cg13353325	20	57485837	3.172	0.38	2.943	-0.596	0.277	27.7	(18.371 to 38.282)	0.473	-0.602	(-0.901 to -0.298)
TND-MLMD2	cg13353325	20	57485837	3.23	0.303	3.109	-0.393	0.348	34.8	(24.713 to 45.724)	0.48	-0.397	(-0.684 to -0.107)
BWS-MLMD4	cg20083839	20	57485940	1.705	0.345	1.494	-0.605	0.274	27.4	(18.124 to 37.979)	0.473	-0.611	(-0.911 to -0.306)
TND-MLMD2	cg20083839	20	57485940	1.776	0.203	1.73	-0.221	0.413	41.3	(30.754 to 52.332)	0.49	-0.223	(-0.503 to 0.058)
BWS-MLMD4	cg26452915	20	57486076	1.188	0.259	1.85	2.531	0.007	0.7	(0.088 to 2.403)	0.152	2.556	(1.977 to 3.128)
TND-MLMD2	cg26452915	20	57486076	1.538	0.197	1.835	1.491	0.071	7.1	(2.813 to 13.655)	0.336	1.506	(1.096 to 1.909)
